# Spatiotemporal patterns of rheumatic heart disease burden attributable to high systolic blood pressure, high sodium diet, and lead exposure (1990 to 2019): a longitudinal observational study

**DOI:** 10.3389/fnut.2024.1419349

**Published:** 2024-09-26

**Authors:** Yanli Zhang, Jun Zhang, Yonggang Liu, Yuzhe Zhou, Lu Ye, Kaiming Chen, Jinghua Jiao

**Affiliations:** ^1^Department of General Practice, Central Hospital Affiliated to Shenyang Medical College, Shenyang, China; ^2^Department of Thoracic Surgery, The Second People's Hospital of Weifang, Weifang, China; ^3^Graduate School, China Medical University, Shenyang, China; ^4^School of Ophthalmology and Optometry, Wenzhou Medical University, Wenzhou, China; ^5^Department of Anesthesiology, Guangzhou Eighth People’s Hospital, Guangzhou Medical University, Guangzhou, China; ^6^Development of Cardiology, Central Hospital Affiliated to Shenyang Medical College, Shenyang, China

**Keywords:** age-period-cohort model, rheumatic heart disease, global burden, risk factors, socio-demographic index

## Abstract

**Background:**

Rheumatic heart disease (RHD) continues to be a significant global health concern, exhibiting unique regional disparities. Although there is a noted decline in the burden of RHD, the specific causatives for this decrease remain unclear. This study aims to identify and quantify the spatiotemporal patterns related to the RHD-attributable risk burden.

**Methods:**

The data pertaining to deaths and disability-adjusted life years (DALYs) attributable to RHD risk were drawn from the Global Burden of Disease (GBD) study conducted from 1990 to 2019. These data, categorized by age, gender, and geographical location, highlighted risk factors including diets high in sodium, elevated systolic blood pressure (SBP), and lead exposure. To examine the long-term trends in RHD changes due to these specific risk factors, the average annual percentage change (AAPC) method was used.

**Results:**

During the past 30 years, the highest decrease in RHD burden was attributed to high SBP. An AAPC of −2.73 [95% confidence interval (CI): −2.82 to −2.65] and − 2.45 (95% CI: −2.55 to −2.36) in deaths and DALYs was attributable to high SBP, while an AAPC of −3.99 (95% CI: −4.14 to −3.85) and − 3.74 (95% CI: −3.89 to −3.6) in deaths and DALYs was attributed to a diet high in sodium. Moreover, the trends in deaths and DALYs due to lead exposure also showed decreases with an AAPC of −2.94 (95% CI: −3 to −2.89) and − 3.46 (95% CI: −3.58 to −3.34) from 1990 to 2019. Oceania showed an upward trend of the RHD DALYs due to high SBP, with an AAPC of 0.23 (95% CI: 0.13 to 0.33). In general, countries in Oceania, East Asia, and South Asia had higher age-standard deaths and DALY rates of RHD due to diets high in sodium.

**Conclusion:**

Our study has revealed that high SBP remains the prime risk factor contributing to the RHD burden. There are decreasing spatiotemporal patterns in RHD-related deaths and burdens. Gaining this knowledge is fundamental to making informed public health strategies and clinical decisions, especially concerning risk assessment, screening, and prevention initiatives.

## Introduction

Rheumatic heart disease (RHD) is indeed a serious global health issue, resulting from acute rheumatic fever and leading to long-term heart complications. It poses a significant challenge, particularly in low- and middle-income regions such as South Asia and Sub-Saharan Africa (1, 2). According to a previous report, the DALYs for RHD were 10,673,882 in 2019 (3). As for mortality, the number of deaths due to RHD was approximately 0.31 million in 2019, underscoring its continued global impact (3).

Generally, there were many factors associated with the development of RHD, including overcrowding and poor housing, socioeconomic factors, improper health literacy, genetic background, and even inequity in healthcare (4, 5). Moreover, many metabolic, environmental, and behavioral factors, such as a diet high in sodium, high SBP, and lead exposure, are also associated with RHD (6). While RHD primarily results from an autoimmune reaction following streptococcal infections, emerging evidence suggests that elevated blood pressure, high sodium intake, and lead exposure can exacerbate cardiovascular damage and potentially influence the course of RHD. These factors May contribute to systemic inflammation, endothelial dysfunction, and overall cardiovascular burden, which can indirectly affect individuals with RHD. However, there has been no comprehensive estimation of the RHD-related burden attributable to these factors.

To the best of our understanding, the GBD is the sole dataset that provides a detailed view of the disease burden due to a particular set of risk factors. The database uses rigorous quality assurance procedures and presents evolving models across various scales over time. In addition, GBD 2019 furnishes the most recent data on each risk-outcome pair for cardiovascular diseases (CVDs) in 204 nations and territories, including their aggregated regions, spanning from 1990 to 2019. This provision facilitates the assessment of the burden attributable to risks for countries with low-income economies. Additionally, robust epidemiological evidence, such as a comprehensive spatiotemporal assessment of the RHD-related burden associated with these factors, would aid the development of effective prevention strategies that reduce the disease burden. Herein, we conducted this comparative study to estimate the burden and trends of RHD attributable to a diet high in sodium, elevated SBP, and lead exposure among different populations at global, regional, and national levels using the latest data from the GBD dataset, aiming to provide a comprehensive basis for the robust, evidence-based development of disease prevention strategies.

## Methods

### Data source

Data on the burden of RHD attributable to a diet high in sodium, high SBP, and lead exposure–across global, regional, and national levels, stratified by age and sex–were retrieved from the GBD 2019 datasets released by the Institute for Health Metrics and Evaluation (IHME). The GBD 2019 study used DisMod-MR 2.1, a Bayesian meta-regression tool, to estimate disease-related measures, including disability, across 369 diseases and injuries and 87 risk factors in 204 countries and territories over the past three decades (7). The population and data utilized in our study are derived from the GBD database, which aggregates data from a wide range of sources, including vital registration systems, health surveys, hospital records, and scientific literature. The GBD database covers nearly every country and region worldwide and systematically collects data to estimate disease burden over time. For our study, we analyzed data spanning from 1990 to 2019, focusing on the RHD burden attributable to high SBP, high sodium diet, and lead exposure. While the GBD study provides extensive global coverage, individual-level data or specific cohort details are not available as it uses aggregated data to generate estimates at the population level. This allows for a comprehensive analysis of disease burden trends over time across different regions and populations. In this study, the burden indicators of RHD included mortality numbers (X 1,000), DALY numbers (X 1,000), the age-standardized mortality rate (per 100,000), and the age-standardized DALY rate (per 100,000). Comprehensive methodology and statistical modeling for the GBD 2019 have been published elsewhere (7, 8). Briefly, the dietary intake of sodium was evaluated by calculating the average 24-h urinary sodium excretion, which was specified in grams per day. High dietary sodium was defined as an excretion exceeding 1–5 grams per day. High SBP was identified through the measurement of brachial SBP, reported in mmHg. The benchmark data used for mean SBP were derived from literary sources and household survey data, including reports such as STEPS and NHANES as per the GBD 2019. Regarding lead exposure, it has been distinguished in two separate ways, each corresponding to its unique pathway leading to attributable health losses. Acute lead exposure was measured in micrograms of lead per deciliter of blood (μg/dL), a condition known to be associated with IQ reduction in children. On the other hand, chronic lead exposure was measured in micrograms of lead per gram of bone (μg/g), a metric associated with elevated SBP and increased incidence of CVDs.

GBD study has been approved by the University of Washington Institutional Review Board. All CVDs were identified by the International Classification of Diseases and Injuries (ICD), 10th Revision.

The sociodemographic index (SDI) serves as a comprehensive assessment of health-related development within a given region or country. This composite indicator is calculated based on fertility rates, *per capita* income, and the level of educational attainment. The SDI values extend from 0 to 1, with low values indicating a low degree of health-related development and high values indicating a high degree of health development at both national and regional levels.

Based on the quintiles of the SDI, all countries are categorized into one of the five SDI zones, namely: low-SDI, low-middle-SDI, middle-SDI, high-middle-SDI, and high-SDI regions. This classification provides a structured framework for comparative analysis of health-related development across different regions and nations worldwide.

### Statistical analysis

The mortality and DALY rates per 100,000 population, inclusive of their 95% uncertainty intervals (UIs), attributed to RHD, are outlined by both age and gender. We examined the temporal trend shifts of RHD mortality and DALYs linked to a diet high in sodium, high SBP, and lead exposure through Joinpoint regression analysis (version 4.9.0.1 from the Statistical Research and Applications Branch, National Cancer Institute).

To depict the temporal trends of RHD mortality attributable to a diet high in sodium, high SBP, and lead exposure, we computed the average annual percent change (AAPC) and its corresponding 95% CI utilizing the Monte Carlo substitution method. We interpreted an increasing trend if the AAPC and its 95% CI exceeded zero. Conversely, an AAPC and its 95% CI of less than zero signified a decreasing trend. Any other values indicated a stable trend.

## Results

### Burden of RHD attributable to high SBP

**Figure 1:**
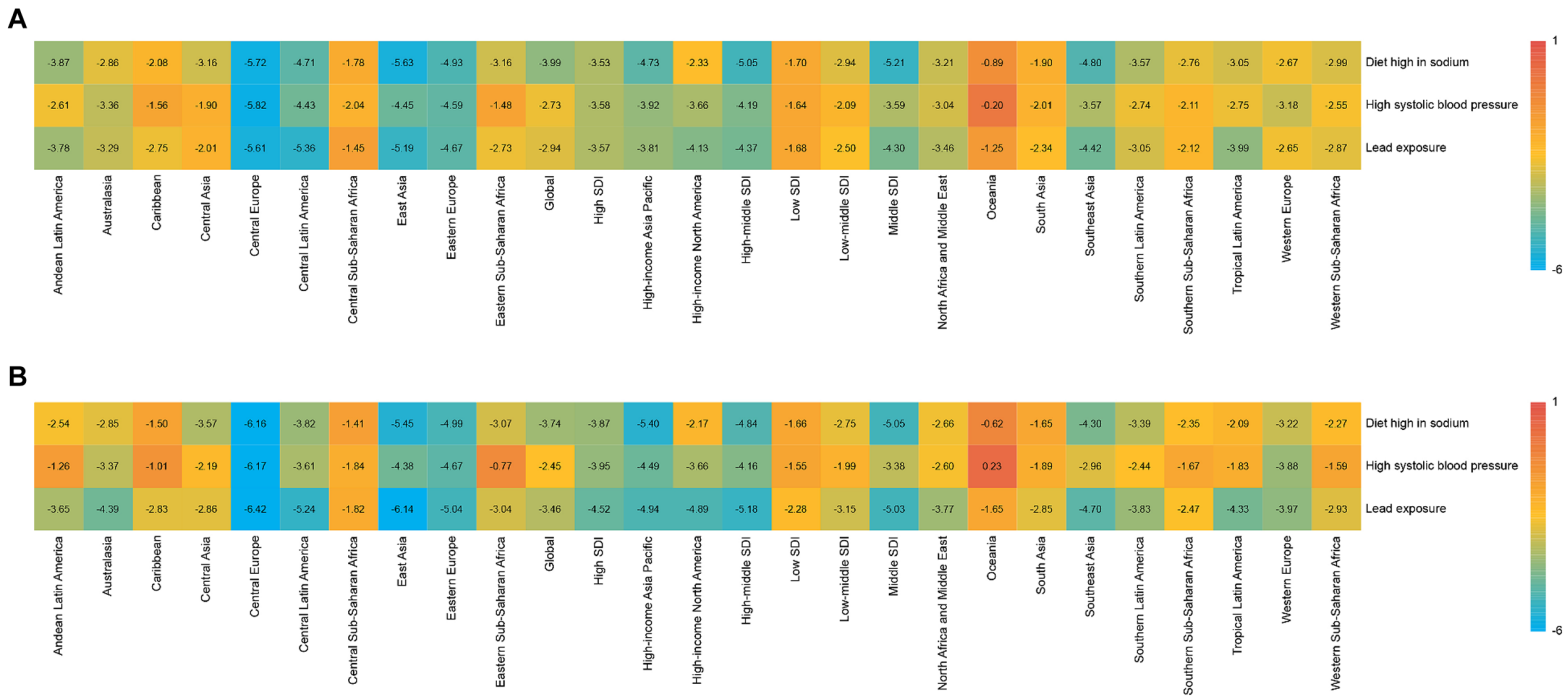
Average annual percentage change for rheumatic heart disease risk-attributable age-standardized mortality rate and age-standardized disability-adjusted life year (DALY) rate in 21 Global Burden of Disease regions classified by five sociodemographic index levels for both sexes in 1990–2019. SDI: sociodemographic index. **(A)** Mortality; **(B)** DALY.

Globally in 2019, there was a total number of 76.4 (51 to 114.7) deaths and 2446.4 (1672.9 to 3445.3) DALYs attributable to high SBP-related RHDs (Table 1). The corresponding rates were 1 death and 29.7 DALYs per 100,000 population (Table 1). The top three regions with high death rates were mainly in Oceania, South Asia, and Central Asia (Table 1), and the highest record of death rate and DALYs rate was observed in Vanuatu (4.9 per 100,000) and Indonesia (174.4 per 100,000) in 2019, respectively (Supplementary Tables S1, S2). From 1990 to 2019, the rates of RHD deaths and DALYs associated with high SBP showed decreasing trends (Figure 1). There was a spatially heterogeneous burden of RHD due to high SBP. Countries in high-income Asia Pacific, Eastern Europe, and Central Europe had the most significant decline, and Singapore, Hungary, and Poland were the top three countries that had fallen the furthest (Supplementary Tables S1, S2). Notably, Oceania was the only region to show an upward trend in RHD DALYs due to high SBP, with an AAPC of 0.23 (95%CI: 0.13 to 0.33), while the death rate showed a downward trend, with an AACP of −0.2 (95%CI: −0.28 to −0.11).

**Table 1:** Global burden of rheumatic heart disease burden attributable to high systolic blood pressure, diet high in sodium, and lead exposure from 1990 to 2019.

Attributable factors	1990				2019			
	Death (X1000, persons, 95% UI)	ASRM (95% UI)	DALY (X1000, persons, 95% UI)	ASRD (95% UI)	Death (X1000, persons, 95% UI)	ASRM (95% UI)	DALY (X1000, persons, 95% UI)	ASRD (95% UI)
High systolic blood pressure								
Global	84 (56 to 127.7)	2.1 (1.4 to 3.3)	2677.4 (1784.6 to 3,904)	61.2 (41.1 to 89.8)	76.4 (51 to 114.7)	1 (0.6 to 1.4)	2446.4 (1672.9 to 3445.3)	29.7 (20.3 to 41.9)
High SDI	8.3 (5.3 to 13.8)	0.8 (0.5 to 1.3)	180.7 (123.7 to 276.8)	17.7 (12 to 26.5)	6 (3.3 to 11.7)	0.3 (0.2 to 0.5)	101.2 (65.3 to 174.3)	5.5 (3.7 to 8.9)
High-middle SDI	17.9 (12.2 to 27.2)	1.7 (1.2 to 2.6)	537.5 (375.1 to 781.3)	48 (33.7 to 71.5)	9.8 (6.4 to 16)	0.5 (0.3 to 0.8)	272.8 (186.3 to 408.3)	14 (9.7 to 21.1)
Middle SDI	26.2 (17 to 42.3)	2.7 (1.7 to 4.6)	824.5 (534.2 to 1225.5)	69.4 (45.8 to 109.7)	21.4 (14.3 to 33.7)	0.9 (0.6 to 1.5)	669 (460.2 to 987.4)	26.1 (17.6 to 38)
Low-middle SDI	23.4 (15.1 to 35.5)	3.8 (2.4 to 5.9)	828.4 (519.6 to 1,225)	111.4 (69.7 to 167.7)	28 (18.3 to 40.7)	2.1 (1.3 to 3.1)	965 (623.1 to 1347.3)	62.2 (41.1 to 87.6)
Low SDI	8.2 (4.9 to 13.3)	3.3 (2 to 5.6)	305.3 (186.2 to 481)	100.3 (61.2 to 161.1)	11.1 (7.3 to 16.4)	2.1 (1.3 to 3.2)	437.1 (283.9 to 621.2)	63.9 (42 to 91.4)
Andean Latin America	0.1 (0.1 to 0.2)	0.5 (0.3 to 0.8)	3.7 (2.1 to 6.4)	14.8 (8.9 to 25.8)	0.1 (0.1 to 0.2)	0.2 (0.1 to 0.4)	6.3 (3.8 to 10.5)	10.3 (6.3 to 17.1)
Australasia	0.2 (0.1 to 0.3)	0.7 (0.5 to 1.2)	3.9 (2.7 to 6)	16.8 (11.5 to 25.5)	0.1 (0.1 to 0.3)	0.3 (0.2 to 0.5)	2.8 (1.8 to 4.7)	6.2 (4.2 to 9.8)
Caribbean	0.3 (0.2 to 0.5)	1.1 (0.7 to 1.7)	13.3 (7.8 to 21)	44 (26.3 to 70.6)	0.3 (0.2 to 0.5)	0.7 (0.4 to 1.1)	16.2 (9.4 to 25.3)	32.2 (18.6 to 50.7)
Central Asia	1.2 (0.8 to 1.7)	2.2 (1.5 to 3.1)	48.6 (32.3 to 69.3)	85.9 (58.5 to 123)	1 (0.7 to 1.5)	1.2 (0.8 to 1.8)	41.7 (27.1 to 61.1)	44.7 (29.8 to 64.7)
Central Europe	3.1 (2.1 to 4.5)	2.1 (1.5 to 3)	91.5 (64.6 to 129.6)	62.4 (44.7 to 88.9)	0.8 (0.5 to 1.3)	0.4 (0.2 to 0.6)	19.2 (12.9 to 29.1)	10 (6.8 to 14.8)
Central Latin America	0.6 (0.4 to 1)	0.7 (0.5 to 1)	24.8 (15.7 to 38.2)	22.9 (15.4 to 36.5)	0.4 (0.3 to 0.6)	0.2 (0.1 to 0.3)	20.2 (13.5 to 31)	8 (5.4 to 12.5)
Central Sub-Saharan Africa	0.5 (0.3 to 0.8)	2 (1.1 to 3.4)	20.4 (11.9 to 31.1)	65.9 (40.9 to 103.9)	0.6 (0.3 to 1)	1.1 (0.6 to 2)	29.3 (16.9 to 47.9)	38.5 (22.5 to 63.6)
East Asia	27.8 (16.9 to 47)	3.7 (2.2 to 6.7)	763.5 (481.6 to 1180.3)	82.4 (51.6 to 134.5)	18.8 (11.7 to 30.1)	1 (0.6 to 1.7)	464.5 (304.1 to 706.9)	23.1 (15.2 to 35.3)
Eastern Europe	4.8 (3.3 to 7.2)	1.7 (1.2 to 2.5)	165.6 (117.3 to 239.5)	59.7 (42.4 to 87.4)	1.4 (1 to 2.2)	0.4 (0.3 to 0.7)	47.1 (31.8 to 70.2)	14.9 (10.3 to 21.8)
Eastern Sub-Saharan Africa	0.7 (0.4 to 1)	0.9 (0.5 to 1.4)	33 (19.7 to 49.2)	31.4 (19.8 to 47)	0.9 (0.6 to 1.4)	0.6 (0.4 to 1)	64.8 (38.3 to 100.5)	25.1 (16.2 to 37.7)
High-income Asia Pacific	1.1 (0.7 to 1.7)	0.6 (0.4 to 1)	22.4 (15.4 to 33.5)	11.4 (7.9 to 17.3)	1.1 (0.5 to 2.4)	0.2 (0.1 to 0.4)	14.6 (8.6 to 28.1)	3 (2 to 5.2)
High-income North America	2.4 (1.5 to 4)	0.7 (0.4 to 1.1)	52.1 (34.7 to 81.7)	15.1 (10.2 to 23.2)	1.5 (0.8 to 2.9)	0.2 (0.1 to 0.4)	30.8 (19.4 to 52.6)	5.1 (3.3 to 8.5)
North Africa and Middle East	1.9 (1.1 to 3.5)	1 (0.6 to 2.1)	72.3 (43.2 to 124.3)	32.9 (20 to 58.1)	1.9 (1.2 to 3.1)	0.4 (0.3 to 0.7)	84.9 (54.3 to 134.1)	15.3 (10 to 24.6)
Oceania	0.1 (0 to 0.2)	2.9 (1.4 to 5.2)	3.8 (1.8 to 6.7)	90 (44.7 to 159.4)	0.2 (0.1 to 0.4)	2.7 (1.4 to 4.7)	10.2 (4.9 to 17.7)	96.4 (47.8 to 163.6)
South Asia	28.6 (18 to 43.3)	4.9 (3.1 to 7.8)	1038.7 (647.8 to 1552.7)	142.3 (90.4 to 216.5)	38.4 (24.9 to 55)	2.7 (1.8 to 4.1)	1309.4 (851.4 to 1839.4)	81.5 (54 to 113.7)
Southeast Asia	2.4 (1.5 to 3.6)	0.9 (0.6 to 1.5)	90.8 (55 to 135.7)	27.1 (17.4 to 42.1)	1.8 (1.2 to 2.6)	0.3 (0.2 to 0.5)	77.4 (50.5 to 113.5)	11.3 (7.5 to 16.5)
Southern Latin America	0.5 (0.3 to 0.8)	1.2 (0.7 to 2.1)	12.5 (8.2 to 19.8)	27.1 (18 to 43)	0.4 (0.3 to 0.8)	0.5 (0.3 to 1)	10.5 (6.8 to 16.4)	13.3 (8.6 to 20.8)
Southern Sub-Saharan Africa	0.4 (0.2 to 0.5)	1.1 (0.7 to 1.6)	19.3 (11.4 to 28.5)	48.7 (30.3 to 70)	0.4 (0.2 to 0.5)	0.6 (0.4 to 0.9)	22.8 (13.7 to 34.2)	30.2 (19.2 to 44.4)
Tropical Latin America	0.7 (0.5 to 1.1)	0.7 (0.5 to 1.1)	36.4 (22.2 to 56.6)	29.6 (19.4 to 46.7)	0.7 (0.5 to 1.1)	0.3 (0.2 to 0.4)	42.8 (27.2 to 66.4)	17 (10.8 to 26.5)
Western Europe	5.7 (3.7 to 9.4)	1 (0.6 to 1.6)	116.9 (78.9 to 179.5)	20.9 (14.3 to 31.3)	4.2 (2.2 to 8.2)	0.4 (0.2 to 0.7)	61 (37.4 to 107.9)	6.6 (4.3 to 10.6)
Western Sub-Saharan Africa	1.2 (0.7 to 2.2)	1.5 (0.9 to 2.7)	43.8 (26.3 to 73.8)	41.5 (25.2 to 71.6)	1.3 (0.8 to 2.1)	0.7 (0.4 to 1.2)	69.9 (43.4 to 116.1)	25.9 (17 to 41.9)
Diet high in sodium								
Global	19.1 (6.4 to 42.3)	0.5 (0.2 to 1)	624.1 (213.1 to 1323.9)	14.2 (4.8 to 30.3)	11.9 (2.8 to 29.1)	0.1 (0 to 0.4)	390.8 (98.1 to 930.5)	4.7 (1.2 to 11.2)
High SDI	0.8 (0.2 to 2.2)	0.1 (0 to 0.2)	20.2 (5.3 to 49.6)	2 (0.5 to 4.9)	0.6 (0.1 to 2)	0 (0 to 0.1)	11.5 (2 to 32.2)	0.6 (0.1 to 1.7)
High-middle SDI	4.4 (1.5 to 9.1)	0.4 (0.1 to 0.8)	136 (50 to 271.7)	12.1 (4.4 to 24)	1.9 (0.6 to 4.1)	0.1 (0 to 0.2)	57.8 (19.9 to 120)	2.9 (1 to 6.1)
Middle SDI	9.1 (3.5 to 18.1)	0.9 (0.3 to 1.8)	296.2 (120.3 to 572.5)	24.4 (9.6 to 47.9)	4.6 (1.3 to 10)	0.2 (0.1 to 0.4)	145.9 (47.5 to 308)	5.6 (1.8 to 12)
Low-middle SDI	4 (1 to 9.7)	0.6 (0.2 to 1.6)	138.7 (33.6 to 335)	18.8 (4.7 to 45.8)	3.7 (0.6 to 9.9)	0.3 (0 to 0.7)	130.6 (21.8 to 345.2)	8.4 (1.4 to 22.2)
Low SDI	0.9 (0.1 to 2.7)	0.4 (0 to 1.1)	32.9 (3 to 103.2)	10.9 (1 to 34.1)	1.2 (0.1 to 3.5)	0.2 (0 to 0.7)	44.9 (4.4 to 136.7)	6.7 (0.7 to 19.8)
Andean Latin America	0 (0 to 0.1)	0.1 (0 to 0.3)	0.8 (0.1 to 2.3)	3 (0.2 to 9)	0 (0 to 0)	0 (0 to 0.1)	0.9 (0.1 to 2.7)	1.4 (0.1 to 4.3)
Australasia	0 (0 to 0)	0 (0 to 0.1)	0.2 (0 to 0.7)	0.9 (0.1 to 3.2)	0 (0 to 0)	0 (0 to 0.1)	0.2 (0 to 0.6)	0.4 (0.1 to 1.4)
Caribbean	0 (0 to 0.1)	0.1 (0 to 0.2)	0.8 (0.1 to 3)	2.6 (0.3 to 9.9)	0 (0 to 0.1)	0 (0 to 0.1)	0.8 (0.1 to 3.2)	1.7 (0.2 to 6.4)
Central Asia	0.2 (0 to 0.4)	0.3 (0.1 to 0.7)	6.7 (1.3 to 16.4)	12.1 (2.3 to 28.8)	0.1 (0 to 0.3)	0.1 (0 to 0.3)	3.9 (0.5 to 11)	4.2 (0.5 to 11.9)
Central Europe	0.7 (0.2 to 1.4)	0.5 (0.2 to 0.9)	19.8 (6.8 to 38.4)	13.6 (4.8 to 26.5)	0.2 (0.1 to 0.4)	0.1 (0 to 0.2)	4.3 (1.1 to 8.9)	2.2 (0.6 to 4.6)
Central Latin America	0.1 (0 to 0.2)	0.1 (0 to 0.2)	3.2 (0.4 to 9.1)	2.9 (0.3 to 8.1)	0 (0 to 0.1)	0 (0 to 0.1)	2.4 (0.3 to 6.7)	0.9 (0.1 to 2.6)
Central Sub-Saharan Africa	0 (0 to 0.1)	0.1 (0 to 0.4)	1.1 (0.1 to 4.6)	3.3 (0.4 to 14)	0 (0 to 0.1)	0.1 (0 to 0.2)	1.8 (0.2 to 8)	2.2 (0.2 to 9.8)
East Asia	12.7 (5.1 to 25)	1.5 (0.6 to 3)	398.5 (172.7 to 734.6)	40 (16.9 to 76.7)	5.7 (2 to 11.8)	0.3 (0.1 to 0.6)	168.3 (69.7 to 314.6)	8.1 (3.3 to 15.2)
Eastern Europe	0.5 (0.1 to 1.2)	0.2 (0 to 0.4)	16.3 (2 to 43)	5.9 (0.7 to 15.5)	0.1 (0 to 0.4)	0 (0 to 0.1)	4.2 (0.5 to 11.5)	1.3 (0.2 to 3.6)
Eastern Sub-Saharan Africa	0.2 (0 to 0.5)	0.2 (0 to 0.7)	7.2 (0.5 to 22.4)	7.5 (0.6 to 21.6)	0.1 (0 to 0.4)	0.1 (0 to 0.3)	6.6 (0.5 to 24.6)	3.1 (0.2 to 10.1)
High-income Asia Pacific	0.2 (0.1 to 0.5)	0.1 (0 to 0.3)	5.4 (1.6 to 11.3)	2.7 (0.8 to 5.7)	0.2 (0 to 0.6)	0 (0 to 0.1)	2.6 (0.4 to 7)	0.5 (0.1 to 1.4)
High-income North America	0.1 (0 to 0.5)	0 (0 to 0.1)	3.6 (0.4 to 12)	1.1 (0.1 to 3.6)	0.1 (0 to 0.5)	0 (0 to 0.1)	3.3 (0.4 to 9.8)	0.6 (0.1 to 1.6)
North Africa and Middle East	0.1 (0 to 0.3)	0 (0 to 0.1)	3 (0.6 to 11.5)	1.3 (0.3 to 5)	0.1 (0 to 0.3)	0 (0 to 0.1)	3.5 (0.7 to 12.9)	0.6 (0.1 to 2.3)
Oceania	0 (0 to 0)	0.6 (0.1 to 1.7)	0.4 (0.1 to 1.4)	12.8 (1.9 to 39.9)	0 (0 to 0.1)	0.4 (0 to 1.3)	0.9 (0.1 to 3.2)	10.8 (1.3 to 34.9)
South Asia	3.2 (0.3 to 9.4)	0.5 (0.1 to 1.6)	117.7 (12.1 to 341.1)	15.9 (1.6 to 46.9)	4.3 (0.5 to 12.4)	0.3 (0 to 0.9)	157.8 (17.5 to 440.4)	9.6 (1.1 to 27.1)
Southeast Asia	0.6 (0.1 to 1.2)	0.2 (0.1 to 0.5)	20.4 (4.8 to 44.9)	6.5 (1.6 to 13.9)	0.3 (0 to 0.8)	0.1 (0 to 0.1)	12.4 (1.7 to 31.5)	1.8 (0.2 to 4.6)
Southern Latin America	0.1 (0 to 0.2)	0.2 (0 to 0.5)	1.9 (0.2 to 5.3)	4.1 (0.4 to 11.5)	0 (0 to 0.2)	0.1 (0 to 0.2)	1.2 (0.1 to 3.6)	1.5 (0.1 to 4.4)
Southern Sub-Saharan Africa	0 (0 to 0.1)	0.1 (0 to 0.4)	2 (0.1 to 7.4)	4.8 (0.3 to 18)	0 (0 to 0.1)	0 (0 to 0.2)	1.9 (0.1 to 7.4)	2.5 (0.2 to 9.5)
Tropical Latin America	0.1 (0 to 0.2)	0.1 (0 to 0.2)	3.9 (0.3 to 11.8)	3.3 (0.3 to 9.4)	0.1 (0 to 0.2)	0 (0 to 0.1)	4.4 (0.4 to 13.3)	1.7 (0.2 to 5.3)
Western Europe	0.3 (0 to 1.1)	0.1 (0 to 0.2)	7.1 (1 to 23)	1.3 (0.2 to 4.2)	0.3 (0 to 1)	0 (0 to 0.1)	4.3 (0.6 to 13.7)	0.5 (0.1 to 1.6)
Western Sub-Saharan Africa	0.1 (0 to 0.4)	0.1 (0 to 0.5)	4.1 (0.3 to 16.3)	3.8 (0.3 to 14.8)	0.1 (0 to 0.4)	0.1 (0 to 0.2)	5.2 (0.4 to 21.5)	2 (0.2 to 7.7)
Lead exposure								
Global	9.4 (5.1 to 16.1)	0.2 (0.1 to 0.4)	320.2 (168.9 to 543.6)	7.2 (3.9 to 12.2)	7.7 (4.1 to 13.5)	0.1 (0 to 0.2)	213.5 (106.8 to 372.7)	2.6 (1.3 to 4.6)
High SDI	0.2 (0.1 to 0.5)	0 (0 to 0)	5 (1.1 to 10.7)	0.5 (0.1 to 1.1)	0.2 (0 to 0.4)	0 (0 to 0)	2.6 (0.4 to 6.1)	0.1 (0 to 0.3)
High-middle SDI	1.1 (0.5 to 2.2)	0.1 (0.1 to 0.2)	33.4 (14.9 to 63)	3 (1.4 to 5.6)	0.6 (0.3 to 1.2)	0 (0 to 0.1)	13.1 (5.5 to 25.4)	0.7 (0.3 to 1.3)
Middle SDI	3.1 (1.6 to 5.5)	0.3 (0.2 to 0.6)	100.4 (49.8 to 172.9)	8.3 (4.3 to 14.9)	1.9 (0.9 to 3.4)	0.1 (0 to 0.2)	46.3 (21.8 to 84.7)	1.9 (0.9 to 3.4)
Low-middle SDI	3.7 (2.1 to 6.2)	0.6 (0.3 to 1)	134.4 (73.4 to 224.9)	17.9 (10.1 to 29.1)	3.6 (1.9 to 6)	0.3 (0.1 to 0.5)	103.4 (51 to 177)	7 (3.6 to 11.9)
Low SDI	1.3 (0.7 to 2.3)	0.5 (0.3 to 0.9)	46.9 (23.8 to 81.7)	15.3 (8 to 26.7)	1.5 (0.8 to 2.6)	0.3 (0.2 to 0.5)	48.1 (24 to 82.7)	7.8 (4.2 to 13.4)
Andean Latin America	0 (0 to 0)	0 (0 to 0.1)	0.5 (0.1 to 1)	1.7 (0.5 to 3.6)	0 (0 to 0)	0 (0 to 0)	0.4 (0.1 to 0.9)	0.6 (0.1 to 1.4)
Australasia	0 (0 to 0)	0 (0 to 0.1)	0.2 (0.1 to 0.4)	0.9 (0.3 to 1.7)	0 (0 to 0)	0 (0 to 0)	0.1 (0 to 0.3)	0.2 (0.1 to 0.5)
Caribbean	0 (0 to 0.1)	0.1 (0 to 0.2)	1.3 (0.5 to 2.5)	4.4 (1.8 to 8.4)	0 (0 to 0)	0 (0 to 0.1)	1 (0.3 to 2.1)	1.9 (0.6 to 4.1)
Central Asia	0 (0 to 0.1)	0.1 (0 to 0.1)	1.3 (0.2 to 3.4)	2.3 (0.3 to 6)	0 (0 to 0.1)	0 (0 to 0.1)	0.9 (0.1 to 2.4)	1 (0.1 to 2.7)
Central Europe	0.1 (0 to 0.2)	0.1 (0 to 0.1)	2.3 (0.4 to 5.3)	1.6 (0.2 to 3.7)	0 (0 to 0)	0 (0 to 0)	0.5 (0.1 to 1)	0.2 (0 to 0.5)
Central Latin America	0.1 (0 to 0.1)	0.1 (0 to 0.1)	2.5 (1.1 to 4.6)	2.3 (1.1 to 4.1)	0 (0 to 0.1)	0 (0 to 0)	1.2 (0.5 to 2.4)	0.5 (0.2 to 0.9)
Central Sub-Saharan Africa	0 (0 to 0)	0.1 (0 to 0.2)	0.9 (0.2 to 2.1)	3.1 (0.9 to 6.6)	0 (0 to 0.1)	0.1 (0 to 0.1)	1.2 (0.3 to 3)	1.8 (0.5 to 4.3)
East Asia	3.6 (1.9 to 6.5)	0.4 (0.2 to 0.9)	108.9 (56 to 189.9)	11.2 (5.8 to 19.9)	1.8 (0.8 to 3.3)	0.1 (0 to 0.2)	37.4 (18.1 to 67.2)	1.8 (0.9 to 3.3)
Eastern Europe	0 (0 to 0.2)	0 (0 to 0.1)	1.6 (0 to 5.6)	0.6 (0 to 2)	0 (0 to 0)	0 (0 to 0)	0.4 (0 to 1.4)	0.1 (0 to 0.5)
Eastern Sub-Saharan Africa	0.1 (0 to 0.1)	0.1 (0 to 0.2)	2.8 (1 to 5.6)	3 (1.2 to 5.5)	0.1 (0 to 0.1)	0 (0 to 0.1)	2.4 (0.7 to 5.9)	1.2 (0.5 to 2.5)
High-income Asia Pacific	0 (0 to 0)	0 (0 to 0)	0.4 (0 to 1)	0.2 (0 to 0.5)	0 (0 to 0.1)	0 (0 to 0)	0.3 (0 to 0.7)	0 (0 to 0.1)
High-income North America	0.1 (0 to 0.2)	0 (0 to 0.1)	2 (0.5 to 4.2)	0.6 (0.1 to 1.2)	0.1 (0 to 0.1)	0 (0 to 0)	0.9 (0.1 to 2.2)	0.1 (0 to 0.3)
North Africa and Middle East	0.2 (0.1 to 0.5)	0.1 (0.1 to 0.3)	8.7 (4 to 17.7)	3.9 (1.9 to 8)	0.2 (0.1 to 0.3)	0 (0 to 0.1)	6.7 (3 to 13)	1.3 (0.6 to 2.5)
Oceania	0 (0 to 0)	0.1 (0 to 0.3)	0.1 (0 to 0.4)	2.3 (0.1 to 8.7)	0 (0 to 0)	0.1 (0 to 0.2)	0.1 (0 to 0.6)	1.4 (0 to 5.6)
South Asia	4.7 (2.6 to 7.6)	0.8 (0.4 to 1.4)	172.4 (94.4 to 290.6)	23.4 (13.2 to 38.6)	5.1 (2.8 to 8.6)	0.4 (0.2 to 0.7)	151 (77.6 to 252.7)	9.9 (5.2 to 16.4)
Southeast Asia	0.1 (0 to 0.3)	0 (0 to 0.1)	5 (1.3 to 11.2)	1.4 (0.4 to 3)	0.1 (0 to 0.1)	0 (0 to 0)	2.4 (0.5 to 6)	0.4 (0.1 to 0.9)
Southern Latin America	0 (0 to 0)	0 (0 to 0.1)	0.3 (0 to 1)	0.8 (0 to 2.1)	0 (0 to 0)	0 (0 to 0)	0.2 (0 to 0.6)	0.2 (0 to 0.7)
Southern Sub-Saharan Africa	0 (0 to 0)	0 (0 to 0.1)	0.7 (0.2 to 1.8)	1.9 (0.5 to 4.4)	0 (0 to 0)	0 (0 to 0.1)	0.7 (0.1 to 1.8)	1 (0.2 to 2.3)
Tropical Latin America	0 (0 to 0.1)	0 (0 to 0.1)	2 (0.5 to 4.5)	1.6 (0.5 to 3.4)	0 (0 to 0.1)	0 (0 to 0)	1.1 (0.2 to 3)	0.5 (0.1 to 1.2)
Western Europe	0.2 (0 to 0.3)	0 (0 to 0.1)	3.1 (0.8 to 6.7)	0.6 (0.1 to 1.2)	0.1 (0 to 0.3)	0 (0 to 0)	1.8 (0.4 to 4.2)	0.2 (0 to 0.4)
Western Sub-Saharan Africa	0.1 (0 to 0.2)	0.1 (0 to 0.2)	3.1 (1 to 6.3)	2.9 (1 to 5.9)	0.1 (0 to 0.2)	0 (0 to 0.1)	2.9 (0.9 to 6.4)	1.2 (0.5 to 2.5)

SDI, sociodemographic index.

Among SDI quintiles, the global high SBP-related RHDs burden varied worldwide. In 2019, the highest death and DALY numbers were observed by the low-middle SDI quintile (Table 1). Furthermore, the highest death and DALY rates were observed in the low-middle SDI quintiles (Table 1). The high-SDI quintile experienced the lowest burden, while the high-middle SDI quintile had the highest downward trend for both death and DALY rates over the past three decades (Figure 1).

**Figure 2:**
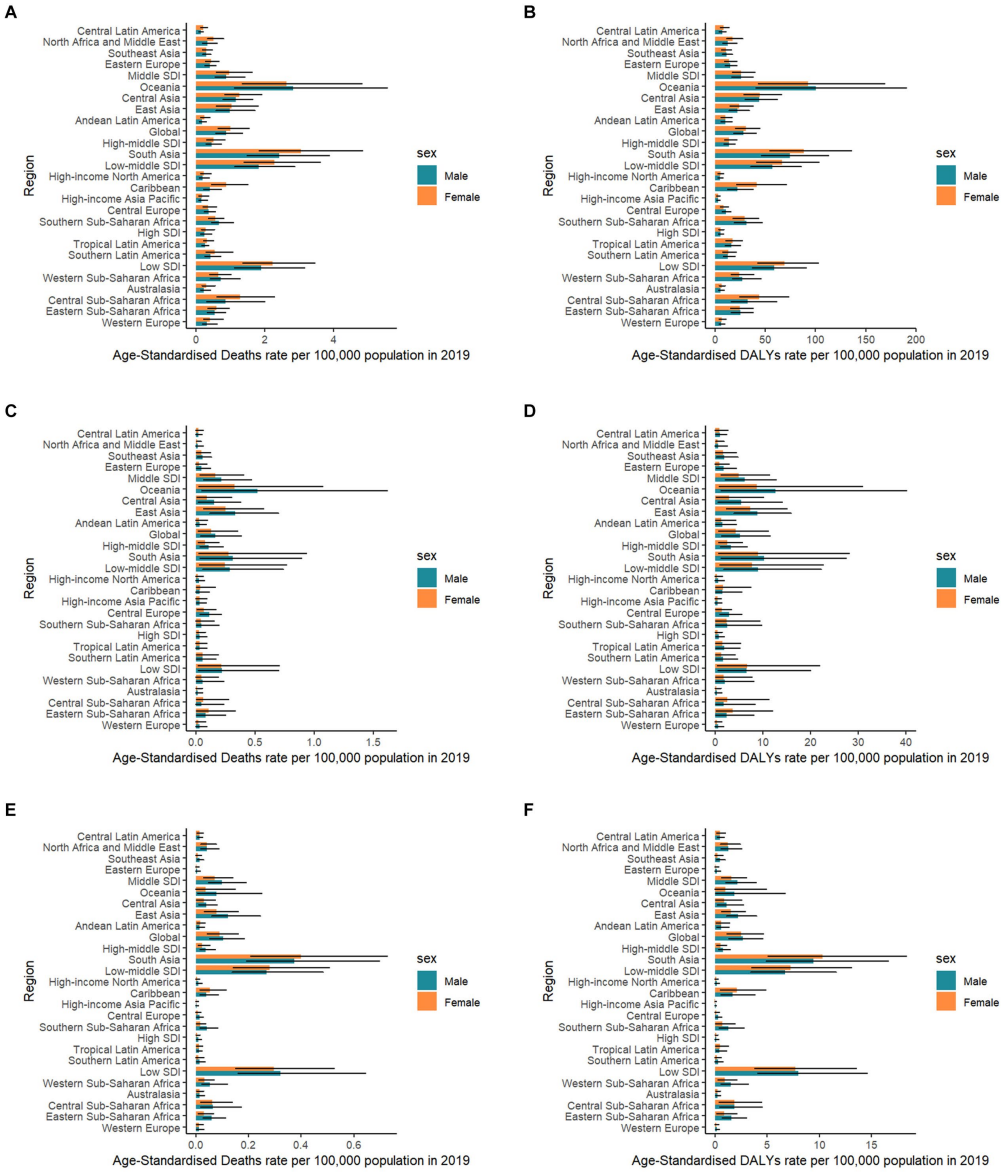
Rheumatic heart disease risk-attributable age-standardized mortality rate and age-standardized disability-adjusted life year (DALY) rate in 21 Global Burden of Disease regions classified by five sociodemographic index levels by sex in 2019. SDI: sociodemographic index. **(A)** Mortality due to high systolic blood pressure; **(B)** DALY due to high systolic blood pressure; **(C)** Mortality due to diet high in sodium; **(D)** DALY due to diet high in sodium; **(E)** Mortality due to lead exposure; and **(F)** DALY due to lead exposure.

**Figure 3:**
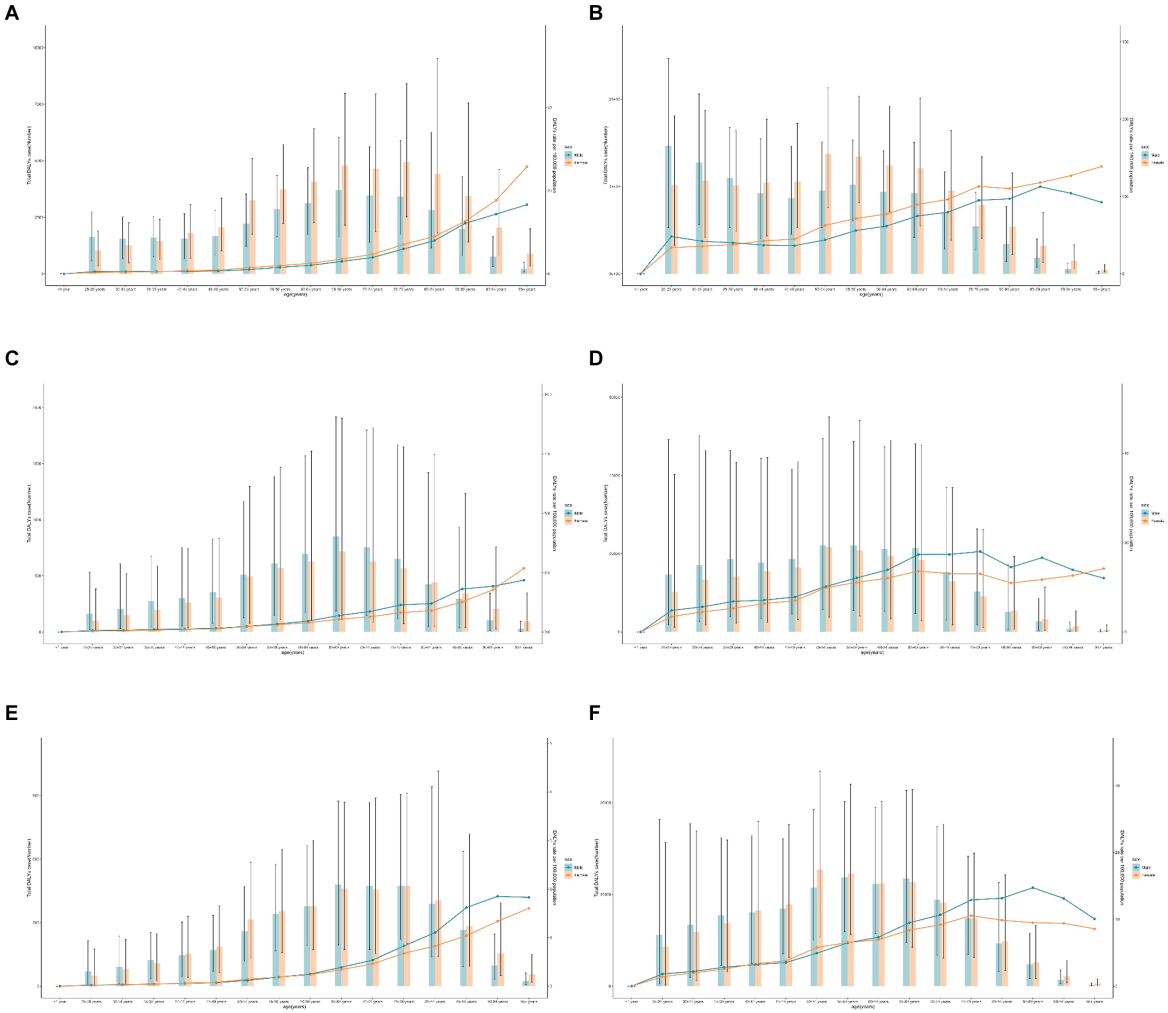
Global rheumatic heart disease risk-attributable age-standardized mortality rate and age-standardized disability-adjusted life year (DALY) rate by age and gender in 2019. **(A)** Mortality due to high systolic blood pressure; **(B)** DALY due to high systolic blood pressure; **(C)** mortality due to diet high in sodium; **(D)** DALY due to diet high in sodium; **(E)** Mortality due to lead exposure; and **(F)** DALY due to lead exposure.

**Figure 4:**
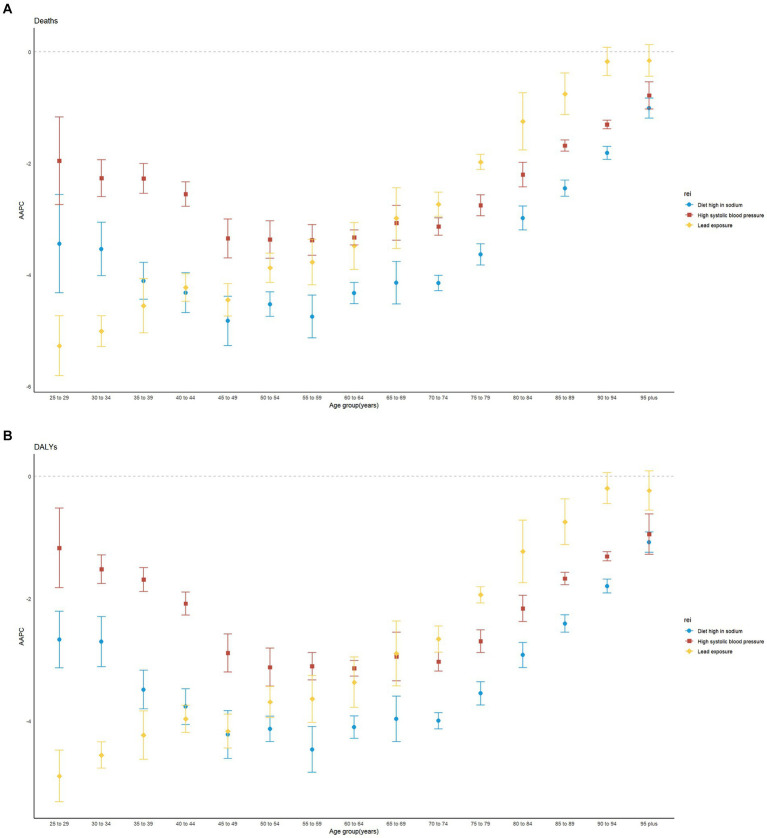
Average annual percentage change of age-standardized mortality rate and age-standardized disability-adjusted life year (DALY) rate for both sexes combined by age groups in 1990–2019 globally. AAPC: average annual percentage change. **(A)** Mortality; **(B)** DALY.

Significant gender differences existed in high SBP-related RHD burden. Females had much higher death rates than males, both globally and regionally (Figures 2A,B). In different age stratifications, males had much higher death numbers and rates than females before 40 years, while females showed higher death rates older than 40 years old in 2019 (Figures 3A,B). The most significant sex difference occurred in South Asia (Figures 2A,B). Some other notable patterns were observed in low-middle and low-SDI regions. During the past three decades, the trends of deaths and DALYs attributable to high SBP declined across all age groups (Figures 4A,B).

**Figure 5:**
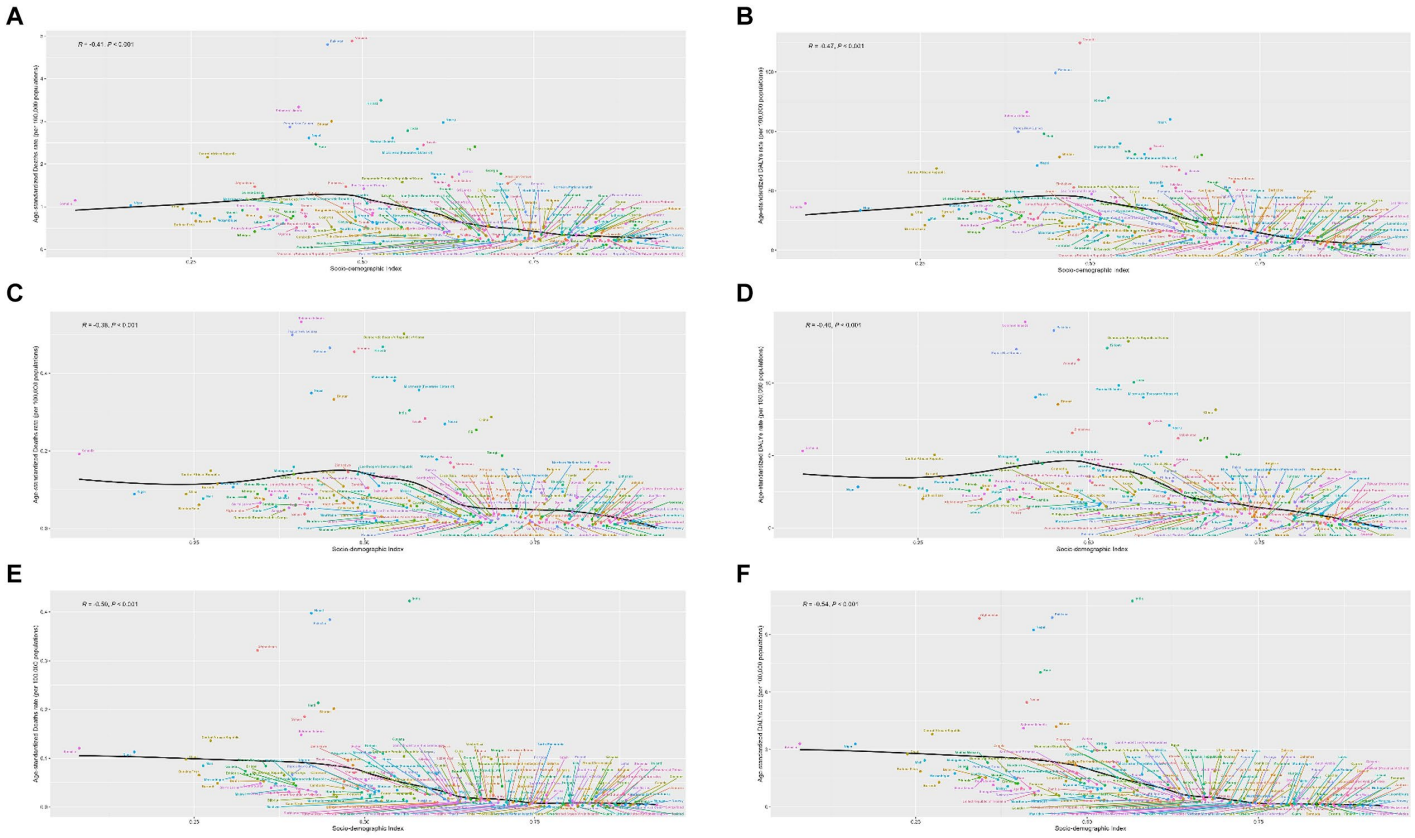
National age-standardized rates of mortality and disability-adjusted life year (DALY) of rheumatic heart disease risk due to available risk factors by socio-demographic index, 1990–2019. **(A)** Mortality due to high systolic blood pressure; **(B)** DALY due to high systolic blood pressure; **(C)** Mortality due to diet high in sodium; **(D)** DALY due to diet high in sodium; **(E)** mortality due to lead exposure; and **(F)** DALY due to lead exposure.

Both age-standardized rates of death and DALYs of RHD due to high SBP showed a moderate negative correlation with the SDI in 2019 (correlation coefficients were approximately −0.47 and − 0.41, respectively) at the national level (Figures 5A,B).

### Burden of RHD attributable to a diet high in sodium

In 2019, globally, a total number of 11.9 (95%UI: 2.8 to 29.1) deaths and 390.8 (95%UI: 98.1 to 930.5) DALYs were caused by RHD due to a diet high in sodium, decreasing by 38 and 37% from 1990, respectively (Table 1). The countries with the highest absolute burden were China deaths: 5.5 (1.9 to 11.5) and DALYs: 163.9 (68.5 to 304.4; Supplementary Tables S3, S4). India, Pakistan, Brazil, the Democratic People’s Republic of Korea, and Bangladesh were all on the list of the most burdened countries. The global age-standardized rates of deaths and DALYs were 0.1 (0 to 0.4, per 100,000) and 4.7 (1.2 to 11.2, per 100,000), respectively, with an AAPC of −3.99 (−4.14 to −3.85), and − 3.74 (−3.89 to −3.6) (Table 1). In general, countries in Oceania, East Asia, and South Asia had higher rates of deaths and DALYs (Table 1). Pakistan had the highest recorded death rate (0.5 per 100,000), and Solomon Islands had the highest DALY rate (14.2 per 100,000) (Supplementary Tables S3, S4). In terms of the temporal changes, high sodium intake attributable to RHD deaths and DALYs decreased annually across the world from 1990 to 2019, except for Belgium and Pakistan (Supplementary Tables S3, S4).

All SDI quintiles exhibited a downward trend in the absolute RHD burden due to high sodium intake during the study period (Table 1). Of which, The middle-SDI quintile led the largest reduction in RHD burden due to high sodium intake, with an AAPC of −5.21 (−5.5 to −4.92; Table 1) in deaths and an AAPC of −5.05 (−5.24 to −4.86; Table 2) in DALYs. Meanwhile, the rates of deaths and DALYs were higher in the low-middle SDI quintile in 2019 (Table 1).

Additionally, males had a higher burden of RHD than females worldwide (Figures 2C,D). A massive sex disparity was observed in Oceania, Central Asia, and East Asia, where death and DALY rates of men were almost three times those of women (Figures 2C,D). However, males had a lower burden of RHD than females in Central Sub-Saharan Africa, and Eastern Sub-Saharan Africa. In 2019, the age-standardized rates of deaths and DALYs from RHD related to high sodium intake increased with age (Figures 3C,D). Moreover, high sodium intake-related deaths and DALYs declined in each age group during the past 30 years, with the highest decrease noted in the 45–49 years age group (Figures 4A,B).

Both age-standardized rates of death and DALYs of RHD due to a diet high in sodium showed a strong negative correlation with the SDI in 2019 (correlation coefficients were approximately −0.46 and − 0.38, respectively) at the national level (Figures 5C,D).

### Burden of RHD attributable to lead exposure

In 2019, globally, there were 7.7 (95% UI: 4.1 to 13.5) RHD deaths attributable to lead exposure, with a rate of 0.1 per 100,000 (95% UI: 0 to 0.2) (Table 1). Additionally, DALYs attributable to lead exposure were 213.5 (95% UI: 106.8 to 372.7), corresponding to a rate of 2.6 per 100,000 (95% UI: 1.3 to 4.6) (Table 1). South Asia had the highest death and DALY rates in 2019. All 21 GBD regions showed downward trends of disease burden attributable to lead exposure from 1990 to 2019 (Figure 1). The three countries with the most deaths and DALYs attributable to lead exposure were India, China, and Pakistan (Supplementary Tables S5, S6). India, Pakistan, and Nepal were the countries with the highest rates of death (Supplementary Table S5), and India, Pakistan, and Afghanistan were those with the highest rates of DALYs (Supplementary Table S6). Most countries showed decreasing trends of disease burden due to lead exposure. Thailand and Singapore had the largest decreases in disease burden, while the highest increase was observed in Georgia (Supplementary Table S6).

At the SDI region level, the low-middle SDI region had the highest number of deaths and DALYs, but the highest rates of death and DALYs occurred in the low-SDI region, respectively. Of the 21 GBD regions, the highest number and rates of deaths and DALYs were in South Asia (Table 1). All SDI quintiles showed a downward trend of RHD deaths and DALYs attributable to lead exposure, where the high-middle SDI quintile had the largest changes (Figure 1).

The sex-specific death and DALY rates in locations were varied (Figures 2E,F). In most regions, males had a much higher burden than females, while this disparity reversed in South Asia. The global number of deaths in 2019 showed an unimodal distribution with age (Figures 3E,F). Age-specific mortality and DALY rates in males were higher than those in females before the age of 35–39, after which the trend reversed. Moreover, lead exposure-related deaths and DALYs declined in each age group except for the 90+ years age group during the past 30 years, with the highest decrease noted in the 25–29 years age group (Figures 4A,B).

Both age-standardized rates of death and DALYs of RHD due to lead exposure showed a moderate negative correlation with the SDI in 2019 (correlation coefficients were approximately −0.44 and − 0.41, respectively) at the national level (Figures 5E,F).

## Discussion

RHD is a significant global health concern, which caused approximately 0.31 million deaths worldwide in 2019 (9). In addition, the prevalence of RHD increased from 23.76 million in 1990 to 40.50 million in 2019, leading to predictions that cases of RHD could exceed 48 million by 2030 (10). Therefore, the disease represents an important global health issue requiring targeted prevention and control efforts. Herein, we conducted a comprehensive estimation of the burden of disease attributable to specific risk factors. Generally, we found that there appears to be a trend of decrease in global RHD-related numbers and rates of deaths and DALYs due to a diet high in sodium, high SBP, and lead exposure since 1990. The burden of RHD varied with different risk factors at regional and national levels, among which RHD disparities had different gradients of risk factors during this period. After that, tracking the disease burden caused by these risk factors helps formulate effective control measures to prevent RH.

Although the past 30 years have witnessed a decrease in high SBP-related RHD burden, high SBP still dominates among the risk-attributable factors in 2019. Hypertension May serve as a major risk factor for valvular heart disease, a known moderate risk factor for heart failure that is often associated with RHD (11). Higher SBP has been associated with an increase in the prevalence of CVDs. RHD, in particular, has been linked to an increased risk of fatal and non-fatal coronary heart disease and stroke (12). The burden of CVD attributable to hypertension is vast, which includes the impact on RHD patients given the role of high blood pressure in heart disease and stroke (13). Over the past few decades (1990–2019), the global incidence of RHD has increased, particularly impacting poverty-stricken and marginalized populations. The correlation with elevated SBP adds to the overall disease burden (14). However, while high SBP is not a direct cause of RHD, it May escalate the severity or prognosis of RHD due to its impact on cardiovascular health. It is also worth mentioning that these sources offer different perspectives on the subject and should be read fully for a comprehensive understanding.

Evidence demonstrates that sodium intake shows a positive correlation with blood pressure, which could increase the risk for CVDs, including RHD (15). Reducing salt intake could therefore form a key strategy to manage cardiovascular risks, including those related to RHD. As is always recommended, maintaining a balanced diet and limiting sodium intake can form part of a comprehensive strategy for overall cardiovascular health.

The escalating levels of lead exposure and its detrimental impact on human health emerged as a major public health concern during the 1990s. Lead exposure is known to contribute to the overall global burden of CVDs (16, 17). The overall estimations of GBD 2019 demonstrated that lead exposure accounted for 8.2, 7.2, and 5.65% of the global burden of hypertensive heart disease, ischemic heart disease, and stroke, respectively (16). In our study, we found that the DALYs attributable to lead exposure were 213,500, and its rate was 2.6 per 100,000, with the highest rate observed in developing countries.

Although rheumatic fever often affects children and adolescents (2), in 2019, the DALY rates of RHD attributable to the above risk exposures were positively associated with increasing age in both males and females, which indicates that the disease continues in youth into middle-aged and older adults. With the development of modern sophisticated biological valves and mechanical valves, the survival rate of patients with rheumatic heart valve disease is increasingly improving. However, children and adolescents are particularly vulnerable to long-term harm from lead, including increased risks of high blood pressure and heart disease (18). In addition, age-standardized mortality and DALYs from RHD attributable to lead exposure and hypertension were higher in men than in women before the age of 39, while the risk attributable to a high sodium diet was higher in women until the age of 80. This May be related to physical differences and lifestyle differences between men and women (19). Specifically, women often exhibit different patterns of risk factor exposure, disease progression, and health outcomes compared to men. Women with RHD May have different clinical presentations and May be more susceptible to the adverse effects of conditions such as hypertension and dietary sodium intake, potentially due to physiological and hormonal factors. Additionally, gender differences in healthcare access and treatment-seeking behavior May further exacerbate these disparities, contributing to the observed patterns in RHD burden.

Since RHD accounts for a large proportion of DALYs (9) and imposes a relatively high burden (10), particularly in the low-middle and low-SDI countries, the preventive strategies against related risk factors, such as a diet high in sodium, high SBP, and lead exposure, seem to be cost-effective in reducing the disease burden. Measurements for behavioral risk factors such as adopting healthy diets, metabolic risk factors such as blood pressure control, as well as environmental risk factors such as reducing lead exposure, should also be emphasized.

The strengths of the current study include the comprehensive and up-to-date estimation of the situation and trends of the attributable burden to diet high in sodium, high SBP, and lead exposure and its attributable RHD, based on the data from GBD 2019 during 1990–2019. However, there were some major limitations of the present study. First, it resulted from GBD methodologies in data collection, thus, the availability and quality of primary data in regions with countries that have poor completeness rates of the data sources. In the absence of data, estimates rely heavily on the predictive validity of the modeling efforts applied to out-of-sample scenarios. Additionally, our data did not take into account the interplay among risk factors, which could possibly affect the reliability of the results. Third, we did not analyze the RHD burden attributable to other risk factors, including infectious agents, physical activity, and comorbidities because there are no data in the GBD database. This May overestimate the burden of RHD attributable to the included risk factors in this study. Fourth, the GBD study relies on observational data and statistical modeling to estimate disease burden and associated risk factors across time periods rather than following a specific cohort of individuals longitudinally. Moreover, there are potential biases due to the heterogeneity of the studies included in the GBD database. These studies vary in design, population characteristics, and data collection methods, which could introduce bias in the estimation of risk factors. Although the GBD study uses sophisticated modeling techniques to mitigate these biases, some residual bias May still exist and should be considered when interpreting the results. Finally, comprehensive and meticulous study is required to enhance the proof that supports understanding the mediating impacts of those factors on the burden of risk attributable to RHD.

## Conclusion

Age-standardized global DALY and death rates of RHD attributed to a diet high in sodium, high SBP, and lead exposure decreased from 1990 to 2019, and both are reversely associated with SDI. Furthermore, we found that the major attributable age-standardized DALYs were from hypertension. Considering the substantial burden of RHD in certain regions, as well as the detrimental health impacts of associated risk factors, there is an immediate need for strategic attention and measures to regulate these factors and their consequent burden. Furthermore, the absence of dependable exposure information from every country worldwide emphasizes the demand for additional research to precisely ascertain the disease burden linked to these factors.

## Data Availability

The data that support the findings of this study are available on request from the corresponding author, JJ, upon reasonable request.
