# Supplementary material for: Spatiotemporal patterns of rheumatic heart disease burden attributable to high systolic blood pressure, high sodium diet, and lead exposure (1990 to 2019): a longitudinal observational study
Source: Front Nutr. 2024 Sep 26;11:1419349. doi: 10.3389/fnut.2024.1419349 (PMC11466049; doi:10.3389/fnut.2024.1419349)
Supplement: Supplementary file 1 [file Table_1.docx]

**Supplementary table 1. Deaths of rheumatic heart disease due to high systolic blood pressure.**

| **Location** | **1990 Counts**  **(thousand)** | **Age-standardised rate (per 100 000 population), 1990** | **2019 Counts**  **(thousand)** | **Age-standardised rate (per 100 000 population), 2019** | **Average annual percent change** |
| --- | --- | --- | --- | --- | --- |
| Afghanistan | 0.2 (0.1 to 0.4) | 2.5 (1 to 5.7) | 0.2 (0.1 to 0.4) | 1.5 (0.7 to 2.9) | -1.81 (-1.9 to -1.73) |
| Albania | 0 (0 to 0.1) | 1.8 (1.2 to 2.8) | 0 (0 to 0) | 0.3 (0.2 to 0.5) | -5.85 (-6.44 to -5.26) |
| Algeria | 0.1 (0.1 to 0.2) | 1 (0.6 to 1.8) | 0.1 (0.1 to 0.2) | 0.3 (0.2 to 0.6) | -3.69 (-3.8 to -3.59) |
| American Samoa | 0 (0 to 0) | 2 (1.3 to 3.4) | 0 (0 to 0) | 1.5 (1 to 2.4) | -1.07 (-1.45 to -0.68) |
| Andorra | 0 (0 to 0) | 0.5 (0.3 to 0.9) | 0 (0 to 0) | 0.3 (0.1 to 0.5) | -2.14 (-2.35 to -1.93) |
| Angola | 0.1 (0 to 0.1) | 2 (1.1 to 3.5) | 0.1 (0.1 to 0.2) | 1 (0.6 to 1.6) | -2.4 (-2.59 to -2.21) |
| Antigua and Barbuda | 0 (0 to 0) | 0.5 (0.3 to 0.8) | 0 (0 to 0) | 0.2 (0.2 to 0.4) | -2.39 (-2.83 to -1.94) |
| Argentina | 0.3 (0.2 to 0.6) | 1.2 (0.7 to 2.2) | 0.4 (0.2 to 0.7) | 0.7 (0.4 to 1.3) | -1.98 (-2.31 to -1.65) |
| Armenia | 0.1 (0 to 0.1) | 2.3 (1.6 to 3.5) | 0 (0 to 0.1) | 0.8 (0.5 to 1.3) | -3.33 (-3.94 to -2.7) |
| Australia | 0.1 (0.1 to 0.2) | 0.7 (0.4 to 1.1) | 0.1 (0.1 to 0.2) | 0.2 (0.1 to 0.5) | -3.28 (-3.6 to -2.97) |
| Austria | 0.1 (0.1 to 0.2) | 0.8 (0.5 to 1.4) | 0.1 (0 to 0.2) | 0.4 (0.2 to 0.8) | -2.47 (-2.77 to -2.16) |
| Azerbaijan | 0.1 (0 to 0.1) | 1.2 (0.8 to 1.8) | 0 (0 to 0.1) | 0.5 (0.3 to 0.8) | -2.63 (-2.88 to -2.39) |
| Bahamas | 0 (0 to 0) | 0.4 (0.3 to 0.7) | 0 (0 to 0) | 0.2 (0.1 to 0.4) | -1.95 (-2.13 to -1.76) |
| Bahrain | 0 (0 to 0) | 0.6 (0.4 to 1.2) | 0 (0 to 0) | 0.3 (0.2 to 0.5) | -2.62 (-3.16 to -2.08) |
| Bangladesh | 0.4 (0.3 to 0.7) | 0.9 (0.5 to 1.4) | 0.8 (0.5 to 1.2) | 0.6 (0.4 to 1) | -0.81 (-1.55 to -0.08) |
| Barbados | 0 (0 to 0) | 0.5 (0.3 to 0.8) | 0 (0 to 0) | 0.3 (0.2 to 0.4) | -1.76 (-2.34 to -1.19) |
| Belarus | 0.3 (0.2 to 0.4) | 2.4 (1.6 to 3.4) | 0.1 (0.1 to 0.2) | 0.6 (0.4 to 1) | -4.48 (-5.22 to -3.73) |
| Belgium | 0 (0 to 0.1) | 0.3 (0.1 to 0.5) | 0.1 (0.1 to 0.3) | 0.5 (0.2 to 0.9) | 1.8 (1.45 to 2.15) |
| Belize | 0 (0 to 0) | 0.4 (0.2 to 0.6) | 0 (0 to 0) | 0.2 (0.2 to 0.4) | -1.56 (-1.83 to -1.3) |
| Benin | 0 (0 to 0.1) | 1.6 (0.9 to 2.7) | 0 (0 to 0.1) | 0.8 (0.5 to 1.2) | -2.55 (-2.74 to -2.37) |
| Bermuda | 0 (0 to 0) | 0.4 (0.2 to 0.6) | 0 (0 to 0) | 0.1 (0.1 to 0.2) | -4.34 (-4.5 to -4.18) |
| Bhutan | 0 (0 to 0) | 5.4 (2.6 to 10.4) | 0 (0 to 0) | 3 (1.4 to 6.4) | -2.03 (-2.14 to -1.93) |
| Bolivia (Plurinational State of) | 0 (0 to 0.1) | 1.1 (0.6 to 2.4) | 0 (0 to 0.1) | 0.5 (0.3 to 0.9) | -2.77 (-2.99 to -2.56) |
| Bosnia and Herzegovina | 0 (0 to 0.1) | 0.8 (0.5 to 1.3) | 0 (0 to 0) | 0.2 (0.1 to 0.3) | -5.16 (-5.53 to -4.8) |
| Botswana | 0 (0 to 0) | 1.6 (0.9 to 2.7) | 0 (0 to 0) | 0.7 (0.3 to 1.1) | -3.05 (-3.18 to -2.92) |
| Brazil | 0.7 (0.5 to 1) | 0.7 (0.5 to 1.1) | 0.7 (0.5 to 1.1) | 0.3 (0.2 to 0.5) | -2.76 (-2.96 to -2.57) |
| Brunei Darussalam | 0 (0 to 0) | 1.3 (0.8 to 2.1) | 0 (0 to 0) | 0.6 (0.4 to 1.1) | -2.49 (-2.63 to -2.35) |
| Bulgaria | 0.3 (0.2 to 0.4) | 2.1 (1.4 to 3.1) | 0.1 (0 to 0.1) | 0.5 (0.3 to 0.8) | -4.8 (-5.19 to -4.4) |
| Burkina Faso | 0 (0 to 0.1) | 0.9 (0.5 to 1.7) | 0.1 (0 to 0.1) | 0.7 (0.4 to 1.1) | -0.91 (-1.16 to -0.65) |
| Burundi | 0 (0 to 0) | 1.2 (0.6 to 2) | 0 (0 to 0.1) | 0.7 (0.4 to 1.3) | -1.66 (-1.73 to -1.58) |
| Cabo Verde | 0 (0 to 0) | 1.9 (1.2 to 2.8) | 0 (0 to 0) | 0.4 (0.3 to 0.6) | -5.25 (-5.94 to -4.55) |
| Cambodia | 0.1 (0 to 0.1) | 1.5 (0.9 to 2.3) | 0.1 (0 to 0.1) | 0.4 (0.2 to 0.7) | -4.23 (-4.38 to -4.08) |
| Cameroon | 0 (0 to 0.1) | 1.2 (0.7 to 2.3) | 0.1 (0 to 0.1) | 0.7 (0.4 to 1.2) | -1.88 (-1.97 to -1.8) |
| Canada | 0.2 (0.1 to 0.3) | 0.5 (0.3 to 0.9) | 0.2 (0.1 to 0.3) | 0.2 (0.1 to 0.4) | -2.84 (-3.09 to -2.59) |
| Central African Republic | 0 (0 to 0.1) | 2.9 (1.5 to 5.1) | 0 (0 to 0.1) | 2.2 (1 to 4.1) | -0.93 (-1.02 to -0.85) |
| Chad | 0 (0 to 0.1) | 1.7 (0.9 to 3.4) | 0.1 (0 to 0.1) | 1 (0.5 to 1.8) | -1.93 (-2.07 to -1.79) |
| Chile | 0.1 (0.1 to 0.2) | 1.3 (0.9 to 1.9) | 0.1 (0 to 0.1) | 0.2 (0.1 to 0.4) | -5.63 (-6.12 to -5.13) |
| China | 27.1 (16.4 to 46.1) | 3.7 (2.2 to 6.8) | 18.2 (11.3 to 29.3) | 1 (0.6 to 1.7) | -4.45 (-4.74 to -4.17) |
| Colombia | 0.1 (0.1 to 0.1) | 0.4 (0.3 to 0.7) | 0 (0 to 0.1) | 0.1 (0.1 to 0.1) | -5.78 (-6.21 to -5.35) |
| Comoros | 0 (0 to 0) | 0.9 (0.4 to 1.6) | 0 (0 to 0) | 0.5 (0.3 to 0.9) | -1.99 (-2.38 to -1.59) |
| Congo | 0 (0 to 0) | 2.1 (1.2 to 3.3) | 0 (0 to 0) | 0.9 (0.5 to 1.5) | -2.93 (-3.24 to -2.61) |
| Cook Islands | 0 (0 to 0) | 0.9 (0.5 to 1.3) | 0 (0 to 0) | 0.5 (0.3 to 0.8) | -1.67 (-2.1 to -1.25) |
| Costa Rica | 0 (0 to 0) | 0.8 (0.5 to 1.1) | 0 (0 to 0) | 0.3 (0.2 to 0.5) | -2.73 (-3.34 to -2.12) |
| Croatia | 0.1 (0.1 to 0.1) | 1.4 (0.9 to 2.2) | 0 (0 to 0.1) | 0.3 (0.2 to 0.6) | -4.27 (-5.8 to -2.72) |
| Cuba | 0.1 (0 to 0.1) | 0.5 (0.3 to 0.8) | 0 (0 to 0.1) | 0.2 (0.1 to 0.3) | -2.62 (-3.05 to -2.18) |
| Cyprus | 0 (0 to 0) | 2 (1.1 to 4.1) | 0 (0 to 0) | 0.7 (0.4 to 1.3) | -3.49 (-3.94 to -3.04) |
| Czechia | 0.2 (0.2 to 0.4) | 1.8 (1.2 to 2.7) | 0.1 (0 to 0.1) | 0.3 (0.2 to 0.6) | -5.66 (-6.02 to -5.3) |
| Côte d'Ivoire | 0.1 (0 to 0.1) | 1.5 (0.9 to 2.5) | 0.1 (0 to 0.1) | 0.7 (0.4 to 1.2) | -2.65 (-2.83 to -2.47) |
| Democratic People's Republic of Korea | 0.5 (0.3 to 0.8) | 3.4 (2 to 5.3) | 0.5 (0.2 to 0.9) | 1.6 (0.8 to 3.2) | -2.52 (-2.73 to -2.3) |
| Democratic Republic of the Congo | 0.3 (0.2 to 0.5) | 1.9 (1.1 to 3.3) | 0.4 (0.2 to 0.8) | 1.1 (0.5 to 2.2) | -1.9 (-2.07 to -1.73) |
| Denmark | 0.1 (0.1 to 0.1) | 1 (0.6 to 1.7) | 0 (0 to 0) | 0.2 (0.1 to 0.4) | -5.74 (-6.82 to -4.65) |
| Djibouti | 0 (0 to 0) | 0.7 (0.4 to 1.2) | 0 (0 to 0) | 0.5 (0.3 to 0.8) | -1.57 (-1.67 to -1.46) |
| Dominica | 0 (0 to 0) | 0.8 (0.5 to 1.3) | 0 (0 to 0) | 0.4 (0.3 to 0.8) | -2.32 (-2.45 to -2.19) |
| Dominican Republic | 0 (0 to 0) | 0.5 (0.3 to 0.8) | 0 (0 to 0.1) | 0.3 (0.2 to 0.5) | -1.47 (-2.18 to -0.76) |
| Ecuador | 0 (0 to 0) | 0.5 (0.3 to 1) | 0 (0 to 0.1) | 0.2 (0.1 to 0.4) | -3.03 (-3.34 to -2.72) |
| Egypt | 0.3 (0.2 to 0.6) | 1 (0.5 to 2.1) | 0.3 (0.2 to 0.6) | 0.5 (0.3 to 0.9) | -2.28 (-2.64 to -1.93) |
| El Salvador | 0 (0 to 0) | 0.4 (0.2 to 0.6) | 0 (0 to 0) | 0.2 (0.1 to 0.3) | -2.73 (-3.22 to -2.24) |
| Equatorial Guinea | 0 (0 to 0) | 2.3 (1.1 to 4.8) | 0 (0 to 0) | 0.6 (0.3 to 1) | -4.78 (-4.97 to -4.59) |
| Eritrea | 0 (0 to 0) | 1.2 (0.6 to 2.4) | 0 (0 to 0) | 0.7 (0.4 to 1.2) | -1.55 (-1.65 to -1.44) |
| Estonia | 0 (0 to 0.1) | 1.8 (1.2 to 2.6) | 0 (0 to 0) | 0.3 (0.2 to 0.5) | -5.82 (-6.41 to -5.24) |
| Eswatini | 0 (0 to 0) | 1.5 (0.8 to 2.3) | 0 (0 to 0) | 0.8 (0.5 to 1.4) | -1.99 (-2.3 to -1.68) |
| Ethiopia | 0.1 (0.1 to 0.2) | 0.7 (0.4 to 1.2) | 0.2 (0.1 to 0.3) | 0.4 (0.2 to 0.7) | -1.61 (-1.67 to -1.54) |
| Fiji | 0 (0 to 0) | 3.2 (2 to 4.9) | 0 (0 to 0) | 2.4 (1.6 to 3.8) | -0.95 (-1.33 to -0.57) |
| Finland | 0.1 (0 to 0.1) | 0.7 (0.5 to 1.2) | 0 (0 to 0) | 0.1 (0.1 to 0.2) | -6.27 (-6.53 to -6) |
| France | 0.6 (0.4 to 1.1) | 0.7 (0.4 to 1.2) | 0.7 (0.3 to 1.3) | 0.4 (0.2 to 0.7) | -2.11 (-2.4 to -1.82) |
| Gabon | 0 (0 to 0) | 1.2 (0.7 to 2.1) | 0 (0 to 0) | 0.5 (0.3 to 0.9) | -2.89 (-3.03 to -2.74) |
| Gambia | 0 (0 to 0) | 1.4 (0.8 to 2.3) | 0 (0 to 0) | 0.8 (0.5 to 1.2) | -1.78 (-2.5 to -1.05) |
| Georgia | 0.1 (0.1 to 0.1) | 1.4 (1 to 2.1) | 0.1 (0.1 to 0.2) | 1.8 (1.1 to 2.9) | 0.86 (0.38 to 1.34) |
| Germany | 1.5 (1 to 2.6) | 1.2 (0.8 to 2) | 1.3 (0.7 to 2.6) | 0.6 (0.3 to 1.1) | -2.51 (-2.92 to -2.1) |
| Ghana | 0.1 (0 to 0.1) | 1 (0.6 to 1.8) | 0.1 (0.1 to 0.1) | 0.5 (0.3 to 0.9) | -2.28 (-2.41 to -2.14) |
| Greece | 0.1 (0 to 0.1) | 0.5 (0.3 to 0.8) | 0 (0 to 0.1) | 0.1 (0.1 to 0.2) | -3.96 (-4.48 to -3.44) |
| Greenland | 0 (0 to 0) | 1.3 (0.7 to 2.5) | 0 (0 to 0) | 0.3 (0.2 to 0.7) | -4.64 (-5.37 to -3.91) |
| Grenada | 0 (0 to 0) | 1.4 (0.9 to 2.2) | 0 (0 to 0) | 0.6 (0.4 to 0.9) | -3.02 (-3.32 to -2.72) |
| Guam | 0 (0 to 0) | 1 (0.6 to 1.7) | 0 (0 to 0) | 0.6 (0.4 to 1) | -1.82 (-2.31 to -1.33) |
| Guatemala | 0 (0 to 0) | 0.2 (0.1 to 0.4) | 0 (0 to 0) | 0.1 (0.1 to 0.2) | -1.9 (-3.09 to -0.7) |
| Guinea | 0.1 (0 to 0.1) | 1.7 (1 to 3.3) | 0.1 (0 to 0.1) | 0.9 (0.6 to 1.5) | -2.19 (-2.34 to -2.05) |
| Guinea-Bissau | 0 (0 to 0) | 2.8 (1.5 to 4.5) | 0 (0 to 0) | 1.3 (0.7 to 2) | -2.71 (-2.82 to -2.6) |
| Guyana | 0 (0 to 0) | 1 (0.6 to 1.5) | 0 (0 to 0) | 0.5 (0.3 to 0.8) | -2.24 (-2.58 to -1.89) |
| Haiti | 0.2 (0.1 to 0.3) | 4.4 (2.3 to 8.6) | 0.2 (0.1 to 0.4) | 2.5 (1.2 to 4.6) | -1.97 (-2.06 to -1.88) |
| Honduras | 0 (0 to 0) | 0.3 (0.2 to 0.5) | 0 (0 to 0) | 0.2 (0.1 to 0.4) | -0.77 (-1.52 to -0.02) |
| Hungary | 0.4 (0.3 to 0.5) | 2.6 (1.8 to 3.8) | 0.1 (0 to 0.1) | 0.3 (0.2 to 0.6) | -6.55 (-6.86 to -6.25) |
| Iceland | 0 (0 to 0) | 0.2 (0.1 to 0.4) | 0 (0 to 0) | 0.1 (0.1 to 0.2) | -2.38 (-2.66 to -2.1) |
| India | 23.9 (15 to 35.8) | 5.2 (3.3 to 8.4) | 31.2 (20 to 45.1) | 2.8 (1.8 to 4.2) | -2.19 (-2.72 to -1.66) |
| Indonesia | 0.7 (0.4 to 1) | 0.7 (0.4 to 1) | 0.4 (0.3 to 0.6) | 0.2 (0.1 to 0.3) | -3.69 (-3.84 to -3.54) |
| Iran (Islamic Republic of) | 0.2 (0.1 to 0.4) | 0.8 (0.5 to 1.6) | 0.2 (0.2 to 0.4) | 0.3 (0.2 to 0.6) | -2.75 (-3.12 to -2.37) |
| Iraq | 0.1 (0.1 to 0.2) | 1.6 (0.9 to 2.7) | 0.1 (0.1 to 0.2) | 0.5 (0.3 to 0.8) | -3.94 (-4.37 to -3.51) |
| Ireland | 0 (0 to 0) | 0.7 (0.5 to 1.1) | 0 (0 to 0) | 0.2 (0.1 to 0.4) | -3.93 (-4.41 to -3.44) |
| Israel | 0 (0 to 0.1) | 0.8 (0.5 to 1.4) | 0.1 (0 to 0.1) | 0.5 (0.3 to 0.8) | -1.76 (-1.97 to -1.56) |
| Italy | 0.9 (0.6 to 1.4) | 1.1 (0.7 to 1.6) | 0.6 (0.3 to 1.2) | 0.4 (0.2 to 0.7) | -3.51 (-4.02 to -3.01) |
| Jamaica | 0 (0 to 0) | 0.4 (0.3 to 0.7) | 0 (0 to 0) | 0.2 (0.1 to 0.4) | -1.91 (-2.3 to -1.52) |
| Japan | 1 (0.6 to 1.6) | 0.6 (0.4 to 1.1) | 1.1 (0.5 to 2.3) | 0.2 (0.1 to 0.4) | -3.76 (-4.04 to -3.48) |
| Jordan | 0 (0 to 0) | 0.4 (0.2 to 0.7) | 0 (0 to 0) | 0.1 (0.1 to 0.2) | -4.11 (-4.55 to -3.68) |
| Kazakhstan | 0.4 (0.3 to 0.6) | 2.9 (2.1 to 4.2) | 0.1 (0.1 to 0.2) | 0.7 (0.5 to 1.1) | -4.75 (-5.14 to -4.36) |
| Kenya | 0.1 (0 to 0.1) | 0.7 (0.3 to 1.1) | 0.1 (0 to 0.2) | 0.5 (0.2 to 0.9) | -1.08 (-1.16 to -1) |
| Kiribati | 0 (0 to 0) | 5.1 (3.1 to 8.1) | 0 (0 to 0) | 3.5 (2 to 5.7) | -1.28 (-1.34 to -1.21) |
| Kuwait | 0 (0 to 0) | 0.5 (0.3 to 0.8) | 0 (0 to 0) | 0.1 (0.1 to 0.2) | -4.91 (-5.9 to -3.91) |
| Kyrgyzstan | 0.1 (0 to 0.1) | 2.2 (1.4 to 3.2) | 0 (0 to 0.1) | 0.6 (0.4 to 1) | -4.46 (-5.42 to -3.5) |
| Lao People's Democratic Republic | 0 (0 to 0.1) | 1.9 (1 to 3.2) | 0 (0 to 0.1) | 0.8 (0.4 to 1.2) | -3.06 (-3.14 to -2.97) |
| Latvia | 0.1 (0.1 to 0.1) | 2.4 (1.6 to 3.5) | 0 (0 to 0) | 0.4 (0.3 to 0.6) | -5.81 (-7.54 to -4.04) |
| Lebanon | 0 (0 to 0) | 0.6 (0.4 to 1.2) | 0 (0 to 0) | 0.2 (0.1 to 0.4) | -3.47 (-3.61 to -3.33) |
| Lesotho | 0 (0 to 0) | 1.4 (0.7 to 2.5) | 0 (0 to 0) | 1.1 (0.6 to 1.8) | -0.92 (-1.05 to -0.79) |
| Liberia | 0 (0 to 0) | 1.5 (0.9 to 2.7) | 0 (0 to 0) | 0.7 (0.4 to 1.3) | -2.63 (-2.83 to -2.43) |
| Libya | 0 (0 to 0) | 0.6 (0.3 to 1.2) | 0 (0 to 0) | 0.3 (0.2 to 0.5) | -2.11 (-2.44 to -1.79) |
| Lithuania | 0.1 (0.1 to 0.2) | 2.8 (1.9 to 3.9) | 0 (0 to 0) | 0.5 (0.3 to 0.8) | -5.9 (-6.63 to -5.17) |
| Luxembourg | 0 (0 to 0) | 0.6 (0.4 to 1.1) | 0 (0 to 0) | 0.3 (0.2 to 0.5) | -2.67 (-2.87 to -2.47) |
| Madagascar | 0.1 (0 to 0.1) | 1.3 (0.8 to 2.1) | 0.1 (0.1 to 0.2) | 1.1 (0.6 to 1.7) | -0.59 (-0.91 to -0.27) |
| Malawi | 0 (0 to 0.1) | 1 (0.6 to 1.6) | 0 (0 to 0.1) | 0.6 (0.4 to 1) | -1.58 (-1.65 to -1.5) |
| Malaysia | 0.1 (0.1 to 0.2) | 1.2 (0.8 to 1.7) | 0.1 (0 to 0.1) | 0.3 (0.2 to 0.4) | -4.78 (-5.18 to -4.38) |
| Maldives | 0 (0 to 0) | 1.4 (0.8 to 2.4) | 0 (0 to 0) | 0.3 (0.2 to 0.5) | -5.07 (-5.28 to -4.87) |
| Mali | 0.1 (0 to 0.1) | 1.9 (1.1 to 3.3) | 0.1 (0 to 0.1) | 0.8 (0.5 to 1.3) | -2.91 (-3.08 to -2.74) |
| Malta | 0 (0 to 0) | 0.7 (0.4 to 1.2) | 0 (0 to 0) | 0.3 (0.2 to 0.5) | -2.98 (-3.21 to -2.76) |
| Marshall Islands | 0 (0 to 0) | 3.5 (1.9 to 6.4) | 0 (0 to 0) | 2.6 (1.4 to 4.6) | -1.04 (-1.23 to -0.85) |
| Mauritania | 0 (0 to 0) | 1.5 (0.9 to 2.7) | 0 (0 to 0) | 0.5 (0.3 to 0.8) | -4.13 (-4.35 to -3.92) |
| Mauritius | 0 (0 to 0) | 0.8 (0.6 to 1.3) | 0 (0 to 0) | 0.2 (0.1 to 0.3) | -4.82 (-5.45 to -4.17) |
| Mexico | 0.4 (0.3 to 0.6) | 0.9 (0.6 to 1.3) | 0.3 (0.2 to 0.4) | 0.2 (0.2 to 0.4) | -4.54 (-4.72 to -4.36) |
| Micronesia (Federated States of) | 0 (0 to 0) | 3.8 (2.1 to 6.8) | 0 (0 to 0) | 2.4 (1.1 to 4.2) | -1.67 (-1.74 to -1.61) |
| Monaco | 0 (0 to 0) | 0.3 (0.2 to 0.4) | 0 (0 to 0) | 0.2 (0.1 to 0.3) | -1.67 (-1.74 to -1.61) |
| Mongolia | 0 (0 to 0.1) | 4.2 (2.4 to 7.6) | 0 (0 to 0.1) | 1.7 (1.1 to 2.7) | -3.06 (-3.25 to -2.88) |
| Montenegro | 0 (0 to 0) | 0.7 (0.4 to 1.1) | 0 (0 to 0) | 0.4 (0.3 to 0.6) | -1.69 (-1.96 to -1.42) |
| Morocco | 0.2 (0.1 to 0.4) | 1.6 (0.8 to 3.5) | 0.2 (0.1 to 0.3) | 0.7 (0.4 to 1.2) | -2.78 (-2.97 to -2.59) |
| Mozambique | 0.1 (0 to 0.1) | 1 (0.6 to 1.8) | 0.1 (0.1 to 0.1) | 0.8 (0.5 to 1.2) | -0.93 (-1.01 to -0.84) |
| Myanmar | 0.4 (0.2 to 0.6) | 1.4 (0.9 to 2.5) | 0.3 (0.2 to 0.4) | 0.6 (0.4 to 0.9) | -2.94 (-3.02 to -2.85) |
| Namibia | 0 (0 to 0) | 1.6 (0.9 to 2.7) | 0 (0 to 0) | 0.6 (0.4 to 1) | -3.29 (-3.43 to -3.15) |
| Nauru | 0 (0 to 0) | 3.6 (2 to 5.8) | 0 (0 to 0) | 3 (1.8 to 4.3) | -0.65 (-0.82 to -0.47) |
| Nepal | 0.5 (0.3 to 0.9) | 5.2 (2.7 to 9.7) | 0.6 (0.3 to 1) | 2.6 (1.3 to 4.6) | -2.39 (-2.51 to -2.28) |
| Netherlands | 0.1 (0 to 0.1) | 0.3 (0.2 to 0.5) | 0.1 (0 to 0.2) | 0.2 (0.1 to 0.4) | -0.84 (-1.52 to -0.15) |
| New Zealand | 0 (0 to 0.1) | 1.2 (0.8 to 1.8) | 0 (0 to 0.1) | 0.5 (0.3 to 0.7) | -3.05 (-3.63 to -2.46) |
| Nicaragua | 0 (0 to 0) | 0.4 (0.3 to 0.7) | 0 (0 to 0) | 0.2 (0.1 to 0.3) | -3.43 (-3.93 to -2.93) |
| Niger | 0.1 (0 to 0.1) | 2 (1.1 to 4.3) | 0.1 (0 to 0.1) | 1 (0.5 to 2) | -2.28 (-2.43 to -2.13) |
| Nigeria | 0.6 (0.3 to 1.2) | 1.4 (0.8 to 2.9) | 0.5 (0.3 to 1) | 0.6 (0.4 to 1.2) | -2.67 (-3.07 to -2.26) |
| Niue | 0 (0 to 0) | 2.1 (1.3 to 3.2) | 0 (0 to 0) | 1.2 (0.7 to 2) | -1.82 (-1.95 to -1.69) |
| North Macedonia | 0 (0 to 0) | 1.4 (0.9 to 2.3) | 0 (0 to 0) | 0.4 (0.2 to 0.6) | -4.33 (-4.58 to -4.08) |
| Northern Mariana Islands | 0 (0 to 0) | 0.9 (0.5 to 1.6) | 0 (0 to 0) | 0.7 (0.5 to 1.2) | -0.82 (-0.94 to -0.69) |
| Norway | 0 (0 to 0.1) | 0.6 (0.4 to 1.1) | 0 (0 to 0) | 0.2 (0.1 to 0.4) | -3.79 (-4.2 to -3.37) |
| Oman | 0 (0 to 0) | 0.4 (0.2 to 0.8) | 0 (0 to 0) | 0.2 (0.1 to 0.3) | -2.56 (-2.95 to -2.16) |
| Pakistan | 3.7 (2.2 to 5.8) | 6.2 (3.8 to 10.4) | 5.8 (3.8 to 8.5) | 4.8 (3.1 to 7.2) | -0.89 (-0.96 to -0.81) |
| Palau | 0 (0 to 0) | 1.1 (0.6 to 1.9) | 0 (0 to 0) | 0.9 (0.6 to 1.4) | -0.89 (-1.01 to -0.77) |
| Palestine | 0 (0 to 0) | 0.5 (0.3 to 0.8) | 0 (0 to 0) | 0.2 (0.1 to 0.3) | -3.63 (-3.81 to -3.46) |
| Panama | 0 (0 to 0) | 0.5 (0.3 to 0.8) | 0 (0 to 0) | 0.2 (0.1 to 0.3) | -3.9 (-4.64 to -3.16) |
| Papua New Guinea | 0.1 (0 to 0.1) | 2.7 (1.2 to 5.6) | 0.2 (0.1 to 0.3) | 2.9 (1.3 to 5.3) | 0.13 (0.03 to 0.22) |
| Paraguay | 0 (0 to 0) | 0.4 (0.3 to 0.6) | 0 (0 to 0) | 0.2 (0.1 to 0.4) | -1.81 (-2.37 to -1.24) |
| Peru | 0 (0 to 0.1) | 0.3 (0.2 to 0.5) | 0 (0 to 0.1) | 0.1 (0.1 to 0.2) | -1.99 (-2.67 to -1.31) |
| Philippines | 0.1 (0.1 to 0.2) | 0.4 (0.3 to 0.6) | 0.4 (0.2 to 0.6) | 0.4 (0.3 to 0.7) | 0.27 (-0.15 to 0.68) |
| Poland | 1.2 (0.8 to 1.8) | 2.7 (1.9 to 4) | 0.3 (0.2 to 0.4) | 0.4 (0.2 to 0.6) | -6.6 (-7.09 to -6.11) |
| Portugal | 0.1 (0.1 to 0.2) | 1 (0.7 to 1.5) | 0.1 (0 to 0.1) | 0.3 (0.2 to 0.5) | -4.45 (-4.85 to -4.04) |
| Puerto Rico | 0 (0 to 0) | 0.3 (0.2 to 0.6) | 0 (0 to 0) | 0.1 (0.1 to 0.2) | -3.47 (-4.15 to -2.79) |
| Qatar | 0 (0 to 0) | 0.8 (0.4 to 1.8) | 0 (0 to 0) | 0.2 (0.1 to 0.4) | -4.04 (-4.79 to -3.28) |
| Republic of Korea | 0.1 (0 to 0.1) | 0.3 (0.2 to 0.5) | 0.1 (0 to 0.1) | 0.1 (0 to 0.1) | -4.81 (-5.3 to -4.31) |
| Republic of Moldova | 0.1 (0.1 to 0.2) | 2.3 (1.5 to 3.3) | 0 (0 to 0) | 0.5 (0.3 to 0.8) | -5.12 (-6.61 to -3.61) |
| Romania | 0.6 (0.5 to 0.9) | 2.3 (1.6 to 3.3) | 0.1 (0.1 to 0.2) | 0.4 (0.3 to 0.6) | -5.92 (-6.32 to -5.52) |
| Russian Federation | 3.6 (2.5 to 5.2) | 1.9 (1.4 to 2.8) | 0.9 (0.6 to 1.4) | 0.4 (0.3 to 0.6) | -5.27 (-6.2 to -4.33) |
| Rwanda | 0 (0 to 0.1) | 1.5 (0.9 to 2.5) | 0 (0 to 0) | 0.5 (0.3 to 0.8) | -3.58 (-3.75 to -3.41) |
| Saint Kitts and Nevis | 0 (0 to 0) | 1 (0.6 to 1.6) | 0 (0 to 0) | 0.3 (0.1 to 0.4) | -4.49 (-4.93 to -4.04) |
| Saint Lucia | 0 (0 to 0) | 1.4 (0.9 to 2.1) | 0 (0 to 0) | 0.4 (0.3 to 0.7) | -3.62 (-4 to -3.24) |
| Saint Vincent and the Grenadines | 0 (0 to 0) | 0.8 (0.5 to 1.2) | 0 (0 to 0) | 0.4 (0.3 to 0.7) | -2.09 (-2.49 to -1.69) |
| Samoa | 0 (0 to 0) | 2.4 (1.3 to 4) | 0 (0 to 0) | 1.8 (1 to 2.9) | -1.02 (-1.13 to -0.9) |
| San Marino | 0 (0 to 0) | 0.9 (0.5 to 1.7) | 0 (0 to 0) | 0.6 (0.3 to 1.1) | -1.3 (-1.53 to -1.07) |
| Sao Tome and Principe | 0 (0 to 0) | 2.2 (1.4 to 3.4) | 0 (0 to 0) | 1.4 (0.8 to 2.4) | -1.41 (-1.53 to -1.29) |
| Saudi Arabia | 0.1 (0 to 0.1) | 0.7 (0.4 to 1.7) | 0.1 (0 to 0.1) | 0.2 (0.1 to 0.3) | -3.94 (-4.2 to -3.68) |
| Senegal | 0 (0 to 0.1) | 1.5 (0.9 to 2.5) | 0 (0 to 0.1) | 0.7 (0.4 to 1.1) | -2.64 (-2.9 to -2.39) |
| Serbia | 0.1 (0.1 to 0.2) | 0.9 (0.6 to 1.8) | 0 (0 to 0.1) | 0.3 (0.2 to 0.5) | -3.62 (-4 to -3.23) |
| Seychelles | 0 (0 to 0) | 1.4 (1 to 2) | 0 (0 to 0) | 0.2 (0.2 to 0.3) | -6.09 (-6.31 to -5.87) |
| Sierra Leone | 0 (0 to 0.1) | 2 (1.1 to 3.3) | 0 (0 to 0.1) | 0.9 (0.5 to 1.5) | -2.46 (-2.6 to -2.33) |
| Singapore | 0 (0 to 0) | 0.6 (0.4 to 0.9) | 0 (0 to 0) | 0.1 (0 to 0.1) | -7.46 (-7.81 to -7.11) |
| Slovakia | 0 (0 to 0.1) | 0.8 (0.5 to 1.1) | 0 (0 to 0) | 0.3 (0.2 to 0.5) | -2.98 (-3.26 to -2.7) |
| Slovenia | 0 (0 to 0.1) | 1.5 (0.9 to 2.5) | 0 (0 to 0.1) | 0.6 (0.3 to 1.1) | -3.04 (-3.62 to -2.46) |
| Solomon Islands | 0 (0 to 0) | 4.3 (1.9 to 7.7) | 0 (0 to 0) | 3.3 (1.6 to 5.9) | -0.9 (-1.08 to -0.73) |
| Somalia | 0 (0 to 0.1) | 1.4 (0.7 to 2.6) | 0.1 (0 to 0.2) | 1.2 (0.5 to 2.4) | -0.68 (-0.83 to -0.53) |
| South Africa | 0.3 (0.2 to 0.4) | 1 (0.7 to 1.4) | 0.2 (0.1 to 0.3) | 0.5 (0.3 to 0.7) | -2.92 (-3.77 to -2.05) |
| South Sudan | 0 (0 to 0) | 0.8 (0.4 to 1.5) | 0 (0 to 0) | 0.6 (0.3 to 1.2) | -1.11 (-1.2 to -1.01) |
| Spain | 0.8 (0.5 to 1.4) | 1.5 (1 to 2.5) | 0.6 (0.3 to 1.2) | 0.5 (0.3 to 1) | -3.55 (-3.75 to -3.36) |
| Sri Lanka | 0.1 (0.1 to 0.1) | 0.8 (0.5 to 1.2) | 0.1 (0 to 0.1) | 0.2 (0.1 to 0.3) | -4.19 (-4.8 to -3.58) |
| Sudan | 0.2 (0.1 to 0.4) | 1.9 (0.9 to 4.5) | 0.2 (0.1 to 0.3) | 0.9 (0.5 to 1.8) | -2.75 (-2.82 to -2.67) |
| Suriname | 0 (0 to 0) | 0.4 (0.3 to 0.6) | 0 (0 to 0) | 0.2 (0.1 to 0.3) | -2.7 (-3.52 to -1.88) |
| Sweden | 0.1 (0 to 0.2) | 0.5 (0.3 to 1) | 0.1 (0 to 0.1) | 0.2 (0.1 to 0.4) | -3.32 (-3.51 to -3.13) |
| Switzerland | 0.1 (0 to 0.2) | 0.7 (0.4 to 1.4) | 0 (0 to 0.1) | 0.1 (0.1 to 0.3) | -5.32 (-5.59 to -5.05) |
| Syrian Arab Republic | 0.1 (0.1 to 0.3) | 2.4 (1.4 to 4.5) | 0 (0 to 0.1) | 0.4 (0.2 to 0.7) | -6.01 (-6.4 to -5.62) |
| Taiwan (Province of China) | 0.2 (0.1 to 0.3) | 1.2 (0.8 to 2.1) | 0.1 (0 to 0.1) | 0.1 (0.1 to 0.3) | -7.27 (-7.62 to -6.93) |
| Tajikistan | 0.1 (0.1 to 0.1) | 2.5 (1.7 to 3.9) | 0.1 (0 to 0.1) | 1 (0.6 to 1.5) | -3.1 (-3.47 to -2.73) |
| Thailand | 0.4 (0.2 to 0.6) | 0.9 (0.6 to 1.7) | 0.1 (0.1 to 0.2) | 0.1 (0.1 to 0.2) | -7.65 (-8.17 to -7.14) |
| Timor-Leste | 0 (0 to 0) | 1.3 (0.7 to 2.5) | 0 (0 to 0) | 0.8 (0.4 to 1.4) | -1.68 (-2 to -1.36) |
| Togo | 0 (0 to 0) | 1.6 (1 to 2.5) | 0 (0 to 0) | 0.8 (0.4 to 1.3) | -2.53 (-2.69 to -2.38) |
| Tokelau | 0 (0 to 0) | 2.3 (1.3 to 4) | 0 (0 to 0) | 1.5 (0.9 to 2.4) | -1.51 (-1.58 to -1.43) |
| Tonga | 0 (0 to 0) | 1.1 (0.7 to 1.6) | 0 (0 to 0) | 0.8 (0.5 to 1.3) | -0.95 (-1.25 to -0.66) |
| Trinidad and Tobago | 0 (0 to 0) | 0.6 (0.4 to 0.9) | 0 (0 to 0) | 0.2 (0.1 to 0.4) | -3.09 (-3.58 to -2.59) |
| Tunisia | 0 (0 to 0.1) | 0.6 (0.4 to 1.1) | 0 (0 to 0.1) | 0.3 (0.1 to 0.4) | -2.86 (-2.96 to -2.76) |
| Turkey | NA | NA | NA | NA | NA |
| Turkmenistan | 0 (0 to 0.1) | 1.7 (1.2 to 2.5) | 0 (0 to 0.1) | 0.7 (0.4 to 1.1) | -2.9 (-4.18 to -1.6) |
| Tuvalu | 0 (0 to 0) | 3.5 (1.8 to 5.9) | 0 (0 to 0) | 2.5 (1.4 to 4.3) | -1.23 (-1.33 to -1.12) |
| Uganda | 0.1 (0 to 0.1) | 1 (0.6 to 1.6) | 0.1 (0 to 0.1) | 0.5 (0.3 to 0.8) | -2.31 (-2.44 to -2.19) |
| Ukraine | 0.6 (0.3 to 1) | 0.8 (0.5 to 1.4) | 0.3 (0.2 to 0.5) | 0.5 (0.3 to 0.7) | -1.63 (-2.77 to -0.48) |
| United Arab Emirates | 0 (0 to 0) | 3.1 (1.5 to 6.9) | 0 (0 to 0.1) | 0.9 (0.5 to 2) | -4.08 (-4.67 to -3.49) |
| United Kingdom | 1 (0.6 to 1.6) | 1.1 (0.7 to 1.7) | 0.3 (0.2 to 0.6) | 0.2 (0.1 to 0.4) | -5.34 (-5.6 to -5.08) |
| United Republic of Tanzania | 0.1 (0 to 0.1) | 0.8 (0.5 to 1.2) | 0.1 (0.1 to 0.2) | 0.5 (0.3 to 0.9) | -1.23 (-1.33 to -1.14) |
| United States of America | 2.2 (1.4 to 3.7) | 0.7 (0.4 to 1.1) | 1.3 (0.8 to 2.6) | 0.2 (0.1 to 0.4) | -3.75 (-3.92 to -3.57) |
| United States Virgin Islands | 0 (0 to 0) | 0.4 (0.2 to 0.6) | 0 (0 to 0) | 0.2 (0.1 to 0.3) | -1.93 (-2.08 to -1.78) |
| Uruguay | 0 (0 to 0) | 0.6 (0.4 to 0.9) | 0 (0 to 0) | 0.3 (0.2 to 0.5) | -1.96 (-2.43 to -1.5) |
| Uzbekistan | 0.3 (0.2 to 0.4) | 1.9 (1.3 to 2.9) | 0.5 (0.3 to 0.7) | 1.7 (1.1 to 2.6) | -0.17 (-1.14 to 0.8) |
| Vanuatu | 0 (0 to 0) | 5.7 (2.9 to 10.1) | 0 (0 to 0) | 4.9 (2.6 to 8.5) | -0.55 (-0.93 to -0.17) |
| Venezuela (Bolivarian Republic of) | 0.1 (0 to 0.1) | 0.6 (0.4 to 0.9) | 0 (0 to 0.1) | 0.2 (0.1 to 0.3) | -4.29 (-4.76 to -3.82) |
| Viet Nam | 0.5 (0.3 to 0.9) | 1.2 (0.7 to 2.5) | 0.4 (0.2 to 0.6) | 0.5 (0.3 to 0.8) | -3.17 (-3.25 to -3.08) |
| Yemen | 0.1 (0 to 0.2) | 1.6 (0.7 to 3.5) | 0.1 (0.1 to 0.3) | 0.9 (0.5 to 1.8) | -1.9 (-2.09 to -1.7) |
| Zambia | 0 (0 to 0) | 0.7 (0.4 to 1.2) | 0 (0 to 0.1) | 0.5 (0.3 to 0.9) | -0.96 (-1.07 to -0.84) |
| Zimbabwe | 0.1 (0 to 0.1) | 1.4 (0.9 to 2) | 0.1 (0.1 to 0.2) | 1.5 (0.9 to 2.4) | 0.25 (-0.06 to 0.56) |
