# Supplementary material for: Spatiotemporal patterns of rheumatic heart disease burden attributable to high systolic blood pressure, high sodium diet, and lead exposure (1990 to 2019): a longitudinal observational study
Source: Front Nutr. 2024 Sep 26;11:1419349. doi: 10.3389/fnut.2024.1419349 (PMC11466049; doi:10.3389/fnut.2024.1419349)
Supplement: Supplementary file 2 [file Table_2.docx]

**Supplementary table 2. Disability-adjusted life year of rheumatic heart disease due to high systolic blood pressure**

| **Location** | **1990 Counts**  **(thousand)** | **Age-standardised rate (per 100 000 population), 1990** | **2019 Counts**  **(thousand)** | **Age-standardised rate (per 100 000 population), 2019** | **Average annual percent change** |
| --- | --- | --- | --- | --- | --- |
| Afghanistan | 6.1 (2.8 to 12.4) | 82.4 (37 to 170.2) | 9.5 (5.1 to 17.2) | 47.1 (24.8 to 90.5) | -1.86 (-1.95 to -1.78) |
| Albania | 1.3 (0.9 to 1.9) | 52.1 (35.9 to 78.5) | 0.6 (0.4 to 0.9) | 18.1 (11.3 to 27) | -3.65 (-3.97 to -3.33) |
| Algeria | 4.8 (2.8 to 7.9) | 31.4 (18.9 to 50.3) | 4.8 (2.8 to 7.7) | 11.9 (7.4 to 19.1) | -3.33 (-3.43 to -3.22) |
| American Samoa | 0 (0 to 0) | 73.2 (44.7 to 121.1) | 0 (0 to 0) | 57.6 (36.4 to 91.9) | -0.91 (-1.23 to -0.59) |
| Andorra | 0 (0 to 0) | 9.6 (5.5 to 16.3) | 0 (0 to 0) | 5.3 (3.2 to 8.7) | -1.95 (-2.16 to -1.73) |
| Angola | 3.8 (2.1 to 6) | 65.7 (36.9 to 108.8) | 6.3 (3.6 to 9.7) | 37.3 (22.5 to 55.9) | -1.92 (-2.12 to -1.71) |
| Antigua and Barbuda | 0 (0 to 0) | 23.2 (13.9 to 37.4) | 0 (0 to 0) | 15.3 (8.8 to 25) | -1.31 (-1.61 to -1.01) |
| Argentina | 8 (5 to 13.2) | 25.4 (15.8 to 42.2) | 8.7 (5.4 to 14.1) | 16.9 (10.5 to 26.8) | -1.44 (-1.74 to -1.13) |
| Armenia | 2.9 (1.9 to 4.3) | 89.8 (60.2 to 135) | 1.2 (0.8 to 1.8) | 31.2 (20.6 to 47.9) | -3.64 (-4.08 to -3.2) |
| Australia | 2.7 (1.8 to 4.3) | 14.2 (9.6 to 22.3) | 2 (1.3 to 3.5) | 5.3 (3.4 to 8.8) | -3.38 (-3.62 to -3.15) |
| Austria | 2 (1.3 to 3.2) | 17.6 (11.3 to 27.2) | 1.2 (0.7 to 2.2) | 6.5 (4.1 to 11) | -3.37 (-3.58 to -3.16) |
| Azerbaijan | 3.2 (1.9 to 5) | 49.3 (31.3 to 76.4) | 2.7 (1.5 to 4.2) | 23.1 (13.8 to 35.5) | -2.65 (-3.03 to -2.27) |
| Bahamas | 0 (0 to 0.1) | 21.8 (12.9 to 35.6) | 0.1 (0 to 0.1) | 16.5 (9.3 to 26) | -0.9 (-1.02 to -0.77) |
| Bahrain | 0 (0 to 0.1) | 15 (9.6 to 25.9) | 0.1 (0.1 to 0.1) | 6.6 (4.2 to 10) | -2.8 (-3.22 to -2.39) |
| Bangladesh | 20 (10.5 to 33.1) | 28.8 (16.6 to 44.7) | 32.8 (18.5 to 50.2) | 21.8 (12.9 to 32.8) | -0.77 (-1.27 to -0.27) |
| Barbados | 0.1 (0 to 0.1) | 20.4 (12.5 to 33.3) | 0.1 (0 to 0.1) | 16.3 (9.5 to 25.9) | -0.72 (-1.04 to -0.4) |
| Belarus | 10.9 (7.4 to 15.6) | 87 (59.3 to 126.1) | 3.2 (2 to 5) | 21.9 (13.8 to 34.9) | -4.62 (-5.36 to -3.87) |
| Belgium | 0.8 (0.5 to 1.3) | 5.1 (3.1 to 8.5) | 1.7 (1 to 3.1) | 6.9 (4.2 to 11.7) | 1.13 (0.87 to 1.39) |
| Belize | 0 (0 to 0) | 17.9 (10.8 to 28.2) | 0.1 (0 to 0.1) | 15.4 (8.7 to 25.5) | -0.54 (-0.77 to -0.31) |
| Benin | 1.1 (0.6 to 1.7) | 45.4 (27.4 to 72.9) | 2 (1.2 to 3.2) | 27.7 (18.1 to 42.5) | -1.71 (-1.89 to -1.54) |
| Bermuda | 0 (0 to 0) | 11.2 (7.2 to 17.7) | 0 (0 to 0) | 3.2 (2 to 5.2) | -4.18 (-4.38 to -3.97) |
| Bhutan | 0.5 (0.3 to 1) | 154.6 (77.2 to 283.8) | 0.5 (0.2 to 1) | 78.5 (37.2 to 160.6) | -2.33 (-2.53 to -2.12) |
| Bolivia (Plurinational State of) | 1.2 (0.6 to 2.1) | 30.3 (15.9 to 55.3) | 1.5 (0.8 to 2.5) | 15 (8.5 to 25) | -2.39 (-2.6 to -2.17) |
| Bosnia and Herzegovina | 0.9 (0.6 to 1.4) | 21.2 (14.1 to 32.2) | 0.3 (0.2 to 0.4) | 5 (3.3 to 7.6) | -4.98 (-5.32 to -4.63) |
| Botswana | 0.5 (0.2 to 0.8) | 59.5 (32.1 to 100.2) | 0.7 (0.4 to 1) | 30.5 (17.7 to 45.6) | -2.3 (-2.42 to -2.17) |
| Brazil | 35.8 (21.9 to 55.7) | 29.8 (19.6 to 47) | 41.7 (26.5 to 64.5) | 17.1 (10.9 to 26.6) | -1.85 (-2 to -1.7) |
| Brunei Darussalam | 0 (0 to 0.1) | 31.8 (20.5 to 47.5) | 0.1 (0 to 0.1) | 14.5 (9.7 to 22.5) | -2.71 (-2.83 to -2.59) |
| Bulgaria | 8.4 (5.5 to 12.3) | 71.5 (48 to 103.6) | 1.8 (1.1 to 2.9) | 16.5 (10.2 to 26) | -4.85 (-5.23 to -4.46) |
| Burkina Faso | 1.4 (0.8 to 2.4) | 26.9 (16.2 to 47.9) | 2.5 (1.5 to 4.2) | 21.1 (13.4 to 32.6) | -0.76 (-0.82 to -0.7) |
| Burundi | 1.4 (0.7 to 2.2) | 41.3 (23 to 64.4) | 2.1 (1.1 to 3.4) | 28.9 (17.3 to 45.5) | -1.23 (-1.28 to -1.18) |
| Cabo Verde | 0.1 (0.1 to 0.2) | 63.6 (42.8 to 97.1) | 0.1 (0.1 to 0.2) | 21.8 (14.2 to 32.7) | -3.68 (-3.92 to -3.45) |
| Cambodia | 3.5 (1.7 to 5.8) | 52.7 (29.2 to 82.7) | 2.3 (1.2 to 4) | 15.4 (8 to 25.4) | -4.19 (-4.33 to -4.04) |
| Cameroon | 1.6 (0.9 to 2.7) | 31 (18.1 to 54.6) | 4.5 (2.6 to 7.8) | 25 (15.2 to 42.2) | -0.7 (-0.84 to -0.57) |
| Canada | 4 (2.6 to 6.5) | 12.3 (8.1 to 20) | 3.4 (2 to 6.1) | 5.1 (3 to 8.9) | -2.98 (-3.17 to -2.79) |
| Central African Republic | 1.4 (0.8 to 2.2) | 90.7 (50.1 to 144.1) | 2.1 (1.1 to 3.5) | 68.8 (35.9 to 118.8) | -0.93 (-1.01 to -0.84) |
| Chad | 1.5 (0.8 to 2.7) | 47.3 (26.7 to 85.3) | 2.3 (1.3 to 4) | 30.1 (17.7 to 54.3) | -1.52 (-1.65 to -1.39) |
| Chile | 3.9 (2.7 to 5.7) | 35.6 (24.8 to 51.3) | 1.4 (0.9 to 2.2) | 6.1 (4.1 to 9.4) | -5.85 (-6.29 to -5.4) |
| China | 742.3 (462.4 to 1155.5) | 83.3 (52.1 to 137.3) | 450.6 (294.9 to 694.3) | 23.2 (15.3 to 35.5) | -4.4 (-4.63 to -4.18) |
| Colombia | 2.6 (1.7 to 4.1) | 12.4 (8.1 to 19.8) | 1.3 (0.8 to 2.1) | 2.4 (1.5 to 3.9) | -5.57 (-6.02 to -5.12) |
| Comoros | 0.1 (0 to 0.2) | 36.5 (18.7 to 61.6) | 0.2 (0.1 to 0.2) | 24.8 (14.8 to 38.9) | -1.32 (-1.69 to -0.95) |
| Congo | 1 (0.6 to 1.5) | 69 (41.5 to 106.5) | 1.3 (0.8 to 2.1) | 35.1 (21.8 to 52.6) | -2.36 (-2.65 to -2.06) |
| Cook Islands | 0 (0 to 0) | 28.9 (16.6 to 46.9) | 0 (0 to 0) | 19.1 (11.2 to 30.8) | -1.42 (-1.65 to -1.19) |
| Costa Rica | 0.8 (0.5 to 1.2) | 34.9 (22.9 to 52.6) | 1.2 (0.7 to 1.8) | 22.7 (14 to 34.6) | -1.5 (-1.79 to -1.22) |
| Croatia | 2.2 (1.5 to 3.3) | 34.6 (23.1 to 52.1) | 0.6 (0.4 to 1) | 7.4 (4.7 to 11.8) | -4.77 (-5.98 to -3.55) |
| Cuba | 2.7 (1.5 to 4.4) | 25.2 (14.6 to 40.4) | 2 (1.2 to 3.1) | 13.2 (7.4 to 21.3) | -2.16 (-2.5 to -1.83) |
| Cyprus | 0.3 (0.2 to 0.5) | 34.5 (20.1 to 64.4) | 0.2 (0.1 to 0.4) | 11.4 (6.8 to 19.1) | -3.72 (-4.11 to -3.32) |
| Czechia | 6.5 (4.3 to 9.9) | 49.9 (33.5 to 73.6) | 1.4 (0.8 to 2.3) | 7.5 (4.6 to 12) | -6.33 (-6.58 to -6.08) |
| Côte d'Ivoire | 2.7 (1.6 to 4.3) | 45.5 (28.1 to 71.1) | 4.5 (2.5 to 7.2) | 26.6 (17 to 42) | -1.85 (-1.99 to -1.72) |
| Democratic People's Republic of Korea | 17 (10.4 to 25.9) | 93.2 (59.1 to 141.6) | 12.3 (6.5 to 22.1) | 38.5 (20.4 to 69.4) | -2.97 (-3.18 to -2.77) |
| Democratic Republic of the Congo | 13.8 (8.1 to 21.7) | 64.5 (38.6 to 102.6) | 19 (10.3 to 32.5) | 37.8 (21.2 to 67.2) | -1.83 (-1.99 to -1.66) |
| Denmark | 1.6 (1 to 2.4) | 19.8 (13.2 to 29.8) | 0.4 (0.2 to 0.7) | 3.5 (2.2 to 5.6) | -5.72 (-6.89 to -4.52) |
| Djibouti | 0.1 (0 to 0.1) | 28.2 (15.9 to 47) | 0.2 (0.1 to 0.4) | 22.9 (13.6 to 36.9) | -0.71 (-0.86 to -0.56) |
| Dominica | 0 (0 to 0) | 34.9 (21.1 to 55) | 0 (0 to 0) | 22.2 (13 to 37.2) | -1.63 (-1.89 to -1.37) |
| Dominican Republic | 1.1 (0.6 to 1.8) | 20.8 (12.3 to 33.7) | 1.8 (1 to 2.9) | 16.8 (9.7 to 26.9) | -0.71 (-1.19 to -0.22) |
| Ecuador | 1 (0.6 to 1.8) | 15.5 (9.2 to 27.2) | 1.7 (0.9 to 2.7) | 10.1 (5.7 to 16.4) | -1.61 (-1.83 to -1.39) |
| Egypt | 12.6 (6.3 to 23.8) | 32.3 (16.9 to 63.9) | 16.6 (9.2 to 27.1) | 19.8 (11.4 to 32.5) | -1.63 (-1.89 to -1.36) |
| El Salvador | 0.6 (0.3 to 1) | 15.8 (9.6 to 26.5) | 0.7 (0.4 to 1.2) | 11.7 (6.8 to 19.7) | -1 (-1.33 to -0.67) |
| Equatorial Guinea | 0.2 (0.1 to 0.3) | 74.4 (39.2 to 135.9) | 0.2 (0.1 to 0.4) | 26.6 (15.5 to 42.4) | -3.48 (-3.62 to -3.35) |
| Eritrea | 0.6 (0.3 to 1) | 39.4 (21.9 to 67.7) | 1.2 (0.6 to 1.9) | 27.1 (16.2 to 44) | -1.28 (-1.41 to -1.16) |
| Estonia | 1.2 (0.8 to 1.7) | 59.6 (39.2 to 87.1) | 0.2 (0.1 to 0.4) | 10.7 (6.4 to 17) | -5.62 (-6.26 to -4.97) |
| Eswatini | 0.2 (0.1 to 0.4) | 52.3 (29.4 to 82.6) | 0.3 (0.2 to 0.5) | 33.8 (20.9 to 53.6) | -1.54 (-1.93 to -1.14) |
| Ethiopia | 5.6 (2.9 to 9.2) | 21.1 (11.6 to 33.8) | 12.2 (6.5 to 20.4) | 18.3 (11.1 to 29.4) | -0.47 (-0.61 to -0.33) |
| Fiji | 0.6 (0.3 to 1) | 107.9 (64.6 to 162) | 0.7 (0.4 to 1.1) | 80.2 (50.9 to 125.9) | -1.06 (-1.36 to -0.77) |
| Finland | 1.1 (0.7 to 1.6) | 15.2 (10.1 to 23) | 0.3 (0.2 to 0.5) | 2.3 (1.5 to 3.8) | -6.25 (-6.53 to -5.96) |
| France | 11.1 (7.3 to 17.9) | 13.7 (9.4 to 22.1) | 8.9 (5.2 to 17) | 6.4 (4 to 10.9) | -2.58 (-2.8 to -2.35) |
| Gabon | 0.3 (0.2 to 0.4) | 41.8 (24.2 to 65.9) | 0.3 (0.2 to 0.5) | 23.6 (13.9 to 37.7) | -1.92 (-2.03 to -1.8) |
| Gambia | 0.2 (0.1 to 0.3) | 39.1 (23.4 to 62.3) | 0.4 (0.2 to 0.6) | 29 (18.4 to 43.7) | -0.91 (-1.45 to -0.37) |
| Georgia | 3.5 (2.4 to 5) | 56.5 (38.4 to 83.2) | 2.6 (1.7 to 3.8) | 50.2 (33.2 to 72.1) | -0.37 (-0.89 to 0.15) |
| Germany | 30.8 (20.9 to 48.9) | 24.8 (16.9 to 38.2) | 17.5 (10.3 to 32.4) | 8.7 (5.4 to 15) | -3.36 (-3.84 to -2.88) |
| Ghana | 2.8 (1.7 to 4.5) | 33.1 (20.3 to 53) | 4.3 (2.5 to 7.2) | 19.6 (12.7 to 31.6) | -1.78 (-1.94 to -1.62) |
| Greece | 1.2 (0.8 to 2) | 8.4 (5.7 to 13.4) | 0.6 (0.4 to 1.1) | 2.6 (1.7 to 4.5) | -3.83 (-4.14 to -3.52) |
| Greenland | 0 (0 to 0) | 26.8 (16.5 to 47.7) | 0 (0 to 0) | 7.8 (4.9 to 12.7) | -4.17 (-4.59 to -3.75) |
| Grenada | 0 (0 to 0.1) | 53.3 (32 to 85.7) | 0 (0 to 0) | 25.9 (15.5 to 41.2) | -2.42 (-2.7 to -2.14) |
| Guam | 0 (0 to 0.1) | 30.2 (18.6 to 49.5) | 0 (0 to 0.1) | 27.5 (16.9 to 43.1) | -0.45 (-0.85 to -0.06) |
| Guatemala | 0.5 (0.2 to 0.8) | 9.5 (5.4 to 16.3) | 1.9 (1 to 3.3) | 12.4 (7.1 to 20.5) | 0.72 (0.31 to 1.14) |
| Guinea | 1.8 (1.1 to 3.1) | 48.7 (28.5 to 85.5) | 2.3 (1.3 to 3.6) | 30.9 (18.7 to 47.9) | -1.53 (-1.68 to -1.38) |
| Guinea-Bissau | 0.4 (0.2 to 0.6) | 78.8 (45.2 to 124.7) | 0.5 (0.3 to 0.7) | 41.4 (25.1 to 65.3) | -2.22 (-2.34 to -2.1) |
| Guyana | 0.2 (0.1 to 0.4) | 39.1 (22.7 to 61.8) | 0.2 (0.1 to 0.3) | 25 (14 to 39.7) | -1.53 (-1.86 to -1.2) |
| Haiti | 7.6 (3.9 to 13.1) | 167.9 (88.7 to 290.4) | 10.3 (5.2 to 17.7) | 97.9 (49.9 to 171.4) | -1.83 (-1.94 to -1.71) |
| Honduras | 0.4 (0.2 to 0.6) | 13.1 (8 to 21.9) | 0.9 (0.5 to 1.5) | 11.8 (6.8 to 18.4) | -0.33 (-0.54 to -0.12) |
| Hungary | 9.4 (6.5 to 13.3) | 66.4 (46.6 to 94.6) | 1.4 (0.9 to 2.3) | 7.9 (5.1 to 12.4) | -7.1 (-7.46 to -6.74) |
| Iceland | 0 (0 to 0) | 4.9 (3 to 7.9) | 0 (0 to 0) | 2.5 (1.6 to 4) | -2.34 (-2.58 to -2.11) |
| India | 876.6 (544 to 1296.5) | 149.6 (94.5 to 224.9) | 1031.4 (664.9 to 1464.1) | 80.7 (53.1 to 114.2) | -2.11 (-2.56 to -1.65) |
| Indonesia | 26 (15.8 to 38.2) | 19.7 (12.7 to 29) | 16.8 (11.7 to 25.5) | 6.9 (4.8 to 10.3) | -3.56 (-3.66 to -3.46) |
| Iran (Islamic Republic of) | 7.4 (4.6 to 12.7) | 22.9 (14.3 to 42) | 11.1 (7.3 to 17.3) | 12.6 (8.5 to 19.7) | -2.01 (-2.27 to -1.74) |
| Iraq | 5.1 (2.9 to 8.3) | 51.1 (29.9 to 83.8) | 6.3 (3.9 to 9.7) | 19.3 (12.9 to 29) | -3.33 (-3.56 to -3.1) |
| Ireland | 0.6 (0.4 to 0.9) | 15.9 (10.4 to 24) | 0.3 (0.2 to 0.5) | 4.5 (2.9 to 7.1) | -4.2 (-4.67 to -3.73) |
| Israel | 0.9 (0.6 to 1.4) | 18.9 (13.1 to 28.9) | 1.1 (0.7 to 1.7) | 9.7 (6.4 to 14.9) | -2.37 (-3.48 to -1.26) |
| Italy | 21.6 (14.6 to 31.7) | 25.6 (17.3 to 37.6) | 10.3 (6.2 to 18) | 7.4 (4.8 to 12.2) | -4.13 (-4.36 to -3.9) |
| Jamaica | 0.3 (0.2 to 0.5) | 16.6 (9.5 to 27.9) | 0.5 (0.2 to 0.8) | 15.4 (8.2 to 26.2) | 0.02 (-0.24 to 0.28) |
| Japan | 19.8 (13.6 to 29.7) | 12 (8.2 to 18.2) | 13.2 (7.5 to 25.4) | 3.4 (2.2 to 5.9) | -4.27 (-4.49 to -4.05) |
| Jordan | 0.2 (0.1 to 0.3) | 11 (6.7 to 18.1) | 0.3 (0.2 to 0.4) | 3.2 (2 to 4.8) | -4.27 (-4.57 to -3.96) |
| Kazakhstan | 16.5 (11.3 to 23.2) | 110 (73.7 to 153.7) | 4.8 (3.1 to 7.1) | 24.4 (15.8 to 36) | -5.1 (-5.51 to -4.69) |
| Kenya | 3.1 (1.7 to 4.9) | 25.6 (14.9 to 39.3) | 7.2 (4 to 11.1) | 21 (12.2 to 31.8) | -0.69 (-0.73 to -0.64) |
| Kiribati | 0.1 (0.1 to 0.2) | 184.2 (104.4 to 304.5) | 0.1 (0.1 to 0.2) | 128.3 (67.7 to 220) | -1.25 (-1.32 to -1.18) |
| Kuwait | 0.1 (0.1 to 0.2) | 11.6 (7.5 to 17.7) | 0.1 (0.1 to 0.2) | 2.7 (1.7 to 4) | -4.79 (-5.59 to -3.99) |
| Kyrgyzstan | 3.3 (1.9 to 4.9) | 91.3 (54.9 to 138.8) | 1.7 (0.9 to 2.7) | 26.8 (15.5 to 43.8) | -4.09 (-4.7 to -3.47) |
| Lao People's Democratic Republic | 2 (1 to 3.2) | 69.4 (38.2 to 114.4) | 2 (1 to 3.5) | 31 (16.7 to 51.6) | -2.77 (-2.88 to -2.65) |
| Latvia | 2.9 (1.9 to 4.1) | 86.1 (57.9 to 122) | 0.5 (0.3 to 0.7) | 15.2 (9.6 to 22.8) | -5.64 (-7.32 to -3.92) |
| Lebanon | 0.4 (0.2 to 0.8) | 17.5 (10 to 32.5) | 0.4 (0.2 to 0.6) | 6.7 (3.3 to 10.7) | -3.27 (-3.43 to -3.11) |
| Lesotho | 0.6 (0.3 to 1) | 49.9 (25.1 to 84.1) | 0.7 (0.4 to 1.1) | 41.4 (23.6 to 65) | -0.63 (-0.75 to -0.51) |
| Liberia | 0.6 (0.3 to 0.9) | 45.3 (28 to 74) | 0.8 (0.5 to 1.3) | 26.5 (16 to 42.7) | -1.8 (-1.97 to -1.62) |
| Libya | 0.6 (0.3 to 1) | 22 (12.5 to 39.5) | 1.1 (0.6 to 1.8) | 15.1 (9.1 to 25.5) | -1.16 (-1.62 to -0.7) |
| Lithuania | 4.2 (2.9 to 5.8) | 95.9 (66.5 to 132.9) | 0.8 (0.5 to 1.2) | 17.8 (11.1 to 27.1) | -5.87 (-6.63 to -5.1) |
| Luxembourg | 0.1 (0 to 0.1) | 13.7 (9.3 to 21) | 0.1 (0 to 0.1) | 6.1 (3.9 to 9.9) | -2.78 (-2.94 to -2.63) |
| Madagascar | 3.6 (1.9 to 5.9) | 47.5 (28.9 to 76.9) | 7 (3.8 to 11.6) | 40.1 (24.1 to 62.1) | -0.57 (-0.68 to -0.46) |
| Malawi | 2.3 (1.3 to 3.6) | 40.1 (24.8 to 60.4) | 3.5 (2 to 5.6) | 29.8 (18.8 to 44.6) | -1.01 (-1.08 to -0.94) |
| Malaysia | 5.1 (3.1 to 7.8) | 40 (26.1 to 59.8) | 4.7 (2.9 to 7.1) | 14.5 (9.2 to 21.5) | -3.43 (-3.79 to -3.06) |
| Maldives | 0.1 (0 to 0.1) | 47.4 (26.6 to 78.2) | 0.1 (0 to 0.1) | 12.3 (7.7 to 18.4) | -4.59 (-4.78 to -4.39) |
| Mali | 2.6 (1.5 to 4.2) | 52.5 (31.2 to 84.2) | 2.9 (1.6 to 4.7) | 25.5 (15.3 to 39.5) | -2.44 (-2.66 to -2.21) |
| Malta | 0.1 (0 to 0.1) | 14.3 (9.6 to 22.5) | 0 (0 to 0.1) | 5.9 (3.8 to 9.4) | -3 (-3.16 to -2.84) |
| Marshall Islands | 0 (0 to 0) | 109.3 (57.7 to 203.5) | 0 (0 to 0.1) | 89.8 (45.8 to 157.9) | -0.69 (-0.76 to -0.62) |
| Mauritania | 0.5 (0.3 to 0.9) | 46.1 (29.4 to 75.8) | 0.5 (0.3 to 0.8) | 19.2 (11.8 to 30.4) | -3 (-3.16 to -2.84) |
| Mauritius | 0.3 (0.2 to 0.5) | 29.3 (19.2 to 46.5) | 0.2 (0.1 to 0.3) | 10.5 (6.1 to 17) | -3.35 (-4 to -2.7) |
| Mexico | 16.5 (10.4 to 25.3) | 29.9 (19.3 to 47.8) | 11.2 (7.4 to 17.1) | 8.9 (5.9 to 13.7) | -4.17 (-4.37 to -3.98) |
| Micronesia (Federated States of) | 0.1 (0 to 0.1) | 119.7 (63.2 to 199.3) | 0.1 (0 to 0.1) | 80.7 (36 to 151) | -1.36 (-1.45 to -1.27) |
| Monaco | 0 (0 to 0) | 5.7 (3.6 to 9.1) | 0 (0 to 0) | 3.5 (2.2 to 5.6) | -1.7 (-1.82 to -1.59) |
| Mongolia | 1.5 (0.9 to 2.7) | 122.3 (74.5 to 220) | 1.7 (1.1 to 2.6) | 54 (35.8 to 80) | -2.8 (-2.96 to -2.64) |
| Montenegro | 0.1 (0.1 to 0.2) | 20.3 (13.3 to 31.1) | 0.1 (0.1 to 0.2) | 11.6 (7.8 to 16.9) | -1.82 (-1.98 to -1.65) |
| Morocco | 9 (5.2 to 15.3) | 51.9 (29.9 to 94) | 8.8 (5.6 to 13.9) | 24.7 (16.1 to 39) | -2.54 (-2.65 to -2.43) |
| Mozambique | 3.2 (1.9 to 5) | 40.3 (25 to 62.7) | 5.9 (3.4 to 9.3) | 34.5 (21.7 to 51.9) | -0.52 (-0.57 to -0.47) |
| Myanmar | 14.5 (7.7 to 23.1) | 47.9 (28.1 to 78.5) | 12 (6.9 to 18.9) | 22.5 (13.3 to 35.2) | -2.61 (-2.73 to -2.49) |
| Namibia | 0.5 (0.3 to 0.8) | 55.5 (32.2 to 94.4) | 0.5 (0.3 to 0.9) | 27.2 (16.8 to 42.1) | -2.4 (-2.54 to -2.26) |
| Nauru | 0 (0 to 0) | 126 (68.2 to 208.3) | 0 (0 to 0) | 110.1 (60.9 to 164.3) | -0.47 (-0.64 to -0.3) |
| Nepal | 20.3 (10.9 to 34.1) | 162.8 (87.2 to 280.2) | 17.6 (9.2 to 28.8) | 71.3 (37.5 to 117.1) | -2.87 (-3.06 to -2.68) |
| Netherlands | 1.1 (0.7 to 1.8) | 5.7 (3.8 to 8.8) | 1.2 (0.7 to 2.1) | 3.5 (2.2 to 6) | -1.62 (-2.15 to -1.08) |
| New Zealand | 1.1 (0.8 to 1.7) | 29.9 (20.4 to 45) | 0.8 (0.5 to 1.2) | 11.7 (7.8 to 17.1) | -3.19 (-3.48 to -2.89) |
| Nicaragua | 0.6 (0.3 to 1) | 23.4 (14.4 to 39.2) | 0.9 (0.5 to 1.5) | 14.6 (8.6 to 23.5) | -1.67 (-1.89 to -1.46) |
| Niger | 2.1 (1.1 to 4) | 56.9 (32 to 107.9) | 3.6 (2 to 6.3) | 33.5 (19.1 to 59.5) | -1.82 (-1.93 to -1.7) |
| Nigeria | 20.5 (11.3 to 37.8) | 40 (22.3 to 75.3) | 33.1 (19.5 to 55.9) | 25.9 (16.1 to 43.8) | -1.44 (-1.65 to -1.22) |
| Niue | 0 (0 to 0) | 68.3 (38.6 to 105.4) | 0 (0 to 0) | 41.5 (23.5 to 68.7) | -1.75 (-1.93 to -1.57) |
| North Macedonia | 0.8 (0.5 to 1.3) | 38.7 (25.7 to 64) | 0.3 (0.2 to 0.5) | 10.2 (6.8 to 16) | -4.48 (-4.74 to -4.22) |
| Northern Mariana Islands | 0 (0 to 0) | 31.9 (17.6 to 55.1) | 0 (0 to 0) | 27.8 (16.5 to 43.5) | -0.46 (-0.58 to -0.33) |
| Norway | 0.8 (0.5 to 1.3) | 11.4 (7.5 to 17.9) | 0.3 (0.2 to 0.6) | 3.5 (2.2 to 5.9) | -3.95 (-4.48 to -3.43) |
| Oman | 0.1 (0 to 0.1) | 8.5 (4.1 to 18.7) | 0.1 (0.1 to 0.2) | 3.6 (2.3 to 5.9) | -2.87 (-3.19 to -2.55) |
| Pakistan | 121.2 (73.9 to 189.1) | 180.1 (111 to 277.3) | 227 (138.7 to 335.3) | 149.1 (95.6 to 219.4) | -0.66 (-0.74 to -0.58) |
| Palau | 0 (0 to 0) | 38.6 (20.3 to 65.4) | 0 (0 to 0) | 32.3 (18.6 to 49.8) | -0.63 (-0.75 to -0.5) |
| Palestine | 0.2 (0.1 to 0.3) | 16 (9.4 to 26.2) | 0.3 (0.2 to 0.5) | 8.1 (4.9 to 13) | -2.34 (-2.43 to -2.26) |
| Panama | 0.5 (0.3 to 0.8) | 24.3 (14.3 to 38) | 0.6 (0.3 to 0.9) | 13.4 (7.6 to 21.1) | -1.94 (-2.25 to -1.62) |
| Papua New Guinea | 2.1 (0.8 to 4.3) | 81.5 (32.5 to 166) | 7.5 (3.3 to 14.3) | 99.7 (44.3 to 186) | 0.64 (0.53 to 0.76) |
| Paraguay | 0.6 (0.3 to 1) | 20 (12.4 to 32.2) | 1.1 (0.6 to 1.7) | 15.6 (9.4 to 24.1) | -0.88 (-1.25 to -0.51) |
| Peru | 1.5 (0.8 to 2.7) | 10.3 (5.7 to 17.5) | 3.2 (1.8 to 5.1) | 9.2 (5.2 to 14.8) | -0.32 (-0.62 to -0.03) |
| Philippines | 7.6 (4.2 to 12.4) | 16.5 (9.9 to 26.1) | 20.1 (10.8 to 32.7) | 19 (10.6 to 31) | 0.58 (0.23 to 0.93) |
| Poland | 36.1 (25.2 to 52.2) | 83.1 (58.9 to 119.9) | 6.6 (4.2 to 9.8) | 10.2 (6.6 to 15) | -7.05 (-7.57 to -6.53) |
| Portugal | 3.5 (2.5 to 5.2) | 27.9 (19.4 to 40.7) | 1.3 (0.8 to 2.1) | 6.1 (4 to 9.5) | -5.23 (-5.76 to -4.7) |
| Puerto Rico | 0.3 (0.2 to 0.5) | 8.8 (5.7 to 13.2) | 0.2 (0.1 to 0.3) | 3.8 (2.3 to 6.1) | -2.91 (-3.28 to -2.55) |
| Qatar | 0 (0 to 0.1) | 17.1 (9.3 to 34.4) | 0.1 (0 to 0.1) | 4.6 (2.9 to 7.8) | -4.46 (-4.77 to -4.15) |
| Republic of Korea | 2.1 (1.4 to 3.3) | 6.5 (4.2 to 10.5) | 1.3 (0.8 to 2.2) | 1.5 (0.9 to 2.6) | -5.2 (-5.62 to -4.78) |
| Republic of Moldova | 4.2 (2.7 to 6.1) | 89.4 (58.3 to 132.2) | 1 (0.7 to 1.5) | 19.5 (12.4 to 28.4) | -5.2 (-6.16 to -4.23) |
| Romania | 21 (14.9 to 29.8) | 76.5 (53.3 to 108) | 3.6 (2.3 to 5.4) | 11.3 (7.5 to 16.8) | -6.4 (-6.69 to -6.11) |
| Russian Federation | 123.8 (86.4 to 180.9) | 67.8 (47.2 to 99.8) | 29.9 (19.9 to 45.6) | 13.5 (9.1 to 20.4) | -5.45 (-6.43 to -4.46) |
| Rwanda | 2.1 (1.1 to 3.3) | 50.2 (29.4 to 82.9) | 1.9 (1 to 3.2) | 21.3 (12.3 to 33.4) | -2.94 (-3.1 to -2.78) |
| Saint Kitts and Nevis | 0 (0 to 0) | 36.6 (21.3 to 58.1) | 0 (0 to 0) | 8.6 (4.4 to 14) | -4.7 (-4.94 to -4.47) |
| Saint Lucia | 0 (0 to 0.1) | 48.7 (30.9 to 72.8) | 0 (0 to 0.1) | 22.6 (13.6 to 35.3) | -2.56 (-2.76 to -2.37) |
| Saint Vincent and the Grenadines | 0 (0 to 0) | 34.6 (20 to 56.6) | 0 (0 to 0) | 22.4 (13 to 35.4) | -1.43 (-1.66 to -1.21) |
| Samoa | 0.1 (0 to 0.1) | 76.4 (41.2 to 130.4) | 0.1 (0.1 to 0.2) | 64.4 (34.8 to 107.8) | -0.58 (-0.73 to -0.43) |
| San Marino | 0 (0 to 0) | 16.2 (9.9 to 26.8) | 0 (0 to 0) | 11.1 (6.2 to 19.1) | -1.26 (-1.46 to -1.06) |
| Sao Tome and Principe | 0 (0 to 0.1) | 64.8 (41.8 to 93.3) | 0.1 (0 to 0.1) | 47.3 (29.6 to 69.5) | -1.09 (-1.22 to -0.96) |
| Saudi Arabia | 2.1 (1 to 4.5) | 22.4 (11.9 to 49) | 2.3 (1.3 to 3.9) | 7 (4.1 to 10.6) | -3.98 (-4.34 to -3.63) |
| Senegal | 1.8 (1.1 to 2.8) | 45.9 (28 to 71.8) | 2.5 (1.5 to 4) | 25.3 (16 to 39.8) | -2.1 (-2.38 to -1.82) |
| Serbia | 2.5 (1.5 to 4.5) | 21.6 (13.6 to 40.1) | 1 (0.7 to 1.6) | 6.9 (4.5 to 10.5) | -3.79 (-4.14 to -3.44) |
| Seychelles | 0 (0 to 0) | 50 (34.5 to 72.4) | 0 (0 to 0) | 12 (7.5 to 18.6) | -4.83 (-5 to -4.66) |
| Sierra Leone | 1.2 (0.7 to 1.9) | 54.7 (33.7 to 86.3) | 1.8 (1.1 to 2.7) | 34.7 (22 to 51.2) | -1.57 (-1.75 to -1.38) |
| Singapore | 0.4 (0.3 to 0.6) | 15.4 (9.8 to 23.9) | 0.1 (0.1 to 0.2) | 1.6 (1 to 2.7) | -7.5 (-7.76 to -7.24) |
| Slovakia | 1.4 (0.9 to 1.9) | 22.9 (15.2 to 33.2) | 0.8 (0.5 to 1.3) | 9.4 (6.2 to 14.2) | -3.05 (-3.33 to -2.77) |
| Slovenia | 0.9 (0.6 to 1.3) | 35.8 (22.7 to 54.5) | 0.5 (0.3 to 0.9) | 12.1 (7.4 to 19.7) | -3.69 (-4.13 to -3.24) |
| Solomon Islands | 0.3 (0.1 to 0.6) | 149.4 (65.4 to 270.2) | 0.6 (0.2 to 1.1) | 116.3 (50.6 to 211.7) | -0.86 (-1.03 to -0.69) |
| Somalia | 1.8 (0.8 to 3) | 46.5 (24.2 to 79.3) | 4.1 (2 to 7.3) | 39.4 (20 to 71.8) | -0.56 (-0.69 to -0.43) |
| South Africa | 14.9 (8.6 to 22) | 48.5 (29.9 to 69.9) | 15.1 (9 to 22.6) | 26.1 (16.6 to 38.6) | -2.23 (-2.71 to -1.75) |
| South Sudan | 1 (0.5 to 1.8) | 29.9 (15.9 to 50.7) | 1.4 (0.7 to 2.4) | 25.2 (14.2 to 45) | -0.56 (-0.65 to -0.48) |
| Spain | 16.7 (11 to 26.5) | 31.4 (20.6 to 48.2) | 9.2 (5.5 to 16.3) | 9.6 (6.1 to 15.8) | -4.02 (-4.18 to -3.87) |
| Sri Lanka | 3.2 (2 to 4.8) | 23.6 (15.5 to 34.6) | 1.6 (1 to 2.5) | 6.6 (4.2 to 10) | -4.33 (-4.8 to -3.86) |
| Sudan | 7.7 (4 to 15) | 63.4 (32.9 to 130.5) | 8.2 (4.6 to 14.6) | 30.1 (18.2 to 55.5) | -2.52 (-2.61 to -2.42) |
| Suriname | 0.1 (0 to 0.1) | 19.2 (11.1 to 30.4) | 0.1 (0 to 0.1) | 12.3 (7 to 20.3) | -1.66 (-2.02 to -1.31) |
| Sweden | 1.5 (0.9 to 2.4) | 9.5 (6.2 to 15.4) | 0.7 (0.4 to 1.3) | 3.3 (2.1 to 5.7) | -3.57 (-3.84 to -3.29) |
| Switzerland | 1.3 (0.8 to 2.3) | 12.3 (8.1 to 21.1) | 0.4 (0.3 to 0.9) | 2.4 (1.5 to 4.3) | -5.47 (-5.7 to -5.24) |
| Syrian Arab Republic | 5.6 (3.3 to 9.4) | 79.3 (46.6 to 138.3) | 2.1 (1.3 to 3.4) | 16 (9.8 to 26.3) | -5.44 (-5.86 to -5.01) |
| Taiwan (Province of China) | 4.3 (2.8 to 6.6) | 26.3 (17.6 to 40.5) | 1.6 (1 to 2.7) | 4.3 (2.6 to 7) | -6.11 (-6.4 to -5.82) |
| Tajikistan | 3.7 (2.3 to 5.8) | 103.5 (65.6 to 155.4) | 3.3 (2 to 5.3) | 41 (25.3 to 63.7) | -3.09 (-3.65 to -2.53) |
| Thailand | 15 (8.5 to 27.1) | 31 (18.8 to 53.1) | 5.1 (3 to 8.4) | 5.7 (3.3 to 9.5) | -5.7 (-6.02 to -5.39) |
| Timor-Leste | 0.3 (0.1 to 0.5) | 48.8 (26.5 to 88) | 0.3 (0.2 to 0.5) | 30.9 (17.4 to 52.7) | -1.57 (-1.95 to -1.19) |
| Togo | 0.8 (0.5 to 1.2) | 46.7 (29.2 to 70) | 1.3 (0.8 to 2) | 25.9 (16 to 40.5) | -2 (-2.14 to -1.86) |
| Tokelau | 0 (0 to 0) | 76.1 (38.3 to 134.8) | 0 (0 to 0) | 52.5 (29.1 to 88.2) | -1.31 (-1.44 to -1.19) |
| Tonga | 0 (0 to 0) | 40.8 (23.2 to 62.9) | 0 (0 to 0) | 34 (21.1 to 54.6) | -0.57 (-0.78 to -0.36) |
| Trinidad and Tobago | 0.3 (0.1 to 0.4) | 24.4 (14.9 to 38.1) | 0.3 (0.2 to 0.5) | 17.3 (10.1 to 27.4) | -1.12 (-1.49 to -0.76) |
| Tunisia | 1 (0.6 to 1.7) | 16.4 (9.8 to 29.2) | 0.9 (0.5 to 1.4) | 6.8 (4.1 to 11.2) | -2.97 (-3.05 to -2.88) |
| Turkey | NA | NA | NA | NA | NA |
| Turkmenistan | 1.9 (1.2 to 2.9) | 71.3 (46.7 to 104.2) | 1.8 (1 to 2.9) | 35.2 (20.1 to 54.7) | -2.33 (-3.96 to -0.67) |
| Tuvalu | 0 (0 to 0) | 117.8 (58.5 to 199.6) | 0 (0 to 0) | 85.4 (44.5 to 147.1) | -1.06 (-1.17 to -0.95) |
| Uganda | 3.4 (2 to 5.3) | 36.7 (22.5 to 57.1) | 5.6 (2.9 to 9.3) | 23.8 (13.9 to 36.9) | -1.51 (-1.65 to -1.38) |
| Ukraine | 18.4 (11.5 to 32.1) | 26.4 (16.9 to 45.2) | 11.5 (7.4 to 17.3) | 17.8 (11.5 to 26.8) | -1.27 (-2.53 to 0.01) |
| United Arab Emirates | 0.7 (0.3 to 1.3) | 79.8 (41.1 to 161.7) | 2.4 (1.3 to 4.2) | 28.2 (15.1 to 51.1) | -3.48 (-3.84 to -3.12) |
| United Kingdom | 19.7 (13 to 29.8) | 22.5 (15.4 to 32.8) | 5.1 (3.3 to 8.6) | 4.3 (2.9 to 6.8) | -5.58 (-5.8 to -5.36) |
| United Republic of Tanzania | 4 (2.3 to 6.2) | 27.2 (17.3 to 41.6) | 10.1 (5.8 to 16.3) | 27.1 (17 to 41.7) | 0.01 (-0.05 to 0.07) |
| United States of America | 48.1 (32.2 to 75.6) | 15.4 (10.4 to 23.6) | 27.4 (17.2 to 46.9) | 5.1 (3.3 to 8.5) | -3.72 (-3.89 to -3.56) |
| United States Virgin Islands | 0 (0 to 0) | 12.9 (8 to 21.4) | 0 (0 to 0) | 7.2 (4.5 to 11.6) | -1.98 (-2.11 to -1.84) |
| Uruguay | 0.5 (0.3 to 0.8) | 14 (9.2 to 21.6) | 0.4 (0.2 to 0.6) | 7.6 (4.9 to 11.6) | -2.09 (-2.48 to -1.71) |
| Uzbekistan | 12.1 (7 to 18.9) | 80.5 (49.7 to 123.6) | 21.8 (13.4 to 33) | 67.5 (42.1 to 102.6) | -0.4 (-1.27 to 0.49) |
| Vanuatu | 0.2 (0.1 to 0.3) | 191.6 (97.4 to 341.6) | 0.4 (0.2 to 0.7) | 174.4 (90.5 to 306) | -0.32 (-0.71 to 0.06) |
| Venezuela (Bolivarian Republic of) | 2.5 (1.6 to 3.8) | 18.7 (12.1 to 28.3) | 1.5 (0.9 to 2.5) | 5.2 (3 to 8.4) | -4.51 (-4.98 to -4.04) |
| Viet Nam | 13.1 (7.5 to 21.4) | 29.8 (17.5 to 50) | 12.1 (7.5 to 18.1) | 12.3 (7.8 to 18.2) | -3.04 (-3.18 to -2.9) |
| Yemen | 3.3 (1.6 to 6.1) | 49.9 (24.5 to 98.9) | 5.9 (3.1 to 10.4) | 30.5 (17 to 55.9) | -1.69 (-1.8 to -1.58) |
| Zambia | 0.8 (0.5 to 1.5) | 21.9 (12.6 to 37.9) | 2.1 (1.1 to 3.5) | 19.4 (11.4 to 31.1) | -0.45 (-0.54 to -0.35) |
| Zimbabwe | 2.7 (1.6 to 4) | 46.9 (30.7 to 66.8) | 5.5 (2.9 to 9) | 52.8 (30.5 to 84.1) | 0.4 (0.08 to 0.72) |
