# Supplementary material for: Spatiotemporal patterns of rheumatic heart disease burden attributable to high systolic blood pressure, high sodium diet, and lead exposure (1990 to 2019): a longitudinal observational study
Source: Front Nutr. 2024 Sep 26;11:1419349. doi: 10.3389/fnut.2024.1419349 (PMC11466049; doi:10.3389/fnut.2024.1419349)
Supplement: Supplementary file 3 [file Table_3.docx]

**Supplementary table 3. Deaths of rheumatic heart disease due to diet high in sodium**

| **location** | **1990 Counts**  **(thousand)** | **Age-standardised rate (per 100 000 population), 1990** | **2019 Counts**  **(thousand)** | **Age-standardised rate (per 100 000 population), 2019** | **Average annual percent change** |
| --- | --- | --- | --- | --- | --- |
| Afghanistan | 0 (0 to 0) | 0.1 (0 to 0.4) | 0 (0 to 0) | 0.1 (0 to 0.2) | -1.94 (-2.09 to -1.79) |
| Albania | 0 (0 to 0) | 0.5 (0.2 to 1) | 0 (0 to 0) | 0.1 (0 to 0.2) | -6.42 (-7 to -5.84) |
| Algeria | 0 (0 to 0) | 0 (0 to 0.1) | 0 (0 to 0) | 0 (0 to 0) | -3.56 (-3.64 to -3.48) |
| American Samoa | 0 (0 to 0) | 0.1 (0 to 0.5) | 0 (0 to 0) | 0.1 (0 to 0.3) | -1.05 (-1.38 to -0.73) |
| Andorra | 0 (0 to 0) | 0 (0 to 0.1) | 0 (0 to 0) | 0 (0 to 0) | -1.97 (-2.13 to -1.8) |
| Angola | 0 (0 to 0) | 0.1 (0 to 0.5) | 0 (0 to 0) | 0.1 (0 to 0.3) | -2.07 (-2.22 to -1.92) |
| Antigua and Barbuda | 0 (0 to 0) | 0 (0 to 0.1) | 0 (0 to 0) | 0 (0 to 0.1) | -2.73 (-3.05 to -2.4) |
| Argentina | 0.1 (0 to 0.2) | 0.2 (0 to 0.6) | 0 (0 to 0.1) | 0.1 (0 to 0.2) | -3.04 (-3.27 to -2.82) |
| Armenia | 0 (0 to 0) | 0.3 (0.1 to 0.7) | 0 (0 to 0) | 0.1 (0 to 0.2) | -4.62 (-5.19 to -4.05) |
| Australia | 0 (0 to 0) | 0 (0 to 0.1) | 0 (0 to 0) | 0 (0 to 0) | -2.62 (-3.18 to -2.06) |
| Austria | 0 (0 to 0) | 0.1 (0 to 0.2) | 0 (0 to 0) | 0 (0 to 0.1) | -2.06 (-2.35 to -1.77) |
| Azerbaijan | 0 (0 to 0) | 0.2 (0 to 0.4) | 0 (0 to 0) | 0.1 (0 to 0.2) | -3.75 (-4.31 to -3.18) |
| Bahamas | 0 (0 to 0) | 0 (0 to 0.1) | 0 (0 to 0) | 0 (0 to 0.1) | -2.3 (-2.48 to -2.11) |
| Bahrain | 0 (0 to 0) | 0 (0 to 0.1) | 0 (0 to 0) | 0 (0 to 0) | -2.88 (-3.34 to -2.41) |
| Bangladesh | 0 (0 to 0.1) | 0.1 (0 to 0.3) | 0.1 (0 to 0.2) | 0.1 (0 to 0.2) | -0.5 (-1.19 to 0.2) |
| Barbados | 0 (0 to 0) | 0 (0 to 0.1) | 0 (0 to 0) | 0 (0 to 0) | -2.28 (-2.64 to -1.92) |
| Belarus | 0 (0 to 0.1) | 0.1 (0 to 0.4) | 0 (0 to 0) | 0 (0 to 0.1) | -4.93 (-5.66 to -4.19) |
| Belgium | 0 (0 to 0) | 0 (0 to 0.1) | 0 (0 to 0) | 0 (0 to 0.1) | 0.95 (0.61 to 1.28) |
| Belize | 0 (0 to 0) | 0 (0 to 0.1) | 0 (0 to 0) | 0 (0 to 0.1) | -1.98 (-2.33 to -1.62) |
| Benin | 0 (0 to 0) | 0.2 (0 to 0.7) | 0 (0 to 0) | 0.1 (0 to 0.3) | -2.95 (-3.08 to -2.83) |
| Bermuda | 0 (0 to 0) | 0 (0 to 0.1) | 0 (0 to 0) | 0 (0 to 0) | -4.42 (-4.56 to -4.29) |
| Bhutan | 0 (0 to 0) | 0.5 (0 to 1.8) | 0 (0 to 0) | 0.3 (0 to 1.2) | -1.14 (-1.22 to -1.06) |
| Bolivia (Plurinational State of) | 0 (0 to 0) | 0.2 (0 to 0.6) | 0 (0 to 0) | 0.1 (0 to 0.2) | -3.52 (-3.69 to -3.36) |
| Bosnia and Herzegovina | 0 (0 to 0) | 0.2 (0.1 to 0.5) | 0 (0 to 0) | 0 (0 to 0.1) | -5.66 (-6.01 to -5.3) |
| Botswana | 0 (0 to 0) | 0.1 (0 to 0.5) | 0 (0 to 0) | 0 (0 to 0.2) | -3.98 (-4.13 to -3.83) |
| Brazil | 0.1 (0 to 0.2) | 0.1 (0 to 0.2) | 0.1 (0 to 0.2) | 0 (0 to 0.1) | -3.07 (-3.25 to -2.89) |
| Brunei Darussalam | 0 (0 to 0) | 0.3 (0 to 0.6) | 0 (0 to 0) | 0.1 (0 to 0.3) | -2.55 (-2.69 to -2.4) |
| Bulgaria | 0.1 (0 to 0.1) | 0.6 (0.2 to 1.1) | 0 (0 to 0) | 0.1 (0 to 0.3) | -5.38 (-5.79 to -4.97) |
| Burkina Faso | 0 (0 to 0) | 0.1 (0 to 0.3) | 0 (0 to 0) | 0.1 (0 to 0.2) | -0.85 (-1.04 to -0.66) |
| Burundi | 0 (0 to 0) | 0.3 (0 to 0.9) | 0 (0 to 0) | 0.1 (0 to 0.4) | -3.22 (-3.36 to -3.09) |
| Cabo Verde | 0 (0 to 0) | 0.1 (0 to 0.6) | 0 (0 to 0) | 0 (0 to 0.1) | -5.22 (-5.98 to -4.46) |
| Cambodia | 0 (0 to 0.1) | 0.4 (0.1 to 1) | 0 (0 to 0) | 0.1 (0 to 0.3) | -4.57 (-4.68 to -4.45) |
| Cameroon | 0 (0 to 0) | 0.1 (0 to 0.5) | 0 (0 to 0) | 0.1 (0 to 0.2) | -2.86 (-2.96 to -2.77) |
| Canada | 0 (0 to 0) | 0 (0 to 0.1) | 0 (0 to 0.1) | 0 (0 to 0.1) | -2.4 (-2.52 to -2.27) |
| Central African Republic | 0 (0 to 0) | 0.2 (0 to 0.8) | 0 (0 to 0) | 0.1 (0 to 0.7) | -0.49 (-0.58 to -0.4) |
| Chad | 0 (0 to 0) | 0.2 (0 to 0.7) | 0 (0 to 0) | 0.1 (0 to 0.4) | -1.87 (-1.99 to -1.75) |
| Chile | 0 (0 to 0) | 0.2 (0 to 0.4) | 0 (0 to 0) | 0 (0 to 0.1) | -5.88 (-6.31 to -5.44) |
| China | 12.5 (5 to 24.6) | 1.5 (0.6 to 3) | 5.5 (1.9 to 11.5) | 0.3 (0.1 to 0.6) | -5.68 (-6.01 to -5.34) |
| Colombia | 0 (0 to 0) | 0.1 (0 to 0.3) | 0 (0 to 0) | 0 (0 to 0) | -6.1 (-6.65 to -5.55) |
| Comoros | 0 (0 to 0) | 0.2 (0 to 0.6) | 0 (0 to 0) | 0.1 (0 to 0.3) | -3.06 (-3.26 to -2.86) |
| Congo | 0 (0 to 0) | 0.1 (0 to 0.5) | 0 (0 to 0) | 0.1 (0 to 0.2) | -2.43 (-2.73 to -2.13) |
| Cook Islands | 0 (0 to 0) | 0.1 (0 to 0.4) | 0 (0 to 0) | 0.1 (0 to 0.2) | -2.78 (-3.22 to -2.33) |
| Costa Rica | 0 (0 to 0) | 0.1 (0 to 0.2) | 0 (0 to 0) | 0 (0 to 0.1) | -2.98 (-3.44 to -2.52) |
| Croatia | 0 (0 to 0.1) | 0.4 (0.1 to 0.8) | 0 (0 to 0) | 0.1 (0 to 0.2) | -4.73 (-6.22 to -3.22) |
| Cuba | 0 (0 to 0) | 0 (0 to 0.1) | 0 (0 to 0) | 0 (0 to 0.1) | -2.35 (-2.84 to -1.86) |
| Cyprus | 0 (0 to 0) | 0.1 (0 to 0.5) | 0 (0 to 0) | 0 (0 to 0.2) | -3.54 (-3.96 to -3.11) |
| Czechia | 0.1 (0 to 0.1) | 0.5 (0.2 to 1.1) | 0 (0 to 0.1) | 0.1 (0 to 0.2) | -5.72 (-6.08 to -5.37) |
| Côte d'Ivoire | 0 (0 to 0) | 0.1 (0 to 0.5) | 0 (0 to 0) | 0.1 (0 to 0.2) | -2.86 (-3.09 to -2.62) |
| Democratic People's Republic of Korea | 0.2 (0.1 to 0.3) | 1 (0.3 to 2.2) | 0.2 (0 to 0.4) | 0.5 (0.1 to 1.3) | -2.44 (-2.64 to -2.23) |
| Democratic Republic of the Congo | 0 (0 to 0.1) | 0.1 (0 to 0.3) | 0 (0 to 0.1) | 0 (0 to 0.2) | -1.66 (-1.76 to -1.56) |
| Denmark | 0 (0 to 0) | 0.1 (0 to 0.2) | 0 (0 to 0) | 0 (0 to 0) | -5.33 (-6.27 to -4.38) |
| Djibouti | 0 (0 to 0) | 0.2 (0 to 0.5) | 0 (0 to 0) | 0.1 (0 to 0.2) | -3.17 (-3.34 to -3.01) |
| Dominica | 0 (0 to 0) | 0.1 (0 to 0.2) | 0 (0 to 0) | 0 (0 to 0.1) | -2.51 (-2.62 to -2.4) |
| Dominican Republic | 0 (0 to 0) | 0 (0 to 0.1) | 0 (0 to 0) | 0 (0 to 0.1) | -1.86 (-2.49 to -1.23) |
| Ecuador | 0 (0 to 0) | 0.1 (0 to 0.3) | 0 (0 to 0) | 0 (0 to 0.1) | -4.09 (-4.58 to -3.6) |
| Egypt | 0 (0 to 0.1) | 0 (0 to 0.2) | 0 (0 to 0) | 0 (0 to 0.1) | -2.96 (-3.25 to -2.67) |
| El Salvador | 0 (0 to 0) | 0 (0 to 0.1) | 0 (0 to 0) | 0 (0 to 0.1) | -2.76 (-3.29 to -2.24) |
| Equatorial Guinea | 0 (0 to 0) | 0.1 (0 to 0.6) | 0 (0 to 0) | 0 (0 to 0.1) | -4.73 (-4.9 to -4.55) |
| Eritrea | 0 (0 to 0) | 0.3 (0 to 1) | 0 (0 to 0) | 0.1 (0 to 0.4) | -2.91 (-2.99 to -2.83) |
| Estonia | 0 (0 to 0) | 0.1 (0 to 0.2) | 0 (0 to 0) | 0 (0 to 0) | -5.95 (-6.56 to -5.33) |
| Eswatini | 0 (0 to 0) | 0.1 (0 to 0.5) | 0 (0 to 0) | 0.1 (0 to 0.2) | -2.93 (-3.14 to -2.71) |
| Ethiopia | 0.1 (0 to 0.2) | 0.3 (0 to 0.9) | 0 (0 to 0.1) | 0.1 (0 to 0.3) | -4.35 (-4.46 to -4.25) |
| Fiji | 0 (0 to 0) | 0.4 (0.1 to 1.2) | 0 (0 to 0) | 0.3 (0 to 0.7) | -1.81 (-2.16 to -1.46) |
| Finland | 0 (0 to 0) | 0 (0 to 0.1) | 0 (0 to 0) | 0 (0 to 0) | -5.64 (-5.94 to -5.34) |
| France | 0 (0 to 0.1) | 0 (0 to 0.1) | 0 (0 to 0.2) | 0 (0 to 0.1) | -1.7 (-2.04 to -1.36) |
| Gabon | 0 (0 to 0) | 0.1 (0 to 0.3) | 0 (0 to 0) | 0 (0 to 0.2) | -2.51 (-2.66 to -2.37) |
| Gambia | 0 (0 to 0) | 0.1 (0 to 0.4) | 0 (0 to 0) | 0.1 (0 to 0.2) | -1.84 (-2.57 to -1.1) |
| Georgia | 0 (0 to 0) | 0.2 (0 to 0.4) | 0 (0 to 0) | 0.2 (0 to 0.5) | 0.24 (-0.23 to 0.71) |
| Germany | 0.1 (0 to 0.3) | 0.1 (0 to 0.2) | 0.1 (0 to 0.3) | 0 (0 to 0.1) | -2.12 (-2.57 to -1.66) |
| Ghana | 0 (0 to 0) | 0.1 (0 to 0.4) | 0 (0 to 0) | 0.1 (0 to 0.2) | -1.57 (-1.77 to -1.36) |
| Greece | 0 (0 to 0) | 0 (0 to 0.1) | 0 (0 to 0) | 0 (0 to 0) | -3.08 (-3.8 to -2.34) |
| Greenland | 0 (0 to 0) | 0.1 (0 to 0.3) | 0 (0 to 0) | 0 (0 to 0.1) | -3.98 (-4.44 to -3.51) |
| Grenada | 0 (0 to 0) | 0.1 (0 to 0.4) | 0 (0 to 0) | 0 (0 to 0.1) | -3.26 (-3.43 to -3.09) |
| Guam | 0 (0 to 0) | 0.2 (0 to 0.4) | 0 (0 to 0) | 0.1 (0 to 0.2) | -3.59 (-4.04 to -3.14) |
| Guatemala | 0 (0 to 0) | 0 (0 to 0.1) | 0 (0 to 0) | 0 (0 to 0) | -3.32 (-4.1 to -2.54) |
| Guinea | 0 (0 to 0) | 0.2 (0 to 0.7) | 0 (0 to 0) | 0.1 (0 to 0.3) | -2.34 (-2.45 to -2.23) |
| Guinea-Bissau | 0 (0 to 0) | 0.2 (0 to 0.9) | 0 (0 to 0) | 0.1 (0 to 0.4) | -2.8 (-2.88 to -2.71) |
| Guyana | 0 (0 to 0) | 0.1 (0 to 0.3) | 0 (0 to 0) | 0 (0 to 0.1) | -2.55 (-2.97 to -2.14) |
| Haiti | 0 (0 to 0) | 0.3 (0 to 1) | 0 (0 to 0) | 0.1 (0 to 0.5) | -2.47 (-2.63 to -2.32) |
| Honduras | 0 (0 to 0) | 0 (0 to 0.1) | 0 (0 to 0) | 0 (0 to 0.1) | -0.98 (-1.55 to -0.41) |
| Hungary | 0.1 (0 to 0.2) | 0.8 (0.4 to 1.5) | 0 (0 to 0) | 0.1 (0 to 0.2) | -6.44 (-6.77 to -6.11) |
| Iceland | 0 (0 to 0) | 0 (0 to 0.1) | 0 (0 to 0) | 0 (0 to 0) | -2.31 (-2.51 to -2.11) |
| India | 2.8 (0.3 to 8.3) | 0.6 (0.1 to 1.8) | 3.6 (0.4 to 10.3) | 0.3 (0 to 0.9) | -2.15 (-2.66 to -1.63) |
| Indonesia | 0.1 (0 to 0.3) | 0.1 (0 to 0.3) | 0.1 (0 to 0.1) | 0 (0 to 0.1) | -4.97 (-5.17 to -4.76) |
| Iran (Islamic Republic of) | 0 (0 to 0) | 0 (0 to 0.1) | 0 (0 to 0) | 0 (0 to 0) | -2.93 (-3.3 to -2.55) |
| Iraq | 0 (0 to 0) | 0.1 (0 to 0.2) | 0 (0 to 0) | 0 (0 to 0.1) | -4.31 (-4.74 to -3.88) |
| Ireland | 0 (0 to 0) | 0 (0 to 0.1) | 0 (0 to 0) | 0 (0 to 0) | -3.47 (-3.67 to -3.27) |
| Israel | 0 (0 to 0) | 0 (0 to 0.2) | 0 (0 to 0) | 0 (0 to 0.1) | -1.41 (-1.6 to -1.22) |
| Italy | 0.1 (0 to 0.2) | 0.1 (0 to 0.3) | 0.1 (0 to 0.2) | 0 (0 to 0.1) | -3.3 (-3.46 to -3.15) |
| Jamaica | 0 (0 to 0) | 0 (0 to 0.1) | 0 (0 to 0) | 0 (0 to 0.1) | -3.01 (-3.85 to -2.18) |
| Japan | 0.2 (0.1 to 0.5) | 0.1 (0 to 0.3) | 0.2 (0 to 0.5) | 0 (0 to 0.1) | -4.8 (-5.11 to -4.5) |
| Jordan | 0 (0 to 0) | 0 (0 to 0.1) | 0 (0 to 0) | 0 (0 to 0) | -4.19 (-4.48 to -3.89) |
| Kazakhstan | 0.1 (0 to 0.1) | 0.3 (0.1 to 0.8) | 0 (0 to 0) | 0.1 (0 to 0.2) | -6.09 (-6.54 to -5.63) |
| Kenya | 0 (0 to 0) | 0.1 (0 to 0.3) | 0 (0 to 0) | 0.1 (0 to 0.2) | -1.86 (-1.93 to -1.8) |
| Kiribati | 0 (0 to 0) | 0.7 (0.1 to 2) | 0 (0 to 0) | 0.5 (0 to 1.3) | -1.53 (-1.62 to -1.45) |
| Kuwait | 0 (0 to 0) | 0 (0 to 0.1) | 0 (0 to 0) | 0 (0 to 0) | -4.69 (-5.63 to -3.74) |
| Kyrgyzstan | 0 (0 to 0) | 0.4 (0.1 to 0.9) | 0 (0 to 0) | 0.1 (0 to 0.3) | -5.21 (-5.68 to -4.73) |
| Lao People's Democratic Republic | 0 (0 to 0) | 0.4 (0.1 to 1) | 0 (0 to 0) | 0.1 (0 to 0.4) | -3.9 (-3.99 to -3.8) |
| Latvia | 0 (0 to 0) | 0.2 (0 to 0.5) | 0 (0 to 0) | 0 (0 to 0.1) | -6.2 (-8.14 to -4.23) |
| Lebanon | 0 (0 to 0) | 0 (0 to 0.1) | 0 (0 to 0) | 0 (0 to 0) | -3.72 (-3.81 to -3.63) |
| Lesotho | 0 (0 to 0) | 0.1 (0 to 0.6) | 0 (0 to 0) | 0.1 (0 to 0.4) | -1.75 (-1.92 to -1.58) |
| Liberia | 0 (0 to 0) | 0.1 (0 to 0.5) | 0 (0 to 0) | 0.1 (0 to 0.2) | -2.69 (-2.87 to -2.51) |
| Libya | 0 (0 to 0) | 0 (0 to 0.1) | 0 (0 to 0) | 0 (0 to 0) | -2.3 (-2.65 to -1.96) |
| Lithuania | 0 (0 to 0) | 0.2 (0 to 0.6) | 0 (0 to 0) | 0 (0 to 0.1) | -5.92 (-6.65 to -5.18) |
| Luxembourg | 0 (0 to 0) | 0 (0 to 0.1) | 0 (0 to 0) | 0 (0 to 0.1) | -2.46 (-2.61 to -2.31) |
| Madagascar | 0 (0 to 0) | 0.3 (0 to 0.9) | 0 (0 to 0.1) | 0.2 (0 to 0.5) | -2.21 (-2.38 to -2.04) |
| Malawi | 0 (0 to 0) | 0.2 (0 to 0.5) | 0 (0 to 0) | 0.1 (0 to 0.3) | -2.89 (-2.97 to -2.81) |
| Malaysia | 0 (0 to 0.1) | 0.3 (0.1 to 0.6) | 0 (0 to 0) | 0 (0 to 0.1) | -6.33 (-6.88 to -5.79) |
| Maldives | 0 (0 to 0) | 0.3 (0.1 to 0.8) | 0 (0 to 0) | 0.1 (0 to 0.1) | -6.03 (-6.23 to -5.82) |
| Mali | 0 (0 to 0) | 0.2 (0 to 0.7) | 0 (0 to 0) | 0.1 (0 to 0.3) | -2.87 (-3.08 to -2.65) |
| Malta | 0 (0 to 0) | 0.1 (0 to 0.2) | 0 (0 to 0) | 0 (0 to 0.1) | -2.5 (-2.8 to -2.21) |
| Marshall Islands | 0 (0 to 0) | 0.6 (0.1 to 1.9) | 0 (0 to 0) | 0.4 (0 to 1.2) | -1.8 (-2.04 to -1.57) |
| Mauritania | 0 (0 to 0) | 0.1 (0 to 0.5) | 0 (0 to 0) | 0 (0 to 0.1) | -4.07 (-4.2 to -3.94) |
| Mauritius | 0 (0 to 0) | 0.2 (0 to 0.5) | 0 (0 to 0) | 0 (0 to 0.1) | -5.93 (-6.54 to -5.31) |
| Mexico | 0 (0 to 0.1) | 0.1 (0 to 0.3) | 0 (0 to 0.1) | 0 (0 to 0.1) | -4.87 (-5.04 to -4.71) |
| Micronesia (Federated States of) | 0 (0 to 0) | 0.7 (0.1 to 2) | 0 (0 to 0) | 0.4 (0 to 1.1) | -2.13 (-2.21 to -2.05) |
| Monaco | 0 (0 to 0) | 0 (0 to 0.1) | 0 (0 to 0) | 0 (0 to 0) | -1.43 (-1.53 to -1.34) |
| Mongolia | 0 (0 to 0) | 0.6 (0.1 to 1.7) | 0 (0 to 0) | 0.2 (0 to 0.5) | -4.27 (-4.5 to -4.05) |
| Montenegro | 0 (0 to 0) | 0.2 (0.1 to 0.4) | 0 (0 to 0) | 0.1 (0 to 0.2) | -2.17 (-2.44 to -1.89) |
| Morocco | 0 (0 to 0) | 0 (0 to 0.2) | 0 (0 to 0) | 0 (0 to 0.1) | -2.83 (-3.15 to -2.51) |
| Mozambique | 0 (0 to 0) | 0.2 (0 to 0.6) | 0 (0 to 0) | 0.1 (0 to 0.3) | -2.32 (-2.4 to -2.24) |
| Myanmar | 0.1 (0 to 0.2) | 0.4 (0.1 to 0.8) | 0.1 (0 to 0.1) | 0.1 (0 to 0.3) | -4.18 (-4.31 to -4.05) |
| Namibia | 0 (0 to 0) | 0.1 (0 to 0.5) | 0 (0 to 0) | 0 (0 to 0.2) | -4.05 (-4.2 to -3.9) |
| Nauru | 0 (0 to 0) | 0.4 (0.1 to 1.1) | 0 (0 to 0) | 0.3 (0 to 0.8) | -1.29 (-1.46 to -1.11) |
| Nepal | 0 (0 to 0.2) | 0.5 (0 to 1.7) | 0.1 (0 to 0.2) | 0.3 (0 to 1.1) | -0.83 (-0.99 to -0.67) |
| Netherlands | 0 (0 to 0) | 0 (0 to 0.1) | 0 (0 to 0) | 0 (0 to 0.1) | -0.45 (-1.32 to 0.44) |
| New Zealand | 0 (0 to 0) | 0.1 (0 to 0.3) | 0 (0 to 0) | 0 (0 to 0.1) | -2.66 (-3.13 to -2.18) |
| Nicaragua | 0 (0 to 0) | 0.1 (0 to 0.2) | 0 (0 to 0) | 0 (0 to 0.1) | -3.61 (-4.09 to -3.13) |
| Niger | 0 (0 to 0) | 0.2 (0 to 0.7) | 0 (0 to 0) | 0.1 (0 to 0.4) | -2.33 (-2.55 to -2.11) |
| Nigeria | 0.1 (0 to 0.2) | 0.1 (0 to 0.5) | 0 (0 to 0.1) | 0 (0 to 0.2) | -3.76 (-3.98 to -3.53) |
| Niue | 0 (0 to 0) | 0.3 (0 to 0.7) | 0 (0 to 0) | 0.1 (0 to 0.4) | -2.23 (-2.3 to -2.16) |
| North Macedonia | 0 (0 to 0) | 0.4 (0.1 to 0.8) | 0 (0 to 0) | 0.1 (0 to 0.2) | -4.81 (-5 to -4.62) |
| Northern Mariana Islands | 0 (0 to 0) | 0.1 (0 to 0.4) | 0 (0 to 0) | 0.1 (0 to 0.2) | -1.51 (-1.63 to -1.39) |
| Norway | 0 (0 to 0) | 0 (0 to 0.1) | 0 (0 to 0) | 0 (0 to 0) | -3.36 (-3.76 to -2.97) |
| Oman | 0 (0 to 0) | 0 (0 to 0.1) | 0 (0 to 0) | 0 (0 to 0) | -4.07 (-4.42 to -3.72) |
| Pakistan | 0.3 (0 to 0.9) | 0.5 (0 to 1.6) | 0.5 (0 to 1.6) | 0.5 (0 to 1.4) | 0 (-0.12 to 0.11) |
| Palau | 0 (0 to 0) | 0.2 (0 to 0.5) | 0 (0 to 0) | 0.1 (0 to 0.3) | -2.02 (-2.15 to -1.89) |
| Palestine | 0 (0 to 0) | 0 (0 to 0.1) | 0 (0 to 0) | 0 (0 to 0) | -3.74 (-3.84 to -3.64) |
| Panama | 0 (0 to 0) | 0.1 (0 to 0.2) | 0 (0 to 0) | 0 (0 to 0.1) | -4.22 (-4.62 to -3.82) |
| Papua New Guinea | 0 (0 to 0) | 0.6 (0.1 to 1.9) | 0 (0 to 0.1) | 0.5 (0 to 1.5) | -0.76 (-0.83 to -0.68) |
| Paraguay | 0 (0 to 0) | 0 (0 to 0.1) | 0 (0 to 0) | 0 (0 to 0.1) | -2.09 (-2.63 to -1.55) |
| Peru | 0 (0 to 0) | 0.1 (0 to 0.2) | 0 (0 to 0) | 0 (0 to 0.1) | -3.92 (-4.7 to -3.12) |
| Philippines | 0 (0 to 0.1) | 0.1 (0 to 0.2) | 0.1 (0 to 0.2) | 0.1 (0 to 0.2) | -0.69 (-1.2 to -0.18) |
| Poland | 0.2 (0 to 0.4) | 0.4 (0.1 to 0.9) | 0.1 (0 to 0.1) | 0.1 (0 to 0.2) | -5.74 (-6.22 to -5.26) |
| Portugal | 0 (0 to 0) | 0.1 (0 to 0.2) | 0 (0 to 0) | 0 (0 to 0.1) | -3.24 (-3.88 to -2.59) |
| Puerto Rico | 0 (0 to 0) | 0 (0 to 0.1) | 0 (0 to 0) | 0 (0 to 0) | -3.72 (-4.21 to -3.23) |
| Qatar | 0 (0 to 0) | 0 (0 to 0.1) | 0 (0 to 0) | 0 (0 to 0) | -4.35 (-4.81 to -3.89) |
| Republic of Korea | 0 (0 to 0) | 0.1 (0 to 0.2) | 0 (0 to 0) | 0 (0 to 0.1) | -3.91 (-4.1 to -3.72) |
| Republic of Moldova | 0 (0 to 0) | 0.1 (0 to 0.4) | 0 (0 to 0) | 0 (0 to 0.1) | -5.45 (-6.78 to -4.1) |
| Romania | 0.2 (0.1 to 0.3) | 0.6 (0.2 to 1.1) | 0 (0 to 0.1) | 0.1 (0 to 0.2) | -6.16 (-6.49 to -5.83) |
| Russian Federation | 0.4 (0 to 1) | 0.2 (0 to 0.6) | 0.1 (0 to 0.3) | 0 (0 to 0.1) | -5.44 (-6.46 to -4.42) |
| Rwanda | 0 (0 to 0) | 0.4 (0 to 1) | 0 (0 to 0) | 0.1 (0 to 0.3) | -4.67 (-4.84 to -4.5) |
| Saint Kitts and Nevis | 0 (0 to 0) | 0.1 (0 to 0.2) | 0 (0 to 0) | 0 (0 to 0.1) | -4.38 (-4.76 to -4) |
| Saint Lucia | 0 (0 to 0) | 0.1 (0 to 0.3) | 0 (0 to 0) | 0 (0 to 0.1) | -3.74 (-3.94 to -3.54) |
| Saint Vincent and the Grenadines | 0 (0 to 0) | 0.1 (0 to 0.2) | 0 (0 to 0) | 0 (0 to 0.1) | -2.32 (-2.67 to -1.97) |
| Samoa | 0 (0 to 0) | 0.1 (0 to 0.5) | 0 (0 to 0) | 0.1 (0 to 0.3) | -0.93 (-1 to -0.85) |
| San Marino | 0 (0 to 0) | 0.1 (0 to 0.2) | 0 (0 to 0) | 0 (0 to 0.1) | -1.1 (-1.25 to -0.95) |
| Sao Tome and Principe | 0 (0 to 0) | 0.2 (0 to 0.7) | 0 (0 to 0) | 0.1 (0 to 0.4) | -1.49 (-1.6 to -1.39) |
| Saudi Arabia | 0 (0 to 0) | 0 (0 to 0.1) | 0 (0 to 0) | 0 (0 to 0) | -3.91 (-4.17 to -3.65) |
| Senegal | 0 (0 to 0) | 0.1 (0 to 0.4) | 0 (0 to 0) | 0.1 (0 to 0.2) | -2.49 (-2.72 to -2.26) |
| Serbia | 0 (0 to 0.1) | 0.3 (0.1 to 0.6) | 0 (0 to 0) | 0.1 (0 to 0.2) | -4.09 (-4.48 to -3.69) |
| Seychelles | 0 (0 to 0) | 0.3 (0.1 to 0.6) | 0 (0 to 0) | 0 (0 to 0.1) | -7.07 (-7.53 to -6.61) |
| Sierra Leone | 0 (0 to 0) | 0.1 (0 to 0.5) | 0 (0 to 0) | 0.1 (0 to 0.3) | -2.24 (-2.4 to -2.07) |
| Singapore | 0 (0 to 0) | 0.1 (0 to 0.3) | 0 (0 to 0) | 0 (0 to 0) | -7.05 (-7.36 to -6.73) |
| Slovakia | 0 (0 to 0) | 0.2 (0.1 to 0.4) | 0 (0 to 0) | 0.1 (0 to 0.2) | -3.6 (-3.85 to -3.34) |
| Slovenia | 0 (0 to 0) | 0.4 (0.2 to 0.9) | 0 (0 to 0) | 0.2 (0 to 0.4) | -3.45 (-3.53 to -3.37) |
| Solomon Islands | 0 (0 to 0) | 0.8 (0.1 to 2.3) | 0 (0 to 0) | 0.5 (0.1 to 1.6) | -1.34 (-1.51 to -1.16) |
| Somalia | 0 (0 to 0) | 0.4 (0 to 1.1) | 0 (0 to 0) | 0.2 (0 to 0.7) | -2.26 (-2.35 to -2.18) |
| South Africa | 0 (0 to 0.1) | 0.1 (0 to 0.3) | 0 (0 to 0) | 0 (0 to 0.1) | -3.9 (-4.39 to -3.4) |
| South Sudan | 0 (0 to 0) | 0.2 (0 to 0.6) | 0 (0 to 0) | 0.1 (0 to 0.3) | -2.78 (-2.84 to -2.72) |
| Spain | 0 (0 to 0.1) | 0.1 (0 to 0.2) | 0 (0 to 0.1) | 0 (0 to 0.1) | -2.83 (-3.04 to -2.63) |
| Sri Lanka | 0 (0 to 0) | 0.2 (0 to 0.4) | 0 (0 to 0) | 0 (0 to 0.1) | -5.43 (-6.04 to -4.82) |
| Sudan | 0 (0 to 0) | 0.1 (0 to 0.3) | 0 (0 to 0) | 0 (0 to 0.1) | -3.13 (-3.26 to -2.99) |
| Suriname | 0 (0 to 0) | 0 (0 to 0.1) | 0 (0 to 0) | 0 (0 to 0.1) | -2.77 (-3.59 to -1.95) |
| Sweden | 0 (0 to 0) | 0 (0 to 0.1) | 0 (0 to 0) | 0 (0 to 0) | -3.15 (-3.28 to -3.03) |
| Switzerland | 0 (0 to 0) | 0.1 (0 to 0.2) | 0 (0 to 0) | 0 (0 to 0) | -5.14 (-5.33 to -4.95) |
| Syrian Arab Republic | 0 (0 to 0) | 0.1 (0 to 0.4) | 0 (0 to 0) | 0 (0 to 0.1) | -6.22 (-6.62 to -5.81) |
| Taiwan (Province of China) | 0 (0 to 0.1) | 0.2 (0 to 0.6) | 0 (0 to 0) | 0 (0 to 0.1) | -7.22 (-7.57 to -6.86) |
| Tajikistan | 0 (0 to 0) | 0.4 (0.1 to 0.9) | 0 (0 to 0) | 0.1 (0 to 0.3) | -4.19 (-4.59 to -3.78) |
| Thailand | 0.1 (0 to 0.2) | 0.3 (0.1 to 0.7) | 0 (0 to 0.1) | 0 (0 to 0.1) | -8.74 (-9.16 to -8.33) |
| Timor-Leste | 0 (0 to 0) | 0.3 (0.1 to 0.7) | 0 (0 to 0) | 0.1 (0 to 0.4) | -2.81 (-3.06 to -2.56) |
| Togo | 0 (0 to 0) | 0.1 (0 to 0.5) | 0 (0 to 0) | 0.1 (0 to 0.2) | -2.5 (-2.7 to -2.29) |
| Tokelau | 0 (0 to 0) | 0.4 (0 to 1.1) | 0 (0 to 0) | 0.2 (0 to 0.5) | -2.75 (-2.78 to -2.71) |
| Tonga | 0 (0 to 0) | 0.2 (0 to 0.4) | 0 (0 to 0) | 0.1 (0 to 0.2) | -1.75 (-1.97 to -1.54) |
| Trinidad and Tobago | 0 (0 to 0) | 0 (0 to 0.2) | 0 (0 to 0) | 0 (0 to 0.1) | -3.79 (-4.37 to -3.21) |
| Tunisia | 0 (0 to 0) | 0 (0 to 0.1) | 0 (0 to 0) | 0 (0 to 0) | -3.03 (-3.1 to -2.96) |
| Turkey | NA | NA | NA | NA | NA |
| Turkmenistan | 0 (0 to 0) | 0.2 (0 to 0.6) | 0 (0 to 0) | 0.1 (0 to 0.2) | -4.33 (-5.75 to -2.9) |
| Tuvalu | 0 (0 to 0) | 0.6 (0.1 to 1.6) | 0 (0 to 0) | 0.3 (0 to 0.9) | -2.34 (-2.44 to -2.23) |
| Uganda | 0 (0 to 0) | 0.2 (0 to 0.6) | 0 (0 to 0) | 0.1 (0 to 0.2) | -3.23 (-3.33 to -3.13) |
| Ukraine | 0 (0 to 0.1) | 0 (0 to 0.1) | 0 (0 to 0.1) | 0 (0 to 0.1) | -1.49 (-2.64 to -0.33) |
| United Arab Emirates | 0 (0 to 0) | 0.1 (0 to 0.5) | 0 (0 to 0) | 0 (0 to 0.2) | -3.3 (-3.57 to -3.03) |
| United Kingdom | 0 (0 to 0.2) | 0 (0 to 0.2) | 0 (0 to 0.1) | 0 (0 to 0.1) | -3.99 (-4.21 to -3.78) |
| United Republic of Tanzania | 0 (0 to 0.1) | 0.2 (0 to 0.5) | 0 (0 to 0.1) | 0.1 (0 to 0.3) | -2.45 (-2.58 to -2.32) |
| United States of America | 0.1 (0 to 0.5) | 0 (0 to 0.1) | 0.1 (0 to 0.4) | 0 (0 to 0.1) | -2.34 (-2.59 to -2.09) |
| United States Virgin Islands | 0 (0 to 0) | 0 (0 to 0.1) | 0 (0 to 0) | 0 (0 to 0) | -2.12 (-2.34 to -1.9) |
| Uruguay | 0 (0 to 0) | 0.1 (0 to 0.2) | 0 (0 to 0) | 0 (0 to 0.1) | -2.35 (-2.73 to -1.98) |
| Uzbekistan | 0 (0 to 0.1) | 0.3 (0.1 to 0.8) | 0 (0 to 0.1) | 0.2 (0 to 0.5) | -2.42 (-2.95 to -1.88) |
| Vanuatu | 0 (0 to 0) | 0.6 (0.1 to 1.8) | 0 (0 to 0) | 0.5 (0 to 1.4) | -1.01 (-1.14 to -0.87) |
| Venezuela (Bolivarian Republic of) | 0 (0 to 0) | 0.1 (0 to 0.2) | 0 (0 to 0) | 0 (0 to 0.1) | -3.85 (-4.1 to -3.59) |
| Viet Nam | 0.1 (0 to 0.3) | 0.3 (0.1 to 0.7) | 0.1 (0 to 0.2) | 0.1 (0 to 0.2) | -4.44 (-4.52 to -4.36) |
| Yemen | 0 (0 to 0) | 0.1 (0 to 0.3) | 0 (0 to 0) | 0 (0 to 0.1) | -2.2 (-2.44 to -1.97) |
| Zambia | 0 (0 to 0) | 0.2 (0 to 0.6) | 0 (0 to 0) | 0.1 (0 to 0.3) | -2.36 (-2.58 to -2.15) |
| Zimbabwe | 0 (0 to 0) | 0.2 (0 to 0.5) | 0 (0 to 0) | 0.1 (0 to 0.5) | -0.17 (-0.32 to -0.02) |
