# Supplementary material for: Spatiotemporal patterns of rheumatic heart disease burden attributable to high systolic blood pressure, high sodium diet, and lead exposure (1990 to 2019): a longitudinal observational study
Source: Front Nutr. 2024 Sep 26;11:1419349. doi: 10.3389/fnut.2024.1419349 (PMC11466049; doi:10.3389/fnut.2024.1419349)
Supplement: Supplementary file 4 [file Table_4.docx]

**Supplementary table 4. Disability-adjusted life year of rheumatic heart disease due to diet high in sodium**

| **location** | **1990 Counts**  **(thousand)** | **Age-standardised rate (per 100 000 population), 1990** | **2019 Counts**  **(thousand)** | **Age-standardised rate (per 100 000 population), 2019** | **Average annual percent change** |
| --- | --- | --- | --- | --- | --- |
| Afghanistan | 0.3 (0 to 1) | 3.5 (0.6 to 14.3) | 0.4 (0.1 to 1.6) | 2 (0.4 to 7.6) | -1.9 (-2.04 to -1.76) |
| Albania | 0.3 (0.1 to 0.6) | 13.8 (5.2 to 26.3) | 0.1 (0 to 0.3) | 3.4 (0.9 to 7.3) | -4.81 (-5.05 to -4.57) |
| Algeria | 0.2 (0 to 0.8) | 1.2 (0.2 to 4.6) | 0.2 (0 to 0.8) | 0.5 (0.1 to 1.8) | -2.94 (-2.99 to -2.89) |
| American Samoa | 0 (0 to 0) | 3 (0.5 to 11.8) | 0 (0 to 0) | 2.3 (0.3 to 8.9) | -1 (-1.26 to -0.73) |
| Andorra | 0 (0 to 0) | 0.5 (0.1 to 1.7) | 0 (0 to 0) | 0.3 (0 to 1) | -1.8 (-1.99 to -1.61) |
| Angola | 0.3 (0 to 1.2) | 4.5 (0.4 to 18.3) | 0.5 (0 to 2) | 2.7 (0.2 to 10.8) | -1.71 (-1.9 to -1.53) |
| Antigua and Barbuda | 0 (0 to 0) | 1.5 (0.2 to 5.7) | 0 (0 to 0) | 0.9 (0.1 to 3.4) | -1.7 (-1.97 to -1.43) |
| Argentina | 1.3 (0.1 to 3.9) | 4.2 (0.4 to 12.3) | 1 (0.1 to 3) | 1.9 (0.2 to 5.7) | -2.72 (-2.98 to -2.45) |
| Armenia | 0.4 (0.1 to 0.9) | 11.3 (2 to 27.2) | 0.1 (0 to 0.3) | 2.7 (0.3 to 8.4) | -4.86 (-5.35 to -4.37) |
| Australia | 0.1 (0 to 0.5) | 0.7 (0.1 to 2.4) | 0.1 (0 to 0.4) | 0.3 (0 to 1.1) | -2.74 (-3.16 to -2.33) |
| Austria | 0.2 (0 to 0.6) | 1.5 (0.1 to 4.8) | 0.1 (0 to 0.4) | 0.7 (0.1 to 1.9) | -2.86 (-3.06 to -2.66) |
| Azerbaijan | 0.4 (0.1 to 1) | 6.7 (1.2 to 16.3) | 0.3 (0 to 0.8) | 2.3 (0.2 to 6.6) | -3.63 (-3.93 to -3.33) |
| Bahamas | 0 (0 to 0) | 1.4 (0.2 to 5.2) | 0 (0 to 0) | 1 (0.1 to 3.7) | -1.27 (-1.57 to -0.98) |
| Bahrain | 0 (0 to 0) | 0.5 (0.1 to 1.9) | 0 (0 to 0) | 0.2 (0.1 to 0.8) | -3 (-3.32 to -2.67) |
| Bangladesh | 1.9 (0.2 to 7) | 2.7 (0.3 to 9.7) | 3.3 (0.3 to 9.8) | 2.2 (0.2 to 6.5) | -0.55 (-1.25 to 0.16) |
| Barbados | 0 (0 to 0) | 1.2 (0.2 to 4.9) | 0 (0 to 0) | 0.8 (0.1 to 3.1) | -1.34 (-1.51 to -1.17) |
| Belarus | 0.6 (0.1 to 2) | 4.9 (0.5 to 15.8) | 0.2 (0 to 0.5) | 1.1 (0.1 to 3.7) | -5.03 (-5.76 to -4.29) |
| Belgium | 0.1 (0 to 0.3) | 0.6 (0.1 to 1.7) | 0.2 (0 to 0.5) | 0.6 (0.1 to 2) | 0.3 (-0.06 to 0.67) |
| Belize | 0 (0 to 0) | 1.3 (0.2 to 5) | 0 (0 to 0) | 1 (0.1 to 3.7) | -0.97 (-1.25 to -0.69) |
| Benin | 0.1 (0 to 0.5) | 5.2 (0.3 to 18.8) | 0.2 (0 to 0.7) | 2.6 (0.2 to 10.1) | -2.31 (-2.44 to -2.18) |
| Bermuda | 0 (0 to 0) | 0.9 (0.1 to 3.1) | 0 (0 to 0) | 0.2 (0 to 0.9) | -4.18 (-4.36 to -4) |
| Bhutan | 0 (0 to 0.2) | 13.1 (1.1 to 48.8) | 0.1 (0 to 0.2) | 8.5 (0.6 to 29.4) | -1.47 (-1.54 to -1.39) |
| Bolivia (Plurinational State of) | 0.2 (0 to 0.7) | 5.7 (0.4 to 18) | 0.2 (0 to 0.7) | 2.3 (0.2 to 7.1) | -3.09 (-3.21 to -2.98) |
| Bosnia and Herzegovina | 0.2 (0.1 to 0.5) | 5.7 (2 to 10.9) | 0.1 (0 to 0.1) | 1.1 (0.3 to 2.4) | -5.64 (-6 to -5.28) |
| Botswana | 0.1 (0 to 0.2) | 6.4 (0.3 to 24) | 0.1 (0 to 0.2) | 2.6 (0.2 to 10.2) | -3.01 (-3.14 to -2.88) |
| Brazil | 3.9 (0.3 to 11.6) | 3.3 (0.3 to 9.5) | 4.3 (0.4 to 13) | 1.7 (0.2 to 5.3) | -2.11 (-2.24 to -1.97) |
| Brunei Darussalam | 0 (0 to 0) | 6.4 (1.2 to 14.6) | 0 (0 to 0) | 2.9 (0.4 to 6.7) | -2.79 (-2.93 to -2.64) |
| Bulgaria | 2.4 (0.8 to 4.4) | 19.8 (7.2 to 37.3) | 0.4 (0.1 to 1) | 3.8 (0.9 to 8.7) | -5.49 (-5.88 to -5.11) |
| Burkina Faso | 0.1 (0 to 0.5) | 2.5 (0.2 to 10.3) | 0.2 (0 to 1) | 2 (0.2 to 8.1) | -0.7 (-0.79 to -0.62) |
| Burundi | 0.3 (0 to 0.9) | 9 (0.6 to 27.5) | 0.2 (0 to 0.8) | 3.5 (0.2 to 12.1) | -3.22 (-3.35 to -3.09) |
| Cabo Verde | 0 (0 to 0) | 4.7 (0.4 to 18.7) | 0 (0 to 0) | 1.5 (0.1 to 6.1) | -3.9 (-4.17 to -3.64) |
| Cambodia | 0.9 (0.2 to 2.1) | 14.4 (3 to 32.9) | 0.6 (0.1 to 1.5) | 3.8 (0.4 to 9.9) | -4.47 (-4.6 to -4.34) |
| Cameroon | 0.2 (0 to 0.8) | 3.6 (0.3 to 14.9) | 0.3 (0 to 1.5) | 2 (0.1 to 8.2) | -2.1 (-2.16 to -2.04) |
| Canada | 0.4 (0 to 1.1) | 1.1 (0.1 to 3.5) | 0.4 (0 to 1.2) | 0.6 (0.1 to 1.8) | -2.28 (-2.4 to -2.15) |
| Central African Republic | 0.1 (0 to 0.4) | 6 (0.5 to 25.3) | 0.2 (0 to 0.7) | 5 (0.3 to 21.4) | -0.58 (-0.68 to -0.49) |
| Chad | 0.1 (0 to 0.6) | 4.5 (0.3 to 18.8) | 0.2 (0 to 0.9) | 2.8 (0.2 to 11.2) | -1.56 (-1.68 to -1.43) |
| Chile | 0.5 (0 to 1.3) | 4.4 (0.4 to 11.7) | 0.2 (0 to 0.5) | 0.7 (0.1 to 2.1) | -5.93 (-6.3 to -5.55) |
| China | 392.5 (170.3 to 720.7) | 40.9 (17.3 to 78.2) | 163.9 (68.5 to 304.4) | 8.2 (3.3 to 15.2) | -5.5 (-5.72 to -5.28) |
| Colombia | 0.7 (0.1 to 1.6) | 3.1 (0.6 to 7.4) | 0.3 (0.1 to 0.7) | 0.6 (0.1 to 1.3) | -5.96 (-6.48 to -5.43) |
| Comoros | 0 (0 to 0.1) | 6.8 (0.4 to 20.2) | 0 (0 to 0.1) | 2.9 (0.2 to 10) | -2.92 (-3.13 to -2.7) |
| Congo | 0.1 (0 to 0.3) | 4.4 (0.4 to 18.2) | 0.1 (0 to 0.4) | 2.5 (0.2 to 10.3) | -1.94 (-2.23 to -1.65) |
| Cook Islands | 0 (0 to 0) | 3.3 (0.5 to 9.5) | 0 (0 to 0) | 1.7 (0.2 to 5) | -2.37 (-2.78 to -1.96) |
| Costa Rica | 0.1 (0 to 0.3) | 3.9 (0.3 to 10.8) | 0.1 (0 to 0.3) | 2.4 (0.2 to 6.6) | -1.62 (-1.87 to -1.36) |
| Croatia | 0.6 (0.2 to 1.2) | 9.7 (3.6 to 18.3) | 0.1 (0 to 0.3) | 1.7 (0.5 to 3.6) | -5.34 (-6.44 to -4.22) |
| Cuba | 0.2 (0 to 0.7) | 1.8 (0.2 to 6.7) | 0.2 (0 to 0.6) | 1 (0.1 to 3.8) | -1.7 (-2.03 to -1.37) |
| Cyprus | 0 (0 to 0.1) | 2.2 (0.2 to 7.8) | 0 (0 to 0.1) | 0.8 (0.1 to 2.7) | -3.57 (-3.89 to -3.23) |
| Czechia | 1.9 (0.7 to 3.6) | 14.7 (5.8 to 27.6) | 0.4 (0.1 to 0.9) | 2.1 (0.6 to 4.5) | -6.52 (-6.76 to -6.29) |
| Côte d'Ivoire | 0.2 (0 to 0.9) | 4 (0.3 to 15.1) | 0.4 (0 to 1.5) | 2.2 (0.1 to 8.5) | -2.09 (-2.26 to -1.91) |
| Democratic People's Republic of Korea | 5.4 (2 to 10.4) | 29.2 (10.5 to 56.7) | 4.2 (0.9 to 10.2) | 12.9 (2.7 to 31.7) | -2.72 (-2.92 to -2.53) |
| Democratic Republic of the Congo | 0.6 (0.1 to 2.6) | 2.6 (0.4 to 11.8) | 1 (0.1 to 4.8) | 1.8 (0.2 to 8.8) | -1.29 (-1.38 to -1.21) |
| Denmark | 0.1 (0 to 0.3) | 1.3 (0.1 to 4.1) | 0 (0 to 0.1) | 0.2 (0 to 0.8) | -5.65 (-6.63 to -4.67) |
| Djibouti | 0 (0 to 0) | 5.8 (0.4 to 17) | 0 (0 to 0.1) | 2.5 (0.2 to 8.5) | -2.88 (-3.02 to -2.75) |
| Dominica | 0 (0 to 0) | 2.2 (0.3 to 8.2) | 0 (0 to 0) | 1.3 (0.1 to 4.9) | -1.83 (-1.92 to -1.75) |
| Dominican Republic | 0.1 (0 to 0.3) | 1.4 (0.2 to 5.4) | 0.1 (0 to 0.4) | 1 (0.1 to 3.9) | -1.12 (-1.57 to -0.66) |
| Ecuador | 0.2 (0 to 0.6) | 3.2 (0.2 to 9.3) | 0.2 (0 to 0.7) | 1.4 (0.1 to 4.3) | -2.79 (-3.18 to -2.39) |
| Egypt | 0.6 (0.1 to 2.4) | 1.5 (0.3 to 6) | 0.6 (0.1 to 2.5) | 0.8 (0.1 to 2.9) | -2.31 (-2.49 to -2.13) |
| El Salvador | 0.1 (0 to 0.3) | 2.4 (0.2 to 6.6) | 0.1 (0 to 0.3) | 1.6 (0.2 to 4.5) | -1.32 (-1.6 to -1.04) |
| Equatorial Guinea | 0 (0 to 0.1) | 5 (0.4 to 21.7) | 0 (0 to 0.1) | 1.8 (0.1 to 7.6) | -3.44 (-3.59 to -3.29) |
| Eritrea | 0.1 (0 to 0.5) | 10.1 (0.7 to 31.2) | 0.1 (0 to 0.6) | 4.2 (0.3 to 14.2) | -2.97 (-3.11 to -2.83) |
| Estonia | 0 (0 to 0.1) | 2.1 (0.4 to 7.2) | 0 (0 to 0) | 0.4 (0.1 to 1.3) | -5.81 (-6.37 to -5.24) |
| Eswatini | 0 (0 to 0.1) | 6 (0.4 to 22.2) | 0 (0 to 0.1) | 3.1 (0.2 to 12.8) | -2.24 (-2.43 to -2.06) |
| Ethiopia | 2.4 (0.2 to 7.9) | 9.6 (0.8 to 28.5) | 1.5 (0.1 to 5.6) | 2.8 (0.2 to 9.4) | -4.22 (-4.34 to -4.1) |
| Fiji | 0 (0 to 0.1) | 9.6 (1.5 to 28) | 0 (0 to 0.1) | 6.1 (0.7 to 17.6) | -1.56 (-1.93 to -1.2) |
| Finland | 0.1 (0 to 0.2) | 0.9 (0.1 to 2.7) | 0 (0 to 0.1) | 0.2 (0 to 0.5) | -5.61 (-5.92 to -5.3) |
| France | 0.6 (0.1 to 2.2) | 0.7 (0.1 to 2.7) | 0.6 (0.1 to 2) | 0.4 (0 to 1.4) | -1.87 (-2.11 to -1.62) |
| Gabon | 0 (0 to 0.1) | 3.1 (0.3 to 12.9) | 0 (0 to 0.1) | 1.9 (0.1 to 7.8) | -1.67 (-1.78 to -1.56) |
| Gambia | 0 (0 to 0.1) | 3.2 (0.2 to 12.6) | 0 (0 to 0.1) | 2.1 (0.1 to 8.7) | -1.34 (-2 to -0.67) |
| Georgia | 0.4 (0.1 to 1) | 6.8 (1.3 to 16.2) | 0.3 (0 to 0.7) | 4.9 (0.5 to 13.5) | -1.05 (-1.49 to -0.6) |
| Germany | 1.8 (0.2 to 6) | 1.5 (0.2 to 4.9) | 1.2 (0.1 to 4) | 0.6 (0.1 to 2.1) | -2.98 (-3.39 to -2.56) |
| Ghana | 0.3 (0 to 1.1) | 3.2 (0.2 to 12.1) | 0.6 (0 to 2) | 2.4 (0.1 to 8.2) | -0.86 (-1.05 to -0.68) |
| Greece | 0.1 (0 to 0.3) | 0.5 (0.1 to 1.8) | 0 (0 to 0.2) | 0.2 (0 to 0.7) | -2.74 (-3.33 to -2.15) |
| Greenland | 0 (0 to 0) | 2.3 (0.2 to 7.6) | 0 (0 to 0) | 0.8 (0.1 to 2.5) | -3.61 (-3.86 to -3.35) |
| Grenada | 0 (0 to 0) | 3.7 (0.4 to 13.8) | 0 (0 to 0) | 1.7 (0.2 to 6.1) | -2.7 (-2.82 to -2.59) |
| Guam | 0 (0 to 0) | 3.4 (0.5 to 9.4) | 0 (0 to 0) | 1.8 (0.2 to 5.7) | -2.15 (-2.49 to -1.8) |
| Guatemala | 0.1 (0 to 0.3) | 2.1 (0.2 to 5.8) | 0.2 (0 to 0.6) | 1.5 (0.1 to 4.1) | -1.22 (-1.72 to -0.72) |
| Guinea | 0.2 (0 to 0.7) | 4.5 (0.3 to 18.8) | 0.2 (0 to 0.7) | 2.6 (0.2 to 10) | -1.95 (-2.06 to -1.83) |
| Guinea-Bissau | 0 (0 to 0.1) | 6.7 (0.5 to 27.3) | 0 (0 to 0.1) | 3.3 (0.2 to 13.1) | -2.44 (-2.53 to -2.35) |
| Guyana | 0 (0 to 0.1) | 2.8 (0.3 to 10.3) | 0 (0 to 0) | 1.6 (0.2 to 5.9) | -1.84 (-2.16 to -1.52) |
| Haiti | 0.4 (0 to 1.5) | 8.9 (0.9 to 33.9) | 0.4 (0 to 1.8) | 4.4 (0.4 to 18.6) | -2.3 (-2.44 to -2.16) |
| Honduras | 0.1 (0 to 0.2) | 2 (0.2 to 5.6) | 0.1 (0 to 0.4) | 1.7 (0.2 to 4.8) | -0.56 (-0.8 to -0.31) |
| Hungary | 3 (1.4 to 5.3) | 21.5 (9.9 to 38.1) | 0.5 (0.2 to 1) | 2.6 (0.9 to 5.1) | -7.1 (-7.57 to -6.63) |
| Iceland | 0 (0 to 0) | 0.4 (0 to 1.4) | 0 (0 to 0) | 0.2 (0 to 0.7) | -2.32 (-2.46 to -2.17) |
| India | 105.2 (10.7 to 301.4) | 17.6 (1.8 to 50.7) | 132.2 (14.9 to 366.1) | 10.1 (1.1 to 27.9) | -1.86 (-2.22 to -1.5) |
| Indonesia | 5 (1.2 to 11.1) | 4 (1 to 8.4) | 2.3 (0.4 to 5.8) | 1 (0.1 to 2.4) | -4.84 (-5.02 to -4.65) |
| Iran (Islamic Republic of) | 0.4 (0.1 to 1.5) | 1.1 (0.2 to 4.2) | 0.5 (0.1 to 2) | 0.6 (0.1 to 2.1) | -2.16 (-2.44 to -1.89) |
| Iraq | 0.2 (0 to 0.7) | 1.8 (0.3 to 7.2) | 0.2 (0 to 0.8) | 0.7 (0.1 to 2.4) | -3.52 (-3.75 to -3.28) |
| Ireland | 0 (0 to 0.1) | 0.7 (0.1 to 2.7) | 0 (0 to 0.1) | 0.2 (0 to 0.9) | -3.7 (-4.23 to -3.18) |
| Israel | 0.1 (0 to 0.2) | 1.2 (0.1 to 4) | 0.1 (0 to 0.3) | 0.7 (0.1 to 2.3) | -1.83 (-2.84 to -0.8) |
| Italy | 2.2 (0.3 to 5.9) | 2.6 (0.3 to 7.1) | 1 (0.1 to 3.1) | 0.8 (0.1 to 2.4) | -3.8 (-4.01 to -3.58) |
| Jamaica | 0 (0 to 0.1) | 1.5 (0.2 to 5.8) | 0 (0 to 0.1) | 1 (0.1 to 3.6) | -1.36 (-1.85 to -0.88) |
| Japan | 4.8 (1.4 to 9.9) | 2.9 (0.8 to 6) | 2.1 (0.3 to 6.1) | 0.6 (0.1 to 1.4) | -5.58 (-5.82 to -5.33) |
| Jordan | 0 (0 to 0) | 0.4 (0.1 to 1.6) | 0 (0 to 0) | 0.1 (0 to 0.4) | -4.2 (-4.44 to -3.95) |
| Kazakhstan | 2 (0.3 to 4.7) | 13.1 (2.4 to 31.1) | 0.4 (0 to 1.1) | 2 (0.2 to 5.7) | -6.36 (-6.85 to -5.87) |
| Kenya | 0.4 (0 to 1.2) | 3.2 (0.3 to 10.1) | 0.6 (0.1 to 2.1) | 1.9 (0.2 to 6.4) | -1.84 (-1.91 to -1.77) |
| Kiribati | 0 (0 to 0) | 18.5 (2.9 to 56.1) | 0 (0 to 0) | 12.4 (1.5 to 39.7) | -1.39 (-1.5 to -1.29) |
| Kuwait | 0 (0 to 0) | 0.8 (0.1 to 2.8) | 0 (0 to 0) | 0.2 (0 to 0.6) | -4.75 (-5.63 to -3.85) |
| Kyrgyzstan | 0.5 (0.1 to 1.3) | 15.5 (2.7 to 39.7) | 0.2 (0 to 0.7) | 3.8 (0.4 to 11) | -4.71 (-5.34 to -4.07) |
| Lao People's Democratic Republic | 0.4 (0.1 to 1) | 14.9 (3.1 to 35.5) | 0.3 (0 to 0.9) | 5 (0.6 to 14.2) | -3.69 (-3.78 to -3.6) |
| Latvia | 0.2 (0 to 0.6) | 5.6 (0.6 to 17.2) | 0 (0 to 0.1) | 0.9 (0.1 to 2.8) | -6 (-7.91 to -4.05) |
| Lebanon | 0 (0 to 0.1) | 0.7 (0.1 to 2.8) | 0 (0 to 0.1) | 0.3 (0 to 1) | -3.65 (-3.72 to -3.57) |
| Lesotho | 0.1 (0 to 0.3) | 6.2 (0.3 to 23.3) | 0.1 (0 to 0.3) | 4.1 (0.3 to 16) | -1.42 (-1.6 to -1.23) |
| Liberia | 0 (0 to 0.2) | 3.7 (0.3 to 14.4) | 0.1 (0 to 0.3) | 2 (0.1 to 8.4) | -2.08 (-2.24 to -1.92) |
| Libya | 0 (0 to 0.1) | 0.8 (0.1 to 3) | 0 (0 to 0.2) | 0.5 (0.1 to 2) | -1.31 (-1.5 to -1.12) |
| Lithuania | 0.2 (0 to 0.8) | 5.4 (0.5 to 18.2) | 0 (0 to 0.2) | 1 (0.1 to 3.5) | -5.91 (-6.71 to -5.1) |
| Luxembourg | 0 (0 to 0) | 0.9 (0.1 to 2.9) | 0 (0 to 0) | 0.4 (0 to 1.4) | -2.41 (-2.56 to -2.27) |
| Madagascar | 0.6 (0 to 2.1) | 9.6 (0.6 to 28.6) | 0.7 (0 to 2.6) | 4.7 (0.3 to 16.1) | -2.44 (-2.55 to -2.32) |
| Malawi | 0.3 (0 to 1) | 6.3 (0.4 to 18.9) | 0.3 (0 to 1) | 2.8 (0.2 to 9.6) | -2.74 (-2.81 to -2.68) |
| Malaysia | 1 (0.2 to 2.3) | 8.6 (2 to 18.6) | 0.6 (0.1 to 1.5) | 1.8 (0.2 to 4.6) | -5.25 (-5.6 to -4.89) |
| Maldives | 0 (0 to 0) | 10.1 (2 to 24.8) | 0 (0 to 0) | 1.9 (0.2 to 4.8) | -5.66 (-5.81 to -5.51) |
| Mali | 0.3 (0 to 1.1) | 5.3 (0.4 to 21.9) | 0.3 (0 to 1.3) | 2.6 (0.2 to 10.6) | -2.45 (-2.7 to -2.2) |
| Malta | 0 (0 to 0) | 1.2 (0.1 to 3.6) | 0 (0 to 0) | 0.6 (0.1 to 1.7) | -2.35 (-2.53 to -2.17) |
| Marshall Islands | 0 (0 to 0) | 14.6 (2.2 to 45.8) | 0 (0 to 0) | 9.8 (1.1 to 32.7) | -1.37 (-1.6 to -1.14) |
| Mauritania | 0 (0 to 0.2) | 3.7 (0.3 to 14.6) | 0 (0 to 0.2) | 1.5 (0.1 to 6.4) | -3.02 (-3.18 to -2.86) |
| Mauritius | 0.1 (0 to 0.1) | 6.8 (1.4 to 15.3) | 0 (0 to 0.1) | 1.6 (0.2 to 4.2) | -4.73 (-5.35 to -4.1) |
| Mexico | 1.7 (0.2 to 5.3) | 3 (0.3 to 9.5) | 1.1 (0.1 to 3.3) | 0.9 (0.1 to 2.6) | -4.22 (-4.39 to -4.04) |
| Micronesia (Federated States of) | 0 (0 to 0) | 15.2 (2.3 to 47.6) | 0 (0 to 0) | 9 (1 to 30.3) | -1.81 (-1.88 to -1.74) |
| Monaco | 0 (0 to 0) | 0.4 (0 to 1.2) | 0 (0 to 0) | 0.2 (0 to 0.8) | -1.28 (-1.39 to -1.18) |
| Mongolia | 0.2 (0 to 0.5) | 17.1 (2.9 to 44.6) | 0.2 (0 to 0.5) | 5.2 (0.5 to 14.8) | -3.98 (-4.19 to -3.77) |
| Montenegro | 0 (0 to 0.1) | 5.4 (2 to 10.4) | 0 (0 to 0) | 2.5 (0.6 to 5.2) | -2.56 (-2.81 to -2.31) |
| Morocco | 0.3 (0 to 1.2) | 1.7 (0.3 to 6.4) | 0.3 (0.1 to 1.1) | 0.8 (0.2 to 3.1) | -2.46 (-2.54 to -2.39) |
| Mozambique | 0.5 (0 to 1.4) | 6.5 (0.5 to 19) | 0.5 (0 to 1.8) | 3.3 (0.2 to 11.3) | -2.27 (-2.33 to -2.21) |
| Myanmar | 3.4 (0.7 to 7.8) | 12 (2.8 to 27.2) | 1.9 (0.2 to 4.9) | 3.6 (0.5 to 9.1) | -4.09 (-4.2 to -3.97) |
| Namibia | 0.1 (0 to 0.2) | 5.9 (0.3 to 21.4) | 0.1 (0 to 0.2) | 2.5 (0.2 to 9.6) | -2.96 (-3.18 to -2.75) |
| Nauru | 0 (0 to 0) | 9.4 (1.5 to 27.7) | 0 (0 to 0) | 7.1 (0.8 to 22.6) | -1.04 (-1.25 to -0.84) |
| Nepal | 1.6 (0.1 to 5.9) | 13.1 (1.2 to 49.1) | 2.2 (0.2 to 6.9) | 9 (0.7 to 28) | -1.23 (-1.37 to -1.08) |
| Netherlands | 0.1 (0 to 0.2) | 0.3 (0 to 1.2) | 0.1 (0 to 0.3) | 0.2 (0 to 0.8) | -1.23 (-1.93 to -0.52) |
| New Zealand | 0.1 (0 to 0.3) | 2.2 (0.2 to 7.1) | 0.1 (0 to 0.2) | 1 (0.1 to 3.1) | -2.78 (-3.21 to -2.35) |
| Nicaragua | 0.1 (0 to 0.2) | 2.9 (0.2 to 8.3) | 0.1 (0 to 0.3) | 1.7 (0.2 to 4.8) | -1.93 (-2.14 to -1.72) |
| Niger | 0.2 (0 to 0.8) | 5.1 (0.4 to 20.9) | 0.3 (0 to 1.3) | 2.8 (0.2 to 11.7) | -1.95 (-2.07 to -1.83) |
| Nigeria | 1.9 (0.2 to 7.8) | 3.6 (0.3 to 14.8) | 2 (0.2 to 8.6) | 1.6 (0.1 to 6.4) | -2.84 (-2.96 to -2.72) |
| Niue | 0 (0 to 0) | 5.8 (0.9 to 17) | 0 (0 to 0) | 3.4 (0.4 to 10.3) | -1.89 (-1.99 to -1.8) |
| North Macedonia | 0.2 (0.1 to 0.4) | 10.5 (3.7 to 21.1) | 0.1 (0 to 0.2) | 2.3 (0.6 to 5) | -5.06 (-5.27 to -4.86) |
| Northern Mariana Islands | 0 (0 to 0) | 3 (0.4 to 9) | 0 (0 to 0) | 2.2 (0.3 to 6.6) | -1.07 (-1.18 to -0.96) |
| Norway | 0 (0 to 0.1) | 0.6 (0.1 to 1.9) | 0 (0 to 0.1) | 0.2 (0 to 0.7) | -3.55 (-4.03 to -3.07) |
| Oman | 0 (0 to 0) | 0.4 (0.1 to 1.6) | 0 (0 to 0) | 0.1 (0 to 0.4) | -4.23 (-4.46 to -4) |
| Pakistan | 9 (0.9 to 30.8) | 13.3 (1.3 to 46.1) | 20.2 (1.8 to 61.4) | 13.6 (1.2 to 41.5) | 0.11 (0 to 0.23) |
| Palau | 0 (0 to 0) | 4.2 (0.6 to 12.9) | 0 (0 to 0) | 2.6 (0.3 to 7.8) | -1.61 (-1.68 to -1.54) |
| Palestine | 0 (0 to 0) | 0.7 (0.1 to 2.7) | 0 (0 to 0.1) | 0.4 (0.1 to 1.5) | -2.14 (-2.2 to -2.08) |
| Panama | 0.1 (0 to 0.2) | 3.5 (0.3 to 10.2) | 0.1 (0 to 0.2) | 1.8 (0.2 to 4.9) | -2.29 (-2.72 to -1.85) |
| Papua New Guinea | 0.3 (0 to 1) | 14.3 (1.9 to 45.3) | 0.7 (0.1 to 2.5) | 12.4 (1.3 to 40.8) | -0.51 (-0.6 to -0.42) |
| Paraguay | 0.1 (0 to 0.2) | 2.1 (0.2 to 6.4) | 0.1 (0 to 0.3) | 1.5 (0.1 to 4.7) | -1.18 (-1.55 to -0.8) |
| Peru | 0.3 (0 to 1) | 2.2 (0.2 to 6.6) | 0.4 (0 to 1.3) | 1.2 (0.1 to 3.7) | -2.06 (-2.44 to -1.68) |
| Philippines | 1.6 (0.3 to 3.7) | 3.7 (0.8 to 8.4) | 3.3 (0.4 to 9.3) | 3.2 (0.4 to 8.9) | -0.4 (-0.83 to 0.04) |
| Poland | 4.7 (0.8 to 11.5) | 10.8 (1.8 to 26.9) | 1.1 (0.2 to 2.8) | 1.8 (0.3 to 4.1) | -6.17 (-6.67 to -5.66) |
| Portugal | 0.2 (0 to 0.7) | 1.7 (0.2 to 5.6) | 0.1 (0 to 0.3) | 0.6 (0.1 to 1.7) | -3.79 (-4.43 to -3.13) |
| Puerto Rico | 0 (0 to 0.1) | 0.6 (0.1 to 2.1) | 0 (0 to 0) | 0.2 (0 to 0.8) | -3.08 (-3.43 to -2.73) |
| Qatar | 0 (0 to 0) | 0.7 (0.1 to 2.6) | 0 (0 to 0) | 0.2 (0 to 0.6) | -4.62 (-4.81 to -4.43) |
| Republic of Korea | 0.5 (0.1 to 1.2) | 1.6 (0.3 to 3.6) | 0.4 (0.1 to 0.9) | 0.5 (0.1 to 1) | -4.23 (-4.38 to -4.08) |
| Republic of Moldova | 0.2 (0 to 0.8) | 4.9 (0.6 to 16.4) | 0 (0 to 0.2) | 0.9 (0.1 to 3.3) | -5.62 (-6.9 to -4.32) |
| Romania | 5.1 (1.8 to 9.5) | 18.7 (6.7 to 34.5) | 0.8 (0.2 to 1.7) | 2.4 (0.7 to 5.1) | -6.8 (-7.05 to -6.54) |
| Russian Federation | 14 (1.7 to 35.6) | 7.7 (1 to 19.5) | 3.3 (0.4 to 8.7) | 1.5 (0.2 to 3.9) | -5.56 (-6.61 to -4.51) |
| Rwanda | 0.4 (0 to 1.2) | 10.6 (0.7 to 31.6) | 0.2 (0 to 0.9) | 2.8 (0.2 to 10) | -4.48 (-4.65 to -4.3) |
| Saint Kitts and Nevis | 0 (0 to 0) | 2 (0.2 to 7.6) | 0 (0 to 0) | 0.5 (0 to 1.9) | -4.58 (-4.84 to -4.31) |
| Saint Lucia | 0 (0 to 0) | 3 (0.4 to 11.1) | 0 (0 to 0) | 1.3 (0.1 to 4.9) | -2.68 (-2.94 to -2.43) |
| Saint Vincent and the Grenadines | 0 (0 to 0) | 2.3 (0.3 to 8.7) | 0 (0 to 0) | 1.4 (0.2 to 5.2) | -1.61 (-1.81 to -1.41) |
| Samoa | 0 (0 to 0) | 2.7 (0.7 to 11) | 0 (0 to 0) | 2.1 (0.5 to 7.8) | -0.97 (-1.03 to -0.9) |
| San Marino | 0 (0 to 0) | 1 (0.1 to 3.5) | 0 (0 to 0) | 0.8 (0.1 to 2.6) | -0.92 (-1.05 to -0.79) |
| Sao Tome and Principe | 0 (0 to 0) | 4.7 (0.4 to 18.8) | 0 (0 to 0) | 3.2 (0.2 to 13.5) | -1.21 (-1.36 to -1.05) |
| Saudi Arabia | 0.1 (0 to 0.4) | 0.9 (0.2 to 3.7) | 0.1 (0 to 0.4) | 0.3 (0.1 to 1.1) | -3.81 (-4.07 to -3.55) |
| Senegal | 0.1 (0 to 0.5) | 3.4 (0.2 to 13.5) | 0.2 (0 to 0.8) | 2 (0.1 to 8) | -1.84 (-2.04 to -1.63) |
| Serbia | 0.7 (0.2 to 1.4) | 5.8 (2 to 12.3) | 0.2 (0.1 to 0.5) | 1.6 (0.4 to 3.3) | -4.41 (-4.77 to -4.05) |
| Seychelles | 0 (0 to 0) | 6.8 (1.6 to 15.1) | 0 (0 to 0) | 1 (0.1 to 2.6) | -6.51 (-6.82 to -6.19) |
| Sierra Leone | 0.1 (0 to 0.3) | 3.7 (0.3 to 14.8) | 0.1 (0 to 0.5) | 2.3 (0.2 to 9.5) | -1.59 (-1.7 to -1.49) |
| Singapore | 0.1 (0 to 0.2) | 3.9 (0.8 to 8.5) | 0 (0 to 0.1) | 0.5 (0.1 to 1.1) | -7.1 (-7.4 to -6.8) |
| Slovakia | 0.4 (0.1 to 0.7) | 6.4 (2.4 to 12.2) | 0.2 (0.1 to 0.4) | 2.1 (0.6 to 4.6) | -3.76 (-4.01 to -3.51) |
| Slovenia | 0.2 (0.1 to 0.5) | 10 (3.6 to 19.8) | 0.1 (0 to 0.3) | 2.9 (0.8 to 6.4) | -4.14 (-4.44 to -3.84) |
| Solomon Islands | 0 (0 to 0.1) | 19.6 (2.7 to 62.7) | 0.1 (0 to 0.2) | 14.2 (1.5 to 48.7) | -1.12 (-1.3 to -0.94) |
| Somalia | 0.4 (0 to 1.2) | 11 (0.7 to 34.1) | 0.5 (0 to 1.8) | 5.3 (0.3 to 18.8) | -2.45 (-2.53 to -2.38) |
| South Africa | 1.3 (0.1 to 5.1) | 4.2 (0.3 to 16.1) | 0.9 (0.1 to 3.8) | 1.6 (0.2 to 6.6) | -3.41 (-3.91 to -2.92) |
| South Sudan | 0.2 (0 to 0.6) | 6.2 (0.4 to 19.2) | 0.1 (0 to 0.5) | 2.9 (0.2 to 10.4) | -2.58 (-2.65 to -2.52) |
| Spain | 0.6 (0.1 to 2.4) | 1.3 (0.2 to 4.7) | 0.4 (0.1 to 1.5) | 0.5 (0.1 to 1.7) | -3.17 (-3.37 to -2.97) |
| Sri Lanka | 0.7 (0.2 to 1.6) | 5.5 (1.3 to 11.8) | 0.3 (0 to 0.7) | 1.1 (0.1 to 2.7) | -5.28 (-5.65 to -4.9) |
| Sudan | 0.3 (0 to 1.2) | 2.3 (0.3 to 9.4) | 0.3 (0 to 1.1) | 1 (0.2 to 3.9) | -2.77 (-2.84 to -2.69) |
| Suriname | 0 (0 to 0) | 1.5 (0.2 to 5.6) | 0 (0 to 0) | 1 (0.1 to 3.6) | -1.6 (-1.94 to -1.26) |
| Sweden | 0.1 (0 to 0.3) | 0.6 (0.1 to 2) | 0.1 (0 to 0.2) | 0.2 (0 to 0.8) | -3.36 (-3.61 to -3.1) |
| Switzerland | 0.1 (0 to 0.3) | 0.9 (0.1 to 3.1) | 0 (0 to 0.1) | 0.2 (0 to 0.7) | -4.93 (-5.17 to -4.7) |
| Syrian Arab Republic | 0.2 (0 to 1) | 3.4 (0.6 to 13.3) | 0.1 (0 to 0.3) | 0.7 (0.1 to 2.5) | -5.46 (-5.87 to -5.06) |
| Taiwan (Province of China) | 0.7 (0.1 to 1.8) | 4.5 (0.6 to 11.2) | 0.3 (0 to 0.7) | 0.7 (0.1 to 1.9) | -6.28 (-6.59 to -5.97) |
| Tajikistan | 0.5 (0.1 to 1.2) | 14 (2.5 to 34.6) | 0.3 (0 to 1) | 4 (0.4 to 11.8) | -4.17 (-4.91 to -3.43) |
| Thailand | 4.1 (0.9 to 9.6) | 9 (2 to 20.3) | 1.1 (0.1 to 2.7) | 1.1 (0.1 to 3) | -7.06 (-7.46 to -6.65) |
| Timor-Leste | 0 (0 to 0.1) | 9.8 (1.9 to 23.7) | 0 (0 to 0.1) | 4.4 (0.5 to 12) | -2.8 (-3.07 to -2.54) |
| Togo | 0.1 (0 to 0.3) | 3.7 (0.3 to 14.6) | 0.1 (0 to 0.5) | 2.1 (0.1 to 8.8) | -1.89 (-2.07 to -1.72) |
| Tokelau | 0 (0 to 0) | 8.8 (1.3 to 27) | 0 (0 to 0) | 4.3 (0.5 to 13.6) | -2.44 (-2.46 to -2.41) |
| Tonga | 0 (0 to 0) | 3.6 (0.5 to 10.9) | 0 (0 to 0) | 2.5 (0.3 to 7.7) | -1.22 (-1.37 to -1.06) |
| Trinidad and Tobago | 0 (0 to 0.1) | 1.9 (0.2 to 6.9) | 0 (0 to 0.1) | 1 (0.1 to 3.5) | -2.31 (-2.68 to -1.94) |
| Tunisia | 0 (0 to 0.2) | 0.7 (0.1 to 2.7) | 0 (0 to 0.1) | 0.3 (0.1 to 1) | -3.14 (-3.2 to -3.07) |
| Turkey | NA | NA | NA | NA | NA |
| Turkmenistan | 0.2 (0 to 0.6) | 9.6 (1.8 to 23.6) | 0.2 (0 to 0.5) | 3.2 (0.3 to 9.1) | -3.65 (-4.52 to -2.78) |
| Tuvalu | 0 (0 to 0) | 13.5 (1.9 to 42.7) | 0 (0 to 0) | 7.2 (0.8 to 23.9) | -2.11 (-2.26 to -1.96) |
| Uganda | 0.5 (0 to 1.6) | 6.2 (0.4 to 17.8) | 0.5 (0 to 2.1) | 2.7 (0.2 to 9.3) | -2.89 (-3.07 to -2.71) |
| Ukraine | 1 (0.1 to 3.5) | 1.4 (0.2 to 5) | 0.6 (0.1 to 2.1) | 1 (0.1 to 3.4) | -1.18 (-2.46 to 0.12) |
| United Arab Emirates | 0 (0 to 0.1) | 3 (0.4 to 12.2) | 0.1 (0 to 0.5) | 1.4 (0.2 to 5.5) | -2.65 (-2.92 to -2.38) |
| United Kingdom | 0.8 (0.1 to 2.8) | 0.9 (0.2 to 3.2) | 0.3 (0.1 to 1.1) | 0.3 (0 to 0.9) | -4.09 (-4.25 to -3.93) |
| United Republic of Tanzania | 0.8 (0.1 to 2.5) | 6.3 (0.5 to 17.8) | 1 (0.1 to 3.6) | 3.3 (0.2 to 10.5) | -2.16 (-2.29 to -2.02) |
| United States of America | 3.3 (0.4 to 11) | 1.1 (0.1 to 3.6) | 2.9 (0.3 to 8.7) | 0.6 (0.1 to 1.7) | -2.17 (-2.37 to -1.96) |
| United States Virgin Islands | 0 (0 to 0) | 0.8 (0.1 to 3.1) | 0 (0 to 0) | 0.4 (0 to 1.6) | -2.09 (-2.29 to -1.9) |
| Uruguay | 0.1 (0 to 0.2) | 1.8 (0.2 to 5) | 0 (0 to 0.1) | 0.9 (0.1 to 2.6) | -2.41 (-2.8 to -2.03) |
| Uzbekistan | 2.1 (0.4 to 5.2) | 14.2 (2.7 to 34.7) | 2 (0.2 to 5.8) | 6.2 (0.7 to 17.7) | -2.78 (-3.42 to -2.13) |
| Vanuatu | 0 (0 to 0) | 13.7 (1.9 to 42.2) | 0 (0 to 0.1) | 11.6 (1.3 to 36.7) | -0.59 (-0.75 to -0.42) |
| Venezuela (Bolivarian Republic of) | 0.3 (0 to 0.8) | 2.1 (0.2 to 5.9) | 0.2 (0 to 0.6) | 0.7 (0.1 to 1.9) | -3.89 (-4.3 to -3.48) |
| Viet Nam | 3.2 (0.7 to 7.1) | 7.4 (1.6 to 16.7) | 2 (0.2 to 5) | 2 (0.2 to 5) | -4.38 (-4.46 to -4.31) |
| Yemen | 0.2 (0 to 0.7) | 2.4 (0.4 to 9.5) | 0.3 (0 to 1) | 1.4 (0.2 to 4.9) | -1.95 (-2.14 to -1.75) |
| Zambia | 0.2 (0 to 0.7) | 6.4 (0.4 to 18.8) | 0.3 (0 to 1.1) | 3.1 (0.2 to 10.6) | -2.4 (-2.56 to -2.23) |
| Zimbabwe | 0.5 (0 to 1.6) | 7 (0.4 to 23.9) | 0.8 (0 to 2.9) | 6.6 (0.3 to 24.3) | -0.24 (-0.4 to -0.08) |
