# Supplementary material for: Spatiotemporal patterns of rheumatic heart disease burden attributable to high systolic blood pressure, high sodium diet, and lead exposure (1990 to 2019): a longitudinal observational study
Source: Front Nutr. 2024 Sep 26;11:1419349. doi: 10.3389/fnut.2024.1419349 (PMC11466049; doi:10.3389/fnut.2024.1419349)
Supplement: Supplementary file 5 [file Table_5.docx]

**Supplementary table 5. Deaths of rheumatic heart disease due to lead exposure**

| **location** | **1990 Counts**  **(thousand)** | **Age-standardised rate (per 100 000 population), 1990** | **2019 Counts**  **(thousand)** | **Age-standardised rate (per 100 000 population), 2019** | **Average annual percent change** |
| --- | --- | --- | --- | --- | --- |
| Afghanistan | 0 (0 to 0.1) | 0.5 (0.2 to 1.2) | 0 (0 to 0.1) | 0.3 (0.1 to 0.7) | -1.69 (-1.82 to -1.56) |
| Albania | 0 (0 to 0) | 0.1 (0 to 0.1) | 0 (0 to 0) | 0 (0 to 0) | -5.4 (-6.06 to -4.73) |
| Algeria | 0 (0 to 0) | 0.1 (0 to 0.1) | 0 (0 to 0) | 0 (0 to 0) | -4.04 (-4.12 to -3.95) |
| American Samoa | 0 (0 to 0) | 0 (0 to 0.1) | 0 (0 to 0) | 0 (0 to 0) | -2.9 (-3.22 to -2.57) |
| Andorra | 0 (0 to 0) | 0 (0 to 0) | 0 (0 to 0) | 0 (0 to 0) | -2.32 (-2.55 to -2.08) |
| Angola | 0 (0 to 0) | 0.1 (0 to 0.2) | 0 (0 to 0) | 0.1 (0 to 0.1) | -2.55 (-2.73 to -2.38) |
| Antigua and Barbuda | 0 (0 to 0) | 0 (0 to 0) | 0 (0 to 0) | 0 (0 to 0) | -3.57 (-3.88 to -3.25) |
| Argentina | 0 (0 to 0) | 0 (0 to 0.1) | 0 (0 to 0) | 0 (0 to 0) | -2.68 (-2.91 to -2.44) |
| Armenia | 0 (0 to 0) | 0.1 (0 to 0.1) | 0 (0 to 0) | 0 (0 to 0.1) | -3.26 (-4.01 to -2.51) |
| Australia | 0 (0 to 0) | 0 (0 to 0.1) | 0 (0 to 0) | 0 (0 to 0) | -3.23 (-3.54 to -2.93) |
| Austria | 0 (0 to 0) | 0 (0 to 0) | 0 (0 to 0) | 0 (0 to 0) | -2.75 (-3 to -2.51) |
| Azerbaijan | 0 (0 to 0) | 0 (0 to 0.1) | 0 (0 to 0) | 0 (0 to 0) | -2.12 (-2.5 to -1.73) |
| Bahamas | 0 (0 to 0) | 0 (0 to 0) | 0 (0 to 0) | 0 (0 to 0) | -3.67 (-3.93 to -3.41) |
| Bahrain | 0 (0 to 0) | 0 (0 to 0.1) | 0 (0 to 0) | 0 (0 to 0) | -3.18 (-3.57 to -2.8) |
| Bangladesh | 0.1 (0.1 to 0.2) | 0.2 (0.1 to 0.3) | 0.1 (0 to 0.2) | 0.1 (0 to 0.2) | -2.54 (-3.29 to -1.79) |
| Barbados | 0 (0 to 0) | 0 (0 to 0) | 0 (0 to 0) | 0 (0 to 0) | -3.79 (-4.1 to -3.48) |
| Belarus | 0 (0 to 0) | 0 (0 to 0.1) | 0 (0 to 0) | 0 (0 to 0) | -4.79 (-5.49 to -4.08) |
| Belgium | 0 (0 to 0) | 0 (0 to 0) | 0 (0 to 0) | 0 (0 to 0.1) | 1.12 (0.76 to 1.48) |
| Belize | 0 (0 to 0) | 0 (0 to 0.1) | 0 (0 to 0) | 0 (0 to 0) | -3.69 (-3.99 to -3.39) |
| Benin | 0 (0 to 0) | 0.1 (0 to 0.2) | 0 (0 to 0) | 0.1 (0 to 0.1) | -2.62 (-2.73 to -2.51) |
| Bermuda | 0 (0 to 0) | 0 (0 to 0) | 0 (0 to 0) | 0 (0 to 0) | -5.33 (-5.45 to -5.2) |
| Bhutan | 0 (0 to 0) | 0.5 (0.2 to 1.1) | 0 (0 to 0) | 0.2 (0.1 to 0.5) | -3.05 (-3.13 to -2.98) |
| Bolivia (Plurinational State of) | 0 (0 to 0) | 0.1 (0 to 0.3) | 0 (0 to 0) | 0 (0 to 0.1) | -3.35 (-3.5 to -3.19) |
| Bosnia and Herzegovina | 0 (0 to 0) | 0 (0 to 0.1) | 0 (0 to 0) | 0 (0 to 0) | -4.99 (-5.31 to -4.66) |
| Botswana | 0 (0 to 0) | 0.1 (0 to 0.2) | 0 (0 to 0) | 0 (0 to 0.1) | -4.36 (-4.53 to -4.2) |
| Brazil | 0 (0 to 0.1) | 0 (0 to 0.1) | 0 (0 to 0.1) | 0 (0 to 0) | -4.03 (-4.18 to -3.89) |
| Brunei Darussalam | 0 (0 to 0) | 0 (0 to 0.1) | 0 (0 to 0) | 0 (0 to 0) | -3.03 (-3.22 to -2.84) |
| Bulgaria | 0 (0 to 0) | 0.1 (0 to 0.1) | 0 (0 to 0) | 0 (0 to 0) | -5.18 (-5.42 to -4.94) |
| Burkina Faso | 0 (0 to 0) | 0.1 (0 to 0.2) | 0 (0 to 0) | 0.1 (0 to 0.1) | -0.65 (-0.78 to -0.51) |
| Burundi | 0 (0 to 0) | 0.1 (0 to 0.2) | 0 (0 to 0) | 0 (0 to 0.1) | -2.5 (-2.58 to -2.42) |
| Cabo Verde | 0 (0 to 0) | 0.1 (0 to 0.1) | 0 (0 to 0) | 0 (0 to 0) | -5.75 (-6.62 to -4.88) |
| Cambodia | 0 (0 to 0) | 0.1 (0 to 0.2) | 0 (0 to 0) | 0 (0 to 0.1) | -3.83 (-3.91 to -3.75) |
| Cameroon | 0 (0 to 0) | 0.1 (0 to 0.2) | 0 (0 to 0) | 0 (0 to 0.1) | -2.64 (-2.76 to -2.52) |
| Canada | 0 (0 to 0) | 0 (0 to 0) | 0 (0 to 0) | 0 (0 to 0) | -3.13 (-3.39 to -2.86) |
| Central African Republic | 0 (0 to 0) | 0.2 (0.1 to 0.4) | 0 (0 to 0) | 0.1 (0 to 0.3) | -0.79 (-0.88 to -0.71) |
| Chad | 0 (0 to 0) | 0.1 (0.1 to 0.3) | 0 (0 to 0) | 0.1 (0 to 0.2) | -1.37 (-1.47 to -1.28) |
| Chile | 0 (0 to 0) | 0 (0 to 0) | 0 (0 to 0) | 0 (0 to 0) | -6.23 (-6.48 to -5.97) |
| China | 3.6 (1.9 to 6.4) | 0.5 (0.2 to 0.9) | 1.7 (0.8 to 3.2) | 0.1 (0 to 0.2) | -5.22 (-5.52 to -4.91) |
| Colombia | 0 (0 to 0) | 0 (0 to 0.1) | 0 (0 to 0) | 0 (0 to 0) | -7.14 (-7.69 to -6.6) |
| Comoros | 0 (0 to 0) | 0.1 (0 to 0.1) | 0 (0 to 0) | 0 (0 to 0.1) | -3.04 (-3.21 to -2.87) |
| Congo | 0 (0 to 0) | 0.1 (0 to 0.2) | 0 (0 to 0) | 0 (0 to 0.1) | -3.45 (-3.75 to -3.15) |
| Cook Islands | 0 (0 to 0) | 0 (0 to 0) | 0 (0 to 0) | 0 (0 to 0) | -4.15 (-4.32 to -3.97) |
| Costa Rica | 0 (0 to 0) | 0 (0 to 0.1) | 0 (0 to 0) | 0 (0 to 0) | -4.09 (-4.5 to -3.67) |
| Croatia | 0 (0 to 0) | 0 (0 to 0.1) | 0 (0 to 0) | 0 (0 to 0) | -3.99 (-5.8 to -2.13) |
| Cuba | 0 (0 to 0) | 0 (0 to 0.1) | 0 (0 to 0) | 0 (0 to 0) | -3.32 (-4.01 to -2.62) |
| Cyprus | 0 (0 to 0) | 0.1 (0 to 0.2) | 0 (0 to 0) | 0 (0 to 0.1) | -3.75 (-4.17 to -3.32) |
| Czechia | 0 (0 to 0) | 0 (0 to 0.1) | 0 (0 to 0) | 0 (0 to 0) | -5.09 (-5.42 to -4.76) |
| Côte d'Ivoire | 0 (0 to 0) | 0.1 (0 to 0.1) | 0 (0 to 0) | 0 (0 to 0.1) | -2.71 (-2.89 to -2.53) |
| Democratic People's Republic of Korea | 0 (0 to 0) | 0.1 (0 to 0.3) | 0 (0 to 0.1) | 0.1 (0 to 0.2) | -0.76 (-0.84 to -0.68) |
| Democratic Republic of the Congo | 0 (0 to 0) | 0.1 (0 to 0.2) | 0 (0 to 0.1) | 0.1 (0 to 0.2) | -1.01 (-1.24 to -0.79) |
| Denmark | 0 (0 to 0) | 0 (0 to 0) | 0 (0 to 0) | 0 (0 to 0) | -5.29 (-6.29 to -4.27) |
| Djibouti | 0 (0 to 0) | 0.1 (0 to 0.1) | 0 (0 to 0) | 0 (0 to 0) | -2.95 (-3.06 to -2.84) |
| Dominica | 0 (0 to 0) | 0 (0 to 0.1) | 0 (0 to 0) | 0 (0 to 0) | -3.9 (-4.01 to -3.79) |
| Dominican Republic | 0 (0 to 0) | 0.1 (0 to 0.1) | 0 (0 to 0) | 0 (0 to 0.1) | -2.41 (-3.02 to -1.8) |
| Ecuador | 0 (0 to 0) | 0 (0 to 0.1) | 0 (0 to 0) | 0 (0 to 0) | -4.16 (-4.62 to -3.7) |
| Egypt | 0 (0 to 0.1) | 0.1 (0.1 to 0.3) | 0 (0 to 0.1) | 0 (0 to 0.1) | -3.42 (-3.68 to -3.15) |
| El Salvador | 0 (0 to 0) | 0.1 (0 to 0.1) | 0 (0 to 0) | 0 (0 to 0) | -3.55 (-4.03 to -3.07) |
| Equatorial Guinea | 0 (0 to 0) | 0.2 (0.1 to 0.5) | 0 (0 to 0) | 0 (0 to 0.1) | -6.16 (-6.33 to -6) |
| Eritrea | 0 (0 to 0) | 0.1 (0 to 0.3) | 0 (0 to 0) | 0 (0 to 0.1) | -2.89 (-2.94 to -2.83) |
| Estonia | 0 (0 to 0) | 0 (0 to 0) | 0 (0 to 0) | 0 (0 to 0) | -5.28 (-6.02 to -4.54) |
| Eswatini | 0 (0 to 0) | 0.1 (0 to 0.2) | 0 (0 to 0) | 0 (0 to 0.1) | -3.04 (-3.34 to -2.74) |
| Ethiopia | 0 (0 to 0.1) | 0.2 (0.1 to 0.3) | 0 (0 to 0) | 0.1 (0 to 0.1) | -3.54 (-3.63 to -3.44) |
| Fiji | 0 (0 to 0) | 0 (0 to 0.1) | 0 (0 to 0) | 0 (0 to 0.1) | -3.12 (-3.46 to -2.78) |
| Finland | 0 (0 to 0) | 0 (0 to 0) | 0 (0 to 0) | 0 (0 to 0) | -6.2 (-6.48 to -5.91) |
| France | 0 (0 to 0.1) | 0 (0 to 0.1) | 0 (0 to 0.1) | 0 (0 to 0) | -2.62 (-2.87 to -2.36) |
| Gabon | 0 (0 to 0) | 0 (0 to 0.1) | 0 (0 to 0) | 0 (0 to 0) | -3.87 (-4.02 to -3.73) |
| Gambia | 0 (0 to 0) | 0.1 (0 to 0.2) | 0 (0 to 0) | 0 (0 to 0.1) | -2.13 (-2.78 to -1.47) |
| Georgia | 0 (0 to 0) | 0 (0 to 0.1) | 0 (0 to 0) | 0 (0 to 0.1) | 2.47 (1.92 to 3.01) |
| Germany | 0 (0 to 0.1) | 0 (0 to 0) | 0 (0 to 0.1) | 0 (0 to 0) | -1.77 (-2.01 to -1.52) |
| Ghana | 0 (0 to 0) | 0 (0 to 0.1) | 0 (0 to 0) | 0 (0 to 0) | -2.66 (-2.82 to -2.51) |
| Greece | 0 (0 to 0) | 0 (0 to 0) | 0 (0 to 0) | 0 (0 to 0) | -3.46 (-4.14 to -2.77) |
| Greenland | 0 (0 to 0) | 0.1 (0 to 0.1) | 0 (0 to 0) | 0 (0 to 0) | -4.2 (-4.99 to -3.4) |
| Grenada | 0 (0 to 0) | 0.1 (0.1 to 0.2) | 0 (0 to 0) | 0 (0 to 0.1) | -4.6 (-4.98 to -4.21) |
| Guam | 0 (0 to 0) | 0 (0 to 0) | 0 (0 to 0) | 0 (0 to 0) | -5.29 (-5.81 to -4.77) |
| Guatemala | 0 (0 to 0) | 0.1 (0 to 0.1) | 0 (0 to 0) | 0 (0 to 0) | -3.79 (-4.49 to -3.1) |
| Guinea | 0 (0 to 0) | 0.1 (0.1 to 0.3) | 0 (0 to 0) | 0.1 (0 to 0.2) | -2.1 (-2.17 to -2.02) |
| Guinea-Bissau | 0 (0 to 0) | 0.2 (0.1 to 0.4) | 0 (0 to 0) | 0.1 (0 to 0.2) | -2.9 (-3.03 to -2.77) |
| Guyana | 0 (0 to 0) | 0.1 (0 to 0.2) | 0 (0 to 0) | 0 (0 to 0.1) | -3.51 (-3.85 to -3.18) |
| Haiti | 0 (0 to 0) | 0.5 (0.2 to 1.1) | 0 (0 to 0) | 0.2 (0.1 to 0.5) | -2.89 (-3 to -2.78) |
| Honduras | 0 (0 to 0) | 0 (0 to 0.1) | 0 (0 to 0) | 0 (0 to 0.1) | -1.34 (-1.91 to -0.76) |
| Hungary | 0 (0 to 0) | 0 (0 to 0.1) | 0 (0 to 0) | 0 (0 to 0) | -5.68 (-6.16 to -5.19) |
| Iceland | 0 (0 to 0) | 0 (0 to 0) | 0 (0 to 0) | 0 (0 to 0) | -3.41 (-3.64 to -3.18) |
| India | 4.1 (2.3 to 6.8) | 0.9 (0.5 to 1.5) | 4.5 (2.5 to 7.6) | 0.4 (0.2 to 0.8) | -2.44 (-2.62 to -2.27) |
| Indonesia | 0 (0 to 0.1) | 0 (0 to 0.1) | 0 (0 to 0) | 0 (0 to 0) | -4.97 (-5.15 to -4.79) |
| Iran (Islamic Republic of) | 0 (0 to 0.1) | 0.1 (0.1 to 0.2) | 0 (0 to 0.1) | 0 (0 to 0.1) | -3.29 (-3.69 to -2.88) |
| Iraq | 0 (0 to 0) | 0.1 (0 to 0.2) | 0 (0 to 0) | 0 (0 to 0.1) | -4.63 (-5.14 to -4.12) |
| Ireland | 0 (0 to 0) | 0 (0 to 0) | 0 (0 to 0) | 0 (0 to 0) | -4.13 (-4.7 to -3.56) |
| Israel | 0 (0 to 0) | 0 (0 to 0) | 0 (0 to 0) | 0 (0 to 0) | -2.56 (-2.95 to -2.17) |
| Italy | 0 (0 to 0.1) | 0 (0 to 0.1) | 0 (0 to 0.1) | 0 (0 to 0) | -2.98 (-3.33 to -2.63) |
| Jamaica | 0 (0 to 0) | 0 (0 to 0.1) | 0 (0 to 0) | 0 (0 to 0) | -4.29 (-5.08 to -3.49) |
| Japan | 0 (0 to 0) | 0 (0 to 0) | 0 (0 to 0.1) | 0 (0 to 0) | -3.64 (-3.81 to -3.47) |
| Jordan | 0 (0 to 0) | 0 (0 to 0.1) | 0 (0 to 0) | 0 (0 to 0) | -5.04 (-5.37 to -4.71) |
| Kazakhstan | 0 (0 to 0) | 0.1 (0 to 0.1) | 0 (0 to 0) | 0 (0 to 0) | -5.01 (-5.36 to -4.66) |
| Kenya | 0 (0 to 0) | 0 (0 to 0.1) | 0 (0 to 0) | 0 (0 to 0) | -2.18 (-2.25 to -2.1) |
| Kiribati | 0 (0 to 0) | 0.2 (0 to 0.5) | 0 (0 to 0) | 0.1 (0 to 0.3) | -1.5 (-1.56 to -1.44) |
| Kuwait | 0 (0 to 0) | 0 (0 to 0) | 0 (0 to 0) | 0 (0 to 0) | -5.4 (-6.39 to -4.39) |
| Kyrgyzstan | 0 (0 to 0) | 0.1 (0 to 0.2) | 0 (0 to 0) | 0 (0 to 0.1) | -3.57 (-4.43 to -2.69) |
| Lao People's Democratic Republic | 0 (0 to 0) | 0.1 (0 to 0.2) | 0 (0 to 0) | 0 (0 to 0.1) | -3.48 (-3.59 to -3.37) |
| Latvia | 0 (0 to 0) | 0 (0 to 0.1) | 0 (0 to 0) | 0 (0 to 0) | -6.38 (-8.45 to -4.27) |
| Lebanon | 0 (0 to 0) | 0 (0 to 0.1) | 0 (0 to 0) | 0 (0 to 0) | -4.99 (-5.12 to -4.86) |
| Lesotho | 0 (0 to 0) | 0.1 (0.1 to 0.3) | 0 (0 to 0) | 0.1 (0 to 0.2) | -1.61 (-1.7 to -1.53) |
| Liberia | 0 (0 to 0) | 0.1 (0 to 0.2) | 0 (0 to 0) | 0 (0 to 0.1) | -2.16 (-2.36 to -1.97) |
| Libya | 0 (0 to 0) | 0 (0 to 0.1) | 0 (0 to 0) | 0 (0 to 0) | -3.97 (-4.36 to -3.58) |
| Lithuania | 0 (0 to 0) | 0 (0 to 0.1) | 0 (0 to 0) | 0 (0 to 0) | -5.92 (-6.86 to -4.97) |
| Luxembourg | 0 (0 to 0) | 0 (0 to 0) | 0 (0 to 0) | 0 (0 to 0) | -3.2 (-3.46 to -2.95) |
| Madagascar | 0 (0 to 0) | 0.1 (0 to 0.2) | 0 (0 to 0) | 0.1 (0 to 0.1) | -1.72 (-1.93 to -1.51) |
| Malawi | 0 (0 to 0) | 0.1 (0 to 0.1) | 0 (0 to 0) | 0 (0 to 0.1) | -2.28 (-2.45 to -2.12) |
| Malaysia | 0 (0 to 0) | 0 (0 to 0.1) | 0 (0 to 0) | 0 (0 to 0) | -6 (-6.45 to -5.55) |
| Maldives | 0 (0 to 0) | 0.1 (0 to 0.1) | 0 (0 to 0) | 0 (0 to 0) | -5.55 (-5.73 to -5.36) |
| Mali | 0 (0 to 0) | 0.2 (0.1 to 0.3) | 0 (0 to 0) | 0.1 (0 to 0.2) | -2.5 (-2.71 to -2.3) |
| Malta | 0 (0 to 0) | 0.1 (0 to 0.1) | 0 (0 to 0) | 0 (0 to 0) | -2.89 (-3.05 to -2.74) |
| Marshall Islands | 0 (0 to 0) | 0.1 (0 to 0.4) | 0 (0 to 0) | 0.1 (0 to 0.2) | -2.87 (-3.15 to -2.59) |
| Mauritania | 0 (0 to 0) | 0.1 (0 to 0.2) | 0 (0 to 0) | 0 (0 to 0) | -4.22 (-4.43 to -4.02) |
| Mauritius | 0 (0 to 0) | 0 (0 to 0.1) | 0 (0 to 0) | 0 (0 to 0) | -6 (-6.42 to -5.59) |
| Mexico | 0 (0 to 0.1) | 0.1 (0 to 0.2) | 0 (0 to 0) | 0 (0 to 0) | -5.65 (-6.07 to -5.23) |
| Micronesia (Federated States of) | 0 (0 to 0) | 0.2 (0 to 0.5) | 0 (0 to 0) | 0.1 (0 to 0.2) | -3.39 (-3.46 to -3.32) |
| Monaco | 0 (0 to 0) | 0 (0 to 0) | 0 (0 to 0) | 0 (0 to 0) | -2.06 (-2.12 to -2) |
| Mongolia | 0 (0 to 0) | 0.2 (0.1 to 0.5) | 0 (0 to 0) | 0.1 (0 to 0.1) | -3.56 (-3.84 to -3.28) |
| Montenegro | 0 (0 to 0) | 0 (0 to 0) | 0 (0 to 0) | 0 (0 to 0) | -1.06 (-1.26 to -0.86) |
| Morocco | 0 (0 to 0) | 0.1 (0 to 0.2) | 0 (0 to 0) | 0 (0 to 0.1) | -3.4 (-3.7 to -3.09) |
| Mozambique | 0 (0 to 0) | 0.1 (0 to 0.2) | 0 (0 to 0) | 0.1 (0 to 0.1) | -1.67 (-1.76 to -1.57) |
| Myanmar | 0 (0 to 0.1) | 0.1 (0 to 0.2) | 0 (0 to 0) | 0 (0 to 0.1) | -3.78 (-3.9 to -3.67) |
| Namibia | 0 (0 to 0) | 0.1 (0 to 0.2) | 0 (0 to 0) | 0 (0 to 0.1) | -3.62 (-3.72 to -3.52) |
| Nauru | 0 (0 to 0) | 0 (0 to 0.1) | 0 (0 to 0) | 0 (0 to 0.1) | -1.76 (-1.92 to -1.61) |
| Nepal | 0.1 (0 to 0.1) | 0.7 (0.3 to 1.5) | 0.1 (0 to 0.2) | 0.4 (0.2 to 0.8) | -2 (-2.08 to -1.93) |
| Netherlands | 0 (0 to 0) | 0 (0 to 0) | 0 (0 to 0) | 0 (0 to 0) | -1.3 (-2.24 to -0.35) |
| New Zealand | 0 (0 to 0) | 0.1 (0 to 0.1) | 0 (0 to 0) | 0 (0 to 0) | -3.54 (-3.9 to -3.18) |
| Nicaragua | 0 (0 to 0) | 0 (0 to 0.1) | 0 (0 to 0) | 0 (0 to 0) | -4.2 (-4.71 to -3.69) |
| Niger | 0 (0 to 0) | 0.2 (0.1 to 0.4) | 0 (0 to 0) | 0.1 (0 to 0.3) | -1.85 (-2.04 to -1.65) |
| Nigeria | 0 (0 to 0.1) | 0.1 (0 to 0.2) | 0 (0 to 0.1) | 0 (0 to 0.1) | -3.78 (-3.95 to -3.6) |
| Niue | 0 (0 to 0) | 0 (0 to 0) | 0 (0 to 0) | 0 (0 to 0) | -3.7 (-3.82 to -3.58) |
| North Macedonia | 0 (0 to 0) | 0 (0 to 0.1) | 0 (0 to 0) | 0 (0 to 0) | -3.76 (-4 to -3.53) |
| Northern Mariana Islands | 0 (0 to 0) | 0 (0 to 0) | 0 (0 to 0) | 0 (0 to 0) | -3.3 (-3.44 to -3.17) |
| Norway | 0 (0 to 0) | 0 (0 to 0) | 0 (0 to 0) | 0 (0 to 0) | -3.56 (-3.99 to -3.13) |
| Oman | 0 (0 to 0) | 0 (0 to 0.1) | 0 (0 to 0) | 0 (0 to 0) | -4.75 (-5.31 to -4.19) |
| Pakistan | 0.3 (0.2 to 0.6) | 0.6 (0.3 to 1.1) | 0.4 (0.2 to 0.7) | 0.4 (0.2 to 0.7) | -1.37 (-1.49 to -1.25) |
| Palau | 0 (0 to 0) | 0 (0 to 0) | 0 (0 to 0) | 0 (0 to 0) | -3.54 (-3.6 to -3.48) |
| Palestine | 0 (0 to 0) | 0 (0 to 0.1) | 0 (0 to 0) | 0 (0 to 0) | -4.2 (-4.39 to -4.01) |
| Panama | 0 (0 to 0) | 0 (0 to 0.1) | 0 (0 to 0) | 0 (0 to 0) | -5.43 (-6.11 to -4.73) |
| Papua New Guinea | 0 (0 to 0) | 0.1 (0 to 0.3) | 0 (0 to 0) | 0.1 (0 to 0.2) | -0.93 (-1 to -0.86) |
| Paraguay | 0 (0 to 0) | 0 (0 to 0.1) | 0 (0 to 0) | 0 (0 to 0) | -2.44 (-2.92 to -1.95) |
| Peru | 0 (0 to 0) | 0 (0 to 0.1) | 0 (0 to 0) | 0 (0 to 0) | -4.15 (-5.01 to -3.28) |
| Philippines | 0 (0 to 0) | 0 (0 to 0) | 0 (0 to 0) | 0 (0 to 0) | -0.16 (-0.49 to 0.18) |
| Poland | 0 (0 to 0.1) | 0.1 (0 to 0.2) | 0 (0 to 0) | 0 (0 to 0) | -6.49 (-6.75 to -6.22) |
| Portugal | 0 (0 to 0) | 0.1 (0 to 0.1) | 0 (0 to 0) | 0 (0 to 0) | -4.34 (-4.76 to -3.92) |
| Puerto Rico | 0 (0 to 0) | 0 (0 to 0) | 0 (0 to 0) | 0 (0 to 0) | -4.65 (-4.96 to -4.35) |
| Qatar | 0 (0 to 0) | 0 (0 to 0.1) | 0 (0 to 0) | 0 (0 to 0) | -4.18 (-4.72 to -3.63) |
| Republic of Korea | 0 (0 to 0) | 0 (0 to 0) | 0 (0 to 0) | 0 (0 to 0) | -4.44 (-4.65 to -4.23) |
| Republic of Moldova | 0 (0 to 0) | 0 (0 to 0.1) | 0 (0 to 0) | 0 (0 to 0) | -5 (-5.82 to -4.18) |
| Romania | 0 (0 to 0) | 0.1 (0 to 0.1) | 0 (0 to 0) | 0 (0 to 0) | -5.66 (-6.11 to -5.22) |
| Russian Federation | 0 (0 to 0.1) | 0 (0 to 0.1) | 0 (0 to 0) | 0 (0 to 0) | -5.08 (-5.97 to -4.18) |
| Rwanda | 0 (0 to 0) | 0.1 (0 to 0.2) | 0 (0 to 0) | 0 (0 to 0.1) | -4.17 (-4.28 to -4.06) |
| Saint Kitts and Nevis | 0 (0 to 0) | 0 (0 to 0.1) | 0 (0 to 0) | 0 (0 to 0) | -5.6 (-6.02 to -5.18) |
| Saint Lucia | 0 (0 to 0) | 0.1 (0 to 0.2) | 0 (0 to 0) | 0 (0 to 0) | -5.21 (-5.42 to -4.99) |
| Saint Vincent and the Grenadines | 0 (0 to 0) | 0.1 (0 to 0.2) | 0 (0 to 0) | 0 (0 to 0.1) | -4.12 (-4.44 to -3.81) |
| Samoa | 0 (0 to 0) | 0 (0 to 0.1) | 0 (0 to 0) | 0 (0 to 0.1) | -2.42 (-2.62 to -2.22) |
| San Marino | 0 (0 to 0) | 0 (0 to 0.1) | 0 (0 to 0) | 0 (0 to 0) | -1.63 (-1.73 to -1.52) |
| Sao Tome and Principe | 0 (0 to 0) | 0.1 (0 to 0.2) | 0 (0 to 0) | 0.1 (0 to 0.1) | -1.82 (-2.04 to -1.6) |
| Saudi Arabia | 0 (0 to 0) | 0.1 (0 to 0.2) | 0 (0 to 0) | 0 (0 to 0) | -5.44 (-5.75 to -5.14) |
| Senegal | 0 (0 to 0) | 0.1 (0 to 0.1) | 0 (0 to 0) | 0 (0 to 0.1) | -2.79 (-3 to -2.57) |
| Serbia | 0 (0 to 0) | 0 (0 to 0.1) | 0 (0 to 0) | 0 (0 to 0) | -2.67 (-3.11 to -2.22) |
| Seychelles | 0 (0 to 0) | 0 (0 to 0.1) | 0 (0 to 0) | 0 (0 to 0) | -7.11 (-7.4 to -6.81) |
| Sierra Leone | 0 (0 to 0) | 0.1 (0 to 0.2) | 0 (0 to 0) | 0 (0 to 0.1) | -2.05 (-2.16 to -1.94) |
| Singapore | 0 (0 to 0) | 0 (0 to 0.1) | 0 (0 to 0) | 0 (0 to 0) | -7.33 (-7.49 to -7.17) |
| Slovakia | 0 (0 to 0) | 0 (0 to 0) | 0 (0 to 0) | 0 (0 to 0) | -3.06 (-3.61 to -2.51) |
| Slovenia | 0 (0 to 0) | 0 (0 to 0.1) | 0 (0 to 0) | 0 (0 to 0) | -2.81 (-3.14 to -2.47) |
| Solomon Islands | 0 (0 to 0) | 0.2 (0 to 0.6) | 0 (0 to 0) | 0.1 (0 to 0.4) | -1.7 (-1.83 to -1.57) |
| Somalia | 0 (0 to 0) | 0.2 (0.1 to 0.4) | 0 (0 to 0) | 0.1 (0 to 0.3) | -1.47 (-1.53 to -1.41) |
| South Africa | 0 (0 to 0) | 0 (0 to 0.1) | 0 (0 to 0) | 0 (0 to 0) | -2.58 (-3.12 to -2.04) |
| South Sudan | 0 (0 to 0) | 0.1 (0 to 0.1) | 0 (0 to 0) | 0 (0 to 0.1) | -1.89 (-1.95 to -1.82) |
| Spain | 0 (0 to 0.1) | 0.1 (0 to 0.1) | 0 (0 to 0.1) | 0 (0 to 0.1) | -3.18 (-3.37 to -2.98) |
| Sri Lanka | 0 (0 to 0) | 0 (0 to 0) | 0 (0 to 0) | 0 (0 to 0) | -4.48 (-5.09 to -3.86) |
| Sudan | 0 (0 to 0.1) | 0.3 (0.1 to 0.7) | 0 (0 to 0) | 0.1 (0 to 0.3) | -3.45 (-3.53 to -3.37) |
| Suriname | 0 (0 to 0) | 0 (0 to 0.1) | 0 (0 to 0) | 0 (0 to 0) | -4.09 (-4.89 to -3.28) |
| Sweden | 0 (0 to 0) | 0 (0 to 0) | 0 (0 to 0) | 0 (0 to 0) | -3 (-3.17 to -2.83) |
| Switzerland | 0 (0 to 0) | 0 (0 to 0) | 0 (0 to 0) | 0 (0 to 0) | -5.03 (-5.25 to -4.81) |
| Syrian Arab Republic | 0 (0 to 0) | 0.3 (0.1 to 0.5) | 0 (0 to 0) | 0 (0 to 0.1) | -6.85 (-7.25 to -6.44) |
| Taiwan (Province of China) | 0 (0 to 0) | 0 (0 to 0.1) | 0 (0 to 0) | 0 (0 to 0) | -7.13 (-7.45 to -6.82) |
| Tajikistan | 0 (0 to 0) | 0.2 (0.1 to 0.3) | 0 (0 to 0) | 0.1 (0 to 0.1) | -3.15 (-3.38 to -2.92) |
| Thailand | 0 (0 to 0) | 0 (0 to 0.1) | 0 (0 to 0) | 0 (0 to 0) | -8.9 (-9.33 to -8.46) |
| Timor-Leste | 0 (0 to 0) | 0.1 (0 to 0.2) | 0 (0 to 0) | 0 (0 to 0.1) | -2.23 (-2.54 to -1.91) |
| Togo | 0 (0 to 0) | 0.1 (0 to 0.2) | 0 (0 to 0) | 0 (0 to 0.1) | -2.49 (-2.66 to -2.32) |
| Tokelau | 0 (0 to 0) | 0.1 (0 to 0.2) | 0 (0 to 0) | 0 (0 to 0.1) | -4.19 (-4.3 to -4.09) |
| Tonga | 0 (0 to 0) | 0 (0 to 0.1) | 0 (0 to 0) | 0 (0 to 0) | -3.05 (-3.3 to -2.79) |
| Trinidad and Tobago | 0 (0 to 0) | 0 (0 to 0) | 0 (0 to 0) | 0 (0 to 0) | -5.55 (-6.1 to -5) |
| Tunisia | 0 (0 to 0) | 0.1 (0 to 0.1) | 0 (0 to 0) | 0 (0 to 0) | -3.77 (-3.84 to -3.69) |
| Turkey | NA | NA | NA | NA | NA |
| Turkmenistan | 0 (0 to 0) | 0 (0 to 0.1) | 0 (0 to 0) | 0 (0 to 0) | -3.8 (-5.02 to -2.57) |
| Tuvalu | 0 (0 to 0) | 0.1 (0 to 0.2) | 0 (0 to 0) | 0 (0 to 0.1) | -3.39 (-3.5 to -3.29) |
| Uganda | 0 (0 to 0) | 0.1 (0 to 0.1) | 0 (0 to 0) | 0 (0 to 0.1) | -2.69 (-2.88 to -2.51) |
| Ukraine | 0 (0 to 0) | 0 (0 to 0) | 0 (0 to 0) | 0 (0 to 0) | -2.36 (-3.16 to -1.55) |
| United Arab Emirates | 0 (0 to 0) | 0.1 (0 to 0.3) | 0 (0 to 0) | 0 (0 to 0.1) | -5.07 (-5.83 to -4.3) |
| United Kingdom | 0 (0 to 0) | 0 (0 to 0) | 0 (0 to 0) | 0 (0 to 0) | -4.83 (-5.07 to -4.59) |
| United Republic of Tanzania | 0 (0 to 0) | 0.1 (0 to 0.1) | 0 (0 to 0) | 0 (0 to 0.1) | -2.91 (-3.03 to -2.78) |
| United States of America | 0.1 (0 to 0.2) | 0 (0 to 0.1) | 0 (0 to 0.1) | 0 (0 to 0) | -4.15 (-4.31 to -3.98) |
| United States Virgin Islands | 0 (0 to 0) | 0 (0 to 0) | 0 (0 to 0) | 0 (0 to 0) | -3.77 (-4.09 to -3.44) |
| Uruguay | 0 (0 to 0) | 0 (0 to 0) | 0 (0 to 0) | 0 (0 to 0) | -1.69 (-2.06 to -1.31) |
| Uzbekistan | 0 (0 to 0) | 0.1 (0 to 0.2) | 0 (0 to 0) | 0 (0 to 0.1) | -1.61 (-2.52 to -0.7) |
| Vanuatu | 0 (0 to 0) | 0.1 (0 to 0.3) | 0 (0 to 0) | 0.1 (0 to 0.2) | -1.45 (-1.61 to -1.28) |
| Venezuela (Bolivarian Republic of) | 0 (0 to 0) | 0 (0 to 0.1) | 0 (0 to 0) | 0 (0 to 0) | -4.27 (-4.53 to -4.01) |
| Viet Nam | 0 (0 to 0) | 0 (0 to 0.1) | 0 (0 to 0) | 0 (0 to 0) | -3.74 (-3.82 to -3.65) |
| Yemen | 0 (0 to 0) | 0.4 (0.2 to 0.9) | 0 (0 to 0.1) | 0.2 (0.1 to 0.4) | -2.28 (-2.47 to -2.09) |
| Zambia | 0 (0 to 0) | 0.1 (0 to 0.1) | 0 (0 to 0) | 0 (0 to 0.1) | -2.03 (-2.31 to -1.76) |
| Zimbabwe | 0 (0 to 0) | 0.1 (0.1 to 0.2) | 0 (0 to 0) | 0.1 (0 to 0.2) | -0.55 (-0.68 to -0.41) |
