# Supplementary material for: Spatiotemporal patterns of rheumatic heart disease burden attributable to high systolic blood pressure, high sodium diet, and lead exposure (1990 to 2019): a longitudinal observational study
Source: Front Nutr. 2024 Sep 26;11:1419349. doi: 10.3389/fnut.2024.1419349 (PMC11466049; doi:10.3389/fnut.2024.1419349)
Supplement: Supplementary file 6 [file Table_6.docx]

**Supplementary table 6. Disability-adjusted life year of rheumatic heart disease due to lead exposure**

| **location** | **1990 Counts**  **(thousand)** | **Age-standardised rate (per 100 000 population), 1990** | **2019 Counts**  **(thousand)** | **Age-standardised rate (per 100 000 population), 2019** | **Average annual percent change** |
| --- | --- | --- | --- | --- | --- |
| Afghanistan | 1.3 (0.6 to 2.8) | 18.1 (7.9 to 36.9) | 1.9 (0.9 to 3.8) | 9.9 (4.8 to 19.4) | -2.04 (-2.17 to -1.92) |
| Albania | 0 (0 to 0.1) | 2 (0.6 to 4.1) | 0 (0 to 0) | 0.5 (0.1 to 1.2) | -4.62 (-5.14 to -4.1) |
| Algeria | 0.3 (0.1 to 0.6) | 1.9 (0.6 to 3.9) | 0.2 (0 to 0.4) | 0.4 (0.1 to 1) | -4.81 (-4.87 to -4.76) |
| American Samoa | 0 (0 to 0) | 0.3 (0 to 1.5) | 0 (0 to 0) | 0.1 (0 to 0.9) | -2.71 (-3.09 to -2.33) |
| Andorra | 0 (0 to 0) | 0.2 (0 to 0.5) | 0 (0 to 0) | 0.1 (0 to 0.2) | -2.67 (-2.95 to -2.39) |
| Angola | 0.2 (0.1 to 0.5) | 3.7 (1.2 to 7.6) | 0.2 (0.1 to 0.6) | 1.6 (0.5 to 3.3) | -2.86 (-3.06 to -2.66) |
| Antigua and Barbuda | 0 (0 to 0) | 0.9 (0.1 to 2.3) | 0 (0 to 0) | 0.3 (0 to 0.9) | -3.99 (-4.11 to -3.88) |
| Argentina | 0.3 (0 to 0.8) | 0.9 (0 to 2.4) | 0.2 (0 to 0.5) | 0.3 (0 to 0.9) | -3.51 (-3.75 to -3.27) |
| Armenia | 0.1 (0 to 0.2) | 2.2 (0.3 to 5.3) | 0 (0 to 0.1) | 0.7 (0.1 to 1.7) | -3.96 (-4.59 to -3.33) |
| Australia | 0.1 (0 to 0.3) | 0.7 (0.2 to 1.4) | 0.1 (0 to 0.2) | 0.2 (0.1 to 0.5) | -4.2 (-4.51 to -3.89) |
| Austria | 0 (0 to 0.1) | 0.4 (0 to 0.9) | 0 (0 to 0.1) | 0.1 (0 to 0.3) | -4.19 (-4.43 to -3.95) |
| Azerbaijan | 0.1 (0 to 0.2) | 1 (0.1 to 2.7) | 0 (0 to 0.1) | 0.4 (0 to 1.2) | -3 (-3.24 to -2.76) |
| Bahamas | 0 (0 to 0) | 0.5 (0 to 1.6) | 0 (0 to 0) | 0.2 (0 to 0.7) | -3.67 (-3.87 to -3.48) |
| Bahrain | 0 (0 to 0) | 0.6 (0.2 to 1.3) | 0 (0 to 0) | 0.2 (0 to 0.4) | -4.16 (-4.48 to -3.84) |
| Bangladesh | 4.8 (2.4 to 8.2) | 6.8 (3.7 to 11.1) | 3.1 (1.3 to 5.7) | 2.2 (1 to 4) | -3.67 (-4.38 to -2.94) |
| Barbados | 0 (0 to 0) | 0.7 (0.1 to 2.1) | 0 (0 to 0) | 0.2 (0 to 0.7) | -4.15 (-4.45 to -3.85) |
| Belarus | 0.2 (0 to 0.5) | 1.4 (0 to 4.1) | 0 (0 to 0.1) | 0.3 (0 to 0.9) | -5.15 (-5.85 to -4.45) |
| Belgium | 0.1 (0 to 0.1) | 0.4 (0.2 to 0.8) | 0.1 (0 to 0.3) | 0.4 (0.2 to 0.9) | -0.12 (-0.34 to 0.09) |
| Belize | 0 (0 to 0) | 1.2 (0.3 to 2.7) | 0 (0 to 0) | 0.4 (0.1 to 1.2) | -3.8 (-4.02 to -3.58) |
| Benin | 0.1 (0 to 0.2) | 3.4 (1.3 to 6.2) | 0.1 (0 to 0.2) | 1.5 (0.6 to 3.1) | -2.67 (-2.76 to -2.57) |
| Bermuda | 0 (0 to 0) | 0.3 (0 to 0.9) | 0 (0 to 0) | 0.1 (0 to 0.2) | -5.87 (-6 to -5.75) |
| Bhutan | 0 (0 to 0.1) | 14.3 (5 to 31.1) | 0 (0 to 0.1) | 4.2 (1.2 to 10.5) | -4.15 (-4.26 to -4.05) |
| Bolivia (Plurinational State of) | 0.2 (0.1 to 0.4) | 4.1 (1.4 to 8.2) | 0.1 (0 to 0.3) | 1.4 (0.4 to 2.9) | -3.68 (-3.78 to -3.57) |
| Bosnia and Herzegovina | 0 (0 to 0.1) | 1 (0.4 to 2) | 0 (0 to 0) | 0.2 (0.1 to 0.5) | -5.33 (-5.68 to -4.97) |
| Botswana | 0 (0 to 0.1) | 3.9 (1.2 to 8.6) | 0 (0 to 0) | 1 (0.2 to 2.2) | -4.75 (-4.86 to -4.63) |
| Brazil | 1.9 (0.5 to 4.4) | 1.6 (0.5 to 3.5) | 1.1 (0.2 to 2.9) | 0.4 (0.1 to 1.2) | -4.37 (-4.5 to -4.24) |
| Brunei Darussalam | 0 (0 to 0) | 0.9 (0.1 to 2.1) | 0 (0 to 0) | 0.3 (0 to 0.8) | -4 (-4.2 to -3.8) |
| Bulgaria | 0.2 (0 to 0.5) | 1.9 (0.2 to 4.5) | 0 (0 to 0.1) | 0.3 (0 to 0.9) | -5.64 (-5.88 to -5.4) |
| Burkina Faso | 0.1 (0.1 to 0.2) | 2.5 (1.1 to 4.9) | 0.2 (0.1 to 0.4) | 1.9 (0.8 to 3.5) | -1.07 (-1.22 to -0.93) |
| Burundi | 0.1 (0 to 0.2) | 2.9 (1 to 5.7) | 0.1 (0 to 0.2) | 1.3 (0.4 to 2.9) | -2.77 (-2.84 to -2.7) |
| Cabo Verde | 0 (0 to 0) | 2 (0.3 to 4.7) | 0 (0 to 0) | 0.4 (0 to 1) | -5.74 (-6.16 to -5.32) |
| Cambodia | 0.3 (0.1 to 0.7) | 4.8 (1.3 to 9.9) | 0.2 (0 to 0.5) | 1.4 (0.3 to 2.9) | -4.21 (-4.3 to -4.12) |
| Cameroon | 0.2 (0.1 to 0.4) | 3.2 (1.2 to 6.3) | 0.2 (0.1 to 0.5) | 1.4 (0.5 to 3.1) | -2.72 (-2.8 to -2.63) |
| Canada | 0.1 (0 to 0.2) | 0.2 (0 to 0.6) | 0.1 (0 to 0.2) | 0.1 (0 to 0.2) | -3.69 (-3.86 to -3.52) |
| Central African Republic | 0.1 (0 to 0.2) | 5.5 (1.9 to 11.4) | 0.1 (0 to 0.2) | 3.8 (1.1 to 8.9) | -1.24 (-1.31 to -1.18) |
| Chad | 0.1 (0.1 to 0.3) | 4.3 (1.8 to 9.3) | 0.2 (0.1 to 0.4) | 2.8 (1.2 to 5.6) | -1.45 (-1.57 to -1.34) |
| Chile | 0 (0 to 0.1) | 0.3 (0 to 1.3) | 0 (0 to 0) | 0 (0 to 0.2) | -6.7 (-7.17 to -6.21) |
| China | 108.1 (55.8 to 188.9) | 11.5 (6 to 20.5) | 36.6 (17.8 to 65.3) | 1.9 (0.9 to 3.4) | -6.19 (-6.47 to -5.9) |
| Colombia | 0.2 (0.1 to 0.4) | 1 (0.4 to 2) | 0.1 (0 to 0.1) | 0.1 (0 to 0.2) | -7.57 (-8.11 to -7.02) |
| Comoros | 0 (0 to 0) | 1.9 (0.5 to 4.1) | 0 (0 to 0) | 0.7 (0.2 to 1.6) | -3.52 (-3.84 to -3.2) |
| Congo | 0 (0 to 0.1) | 2.6 (0.5 to 5.6) | 0 (0 to 0.1) | 0.8 (0.1 to 1.9) | -3.96 (-4.21 to -3.72) |
| Cook Islands | 0 (0 to 0) | 0.1 (0 to 0.7) | 0 (0 to 0) | 0 (0 to 0.2) | -4.21 (-4.62 to -3.8) |
| Costa Rica | 0 (0 to 0.1) | 2.2 (0.8 to 4.3) | 0 (0 to 0.1) | 0.7 (0.2 to 1.6) | -3.85 (-4.11 to -3.59) |
| Croatia | 0 (0 to 0.1) | 0.5 (0 to 1.4) | 0 (0 to 0) | 0.1 (0 to 0.3) | -4.68 (-6.04 to -3.3) |
| Cuba | 0.2 (0.1 to 0.5) | 2.3 (0.7 to 4.7) | 0.1 (0 to 0.3) | 0.7 (0.2 to 1.7) | -3.61 (-4 to -3.23) |
| Cyprus | 0 (0 to 0) | 1.4 (0.4 to 3.3) | 0 (0 to 0) | 0.4 (0.1 to 0.9) | -4.35 (-4.73 to -3.96) |
| Czechia | 0.1 (0 to 0.3) | 1 (0 to 2.5) | 0 (0 to 0.1) | 0.1 (0 to 0.4) | -6.3 (-6.61 to -5.98) |
| Côte d'Ivoire | 0.1 (0 to 0.3) | 2.1 (0.6 to 4.3) | 0.1 (0 to 0.3) | 0.9 (0.2 to 2) | -2.84 (-3.01 to -2.66) |
| Democratic People's Republic of Korea | 0.6 (0.1 to 1.3) | 3.3 (0.7 to 7.3) | 0.8 (0.2 to 1.8) | 2.4 (0.6 to 5.6) | -1.1 (-1.2 to -1) |
| Democratic Republic of the Congo | 0.6 (0.1 to 1.3) | 2.8 (0.7 to 6.1) | 0.9 (0.2 to 2.1) | 1.9 (0.5 to 4.7) | -1.34 (-1.42 to -1.26) |
| Denmark | 0 (0 to 0.1) | 0.3 (0 to 0.9) | 0 (0 to 0) | 0.1 (0 to 0.1) | -6.27 (-7.06 to -5.47) |
| Djibouti | 0 (0 to 0) | 1.6 (0.4 to 3.4) | 0 (0 to 0) | 0.6 (0.1 to 1.5) | -3.31 (-3.43 to -3.19) |
| Dominica | 0 (0 to 0) | 1.7 (0.3 to 3.9) | 0 (0 to 0) | 0.4 (0.1 to 1.3) | -4.53 (-4.71 to -4.35) |
| Dominican Republic | 0.1 (0 to 0.3) | 2.5 (1.1 to 4.7) | 0.1 (0 to 0.2) | 1 (0.3 to 2.1) | -3.15 (-3.59 to -2.71) |
| Ecuador | 0.1 (0 to 0.2) | 1.2 (0.2 to 2.7) | 0.1 (0 to 0.2) | 0.3 (0.1 to 0.9) | -4.34 (-4.68 to -3.99) |
| Egypt | 1.8 (0.8 to 3.7) | 4.5 (2 to 9.2) | 1.1 (0.4 to 2.2) | 1.4 (0.5 to 2.8) | -3.92 (-4.1 to -3.74) |
| El Salvador | 0.1 (0 to 0.2) | 2.5 (1.3 to 4.5) | 0.1 (0 to 0.1) | 0.9 (0.4 to 1.7) | -3.56 (-3.96 to -3.15) |
| Equatorial Guinea | 0 (0 to 0) | 6.6 (2.7 to 13.4) | 0 (0 to 0) | 1 (0.3 to 2.1) | -6.4 (-6.55 to -6.26) |
| Eritrea | 0 (0 to 0.1) | 3.5 (1.2 to 6.8) | 0 (0 to 0.1) | 1.2 (0.4 to 2.8) | -3.5 (-3.56 to -3.44) |
| Estonia | 0 (0 to 0) | 0.3 (0 to 1.5) | 0 (0 to 0) | 0.1 (0 to 0.3) | -5.2 (-5.92 to -4.47) |
| Eswatini | 0 (0 to 0) | 2.9 (0.7 to 6.3) | 0 (0 to 0) | 1.1 (0.3 to 2.8) | -3.25 (-3.52 to -2.98) |
| Ethiopia | 1.2 (0.5 to 2.3) | 4.8 (2 to 8.6) | 0.8 (0.3 to 1.8) | 1.5 (0.6 to 3.1) | -3.84 (-3.93 to -3.76) |
| Fiji | 0 (0 to 0) | 0.8 (0 to 3.7) | 0 (0 to 0) | 0.3 (0 to 1.6) | -3.33 (-3.67 to -2.99) |
| Finland | 0 (0 to 0) | 0.1 (0 to 0.3) | 0 (0 to 0) | 0 (0 to 0) | -6.57 (-6.82 to -6.32) |
| France | 0.5 (0.2 to 1) | 0.6 (0.2 to 1.2) | 0.3 (0.1 to 0.8) | 0.2 (0.1 to 0.5) | -3.39 (-3.71 to -3.07) |
| Gabon | 0 (0 to 0) | 1.6 (0.3 to 3.9) | 0 (0 to 0) | 0.4 (0.1 to 1.2) | -4.48 (-4.59 to -4.37) |
| Gambia | 0 (0 to 0) | 2.5 (0.9 to 5.1) | 0 (0 to 0) | 1.2 (0.4 to 2.6) | -2.38 (-3.03 to -1.73) |
| Georgia | 0 (0 to 0.1) | 0.7 (0 to 2.3) | 0.1 (0 to 0.1) | 0.9 (0.1 to 2.3) | 0.73 (0.4 to 1.07) |
| Germany | 0.4 (0 to 1.1) | 0.3 (0 to 0.9) | 0.3 (0 to 0.8) | 0.1 (0 to 0.4) | -3.17 (-3.42 to -2.92) |
| Ghana | 0.1 (0 to 0.2) | 1 (0.1 to 2.5) | 0.1 (0 to 0.3) | 0.4 (0.1 to 1.2) | -2.89 (-2.97 to -2.81) |
| Greece | 0 (0 to 0.1) | 0.3 (0.1 to 0.6) | 0 (0 to 0.1) | 0.1 (0 to 0.2) | -3.79 (-4.33 to -3.26) |
| Greenland | 0 (0 to 0) | 1.1 (0.2 to 2.4) | 0 (0 to 0) | 0.3 (0.1 to 0.7) | -4.26 (-4.92 to -3.61) |
| Grenada | 0 (0 to 0) | 4.5 (1.6 to 9) | 0 (0 to 0) | 1 (0.3 to 2.2) | -5.14 (-5.48 to -4.81) |
| Guam | 0 (0 to 0) | 0.1 (0 to 0.6) | 0 (0 to 0) | 0 (0 to 0.3) | -4.12 (-4.41 to -3.82) |
| Guatemala | 0.1 (0.1 to 0.3) | 2.9 (1.6 to 4.9) | 0.2 (0.1 to 0.3) | 1.1 (0.5 to 2.2) | -3.15 (-3.44 to -2.86) |
| Guinea | 0.1 (0.1 to 0.3) | 3.8 (1.4 to 7.9) | 0.1 (0 to 0.3) | 2 (0.8 to 4) | -2.17 (-2.29 to -2.05) |
| Guinea-Bissau | 0 (0 to 0.1) | 5.6 (2.1 to 11.2) | 0 (0 to 0) | 2.3 (0.8 to 4.5) | -3.08 (-3.24 to -2.91) |
| Guyana | 0 (0 to 0) | 3.3 (1.1 to 6.5) | 0 (0 to 0) | 1.2 (0.3 to 2.8) | -3.51 (-3.77 to -3.25) |
| Haiti | 0.8 (0.3 to 1.6) | 18.5 (7.9 to 36.5) | 0.7 (0.2 to 1.5) | 7 (2.7 to 15.7) | -3.24 (-3.38 to -3.1) |
| Honduras | 0.1 (0 to 0.1) | 2.4 (1.3 to 4.3) | 0.1 (0 to 0.2) | 1.2 (0.6 to 2.3) | -2.26 (-2.54 to -1.98) |
| Hungary | 0.1 (0 to 0.4) | 1.1 (0 to 2.8) | 0 (0 to 0.1) | 0.1 (0 to 0.4) | -6.49 (-6.85 to -6.12) |
| Iceland | 0 (0 to 0) | 0.1 (0 to 0.3) | 0 (0 to 0) | 0 (0 to 0.1) | -4.15 (-4.4 to -3.89) |
| India | 153.3 (85.3 to 256.5) | 25.9 (14.9 to 42.7) | 132.3 (68.8 to 223) | 10.8 (5.7 to 17.8) | -2.94 (-3.25 to -2.63) |
| Indonesia | 1.9 (0.7 to 3.7) | 1.4 (0.5 to 2.5) | 0.5 (0.2 to 1.2) | 0.2 (0.1 to 0.5) | -5.82 (-6 to -5.64) |
| Iran (Islamic Republic of) | 1.3 (0.7 to 2.4) | 3.8 (2 to 7.3) | 1 (0.5 to 1.8) | 1.2 (0.6 to 2.1) | -3.9 (-4.18 to -3.62) |
| Iraq | 0.3 (0.1 to 0.6) | 3 (1 to 6.1) | 0.2 (0 to 0.5) | 0.7 (0.2 to 1.5) | -4.9 (-5.27 to -4.54) |
| Ireland | 0 (0 to 0) | 0.5 (0.1 to 1) | 0 (0 to 0) | 0.1 (0 to 0.3) | -4.94 (-5.46 to -4.41) |
| Israel | 0 (0 to 0) | 0.4 (0 to 1) | 0 (0 to 0) | 0.1 (0 to 0.4) | -3.69 (-4.05 to -3.33) |
| Italy | 0.7 (0.2 to 1.4) | 0.8 (0.2 to 1.6) | 0.3 (0.1 to 0.8) | 0.2 (0 to 0.5) | -4.38 (-4.76 to -3.99) |
| Jamaica | 0 (0 to 0.1) | 1.6 (0.5 to 3.5) | 0 (0 to 0) | 0.5 (0.1 to 1.5) | -3.87 (-4.3 to -3.44) |
| Japan | 0.3 (0 to 0.8) | 0.2 (0 to 0.5) | 0.2 (0 to 0.6) | 0 (0 to 0.1) | -4.89 (-5.18 to -4.59) |
| Jordan | 0 (0 to 0) | 0.7 (0.2 to 1.4) | 0 (0 to 0) | 0.1 (0 to 0.3) | -5.76 (-5.99 to -5.53) |
| Kazakhstan | 0.3 (0 to 0.7) | 1.8 (0.1 to 5) | 0.1 (0 to 0.2) | 0.3 (0 to 1) | -5.58 (-5.96 to -5.2) |
| Kenya | 0.1 (0 to 0.3) | 1.2 (0.4 to 2.6) | 0.1 (0 to 0.4) | 0.6 (0.1 to 1.3) | -2.71 (-2.78 to -2.65) |
| Kiribati | 0 (0 to 0) | 5 (0.4 to 15.7) | 0 (0 to 0) | 3.1 (0.3 to 10.3) | -1.61 (-1.67 to -1.54) |
| Kuwait | 0 (0 to 0) | 0.7 (0.2 to 1.3) | 0 (0 to 0) | 0.1 (0 to 0.2) | -6.2 (-7.07 to -5.33) |
| Kyrgyzstan | 0.1 (0 to 0.3) | 2.8 (0.2 to 7.6) | 0.1 (0 to 0.2) | 0.9 (0.1 to 2.5) | -3.76 (-4.64 to -2.87) |
| Lao People's Democratic Republic | 0.1 (0 to 0.3) | 4 (0.9 to 9.1) | 0.1 (0 to 0.2) | 1.3 (0.3 to 3.3) | -3.91 (-4 to -3.81) |
| Latvia | 0 (0 to 0.1) | 0.7 (0 to 2.7) | 0 (0 to 0) | 0.1 (0 to 0.4) | -6.38 (-8.38 to -4.34) |
| Lebanon | 0 (0 to 0.1) | 1.1 (0.3 to 2.3) | 0 (0 to 0) | 0.2 (0 to 0.5) | -5.68 (-5.83 to -5.52) |
| Lesotho | 0.1 (0 to 0.1) | 5.1 (2 to 10.5) | 0 (0 to 0.1) | 2.9 (1.1 to 5.7) | -1.91 (-2 to -1.83) |
| Liberia | 0 (0 to 0.1) | 2.7 (0.9 to 5.6) | 0 (0 to 0.1) | 1.4 (0.5 to 3.1) | -2.05 (-2.22 to -1.89) |
| Libya | 0 (0 to 0.1) | 1.1 (0.3 to 2.7) | 0 (0 to 0) | 0.2 (0 to 0.7) | -5.05 (-5.45 to -4.64) |
| Lithuania | 0 (0 to 0.1) | 0.6 (0 to 2.6) | 0 (0 to 0) | 0.1 (0 to 0.4) | -6.12 (-7.13 to -5.1) |
| Luxembourg | 0 (0 to 0) | 0.3 (0 to 0.7) | 0 (0 to 0) | 0.1 (0 to 0.3) | -3.88 (-4 to -3.76) |
| Madagascar | 0.2 (0 to 0.4) | 2.8 (0.9 to 5.8) | 0.2 (0 to 0.5) | 1.5 (0.4 to 3.4) | -2.13 (-2.28 to -1.98) |
| Malawi | 0.1 (0 to 0.2) | 2.1 (0.8 to 4.2) | 0.1 (0 to 0.3) | 1.1 (0.3 to 2.3) | -2.35 (-2.44 to -2.26) |
| Malaysia | 0.2 (0 to 0.4) | 1.2 (0.1 to 3.1) | 0.1 (0 to 0.2) | 0.2 (0 to 0.6) | -6.04 (-6.29 to -5.79) |
| Maldives | 0 (0 to 0) | 2.2 (0.4 to 5.3) | 0 (0 to 0) | 0.4 (0.1 to 0.9) | -6.15 (-6.36 to -5.95) |
| Mali | 0.3 (0.1 to 0.5) | 5.3 (2.2 to 9.9) | 0.3 (0.1 to 0.5) | 2.4 (1 to 4.7) | -2.6 (-2.8 to -2.4) |
| Malta | 0 (0 to 0) | 1.1 (0.6 to 2) | 0 (0 to 0) | 0.4 (0.2 to 0.8) | -3.32 (-3.46 to -3.18) |
| Marshall Islands | 0 (0 to 0) | 3.8 (0.4 to 11.8) | 0 (0 to 0) | 1.5 (0.1 to 6) | -3.11 (-3.33 to -2.89) |
| Mauritania | 0 (0 to 0.1) | 1.8 (0.4 to 4.1) | 0 (0 to 0) | 0.5 (0.1 to 1.4) | -4.18 (-4.35 to -4.01) |
| Mauritius | 0 (0 to 0) | 0.7 (0 to 2.2) | 0 (0 to 0) | 0.1 (0 to 0.5) | -5.85 (-6.47 to -5.22) |
| Mexico | 1.7 (0.8 to 3.1) | 3.1 (1.5 to 5.5) | 0.7 (0.3 to 1.4) | 0.6 (0.2 to 1.1) | -5.81 (-6.01 to -5.61) |
| Micronesia (Federated States of) | 0 (0 to 0) | 5.5 (0.9 to 15.2) | 0 (0 to 0) | 1.7 (0.2 to 6.1) | -4.02 (-4.08 to -3.95) |
| Monaco | 0 (0 to 0) | 0.1 (0 to 0.3) | 0 (0 to 0) | 0.1 (0 to 0.2) | -2.72 (-2.78 to -2.67) |
| Mongolia | 0.1 (0 to 0.1) | 5 (1.3 to 11.9) | 0 (0 to 0.1) | 1.5 (0.4 to 3.5) | -4.06 (-4.32 to -3.81) |
| Montenegro | 0 (0 to 0) | 0.2 (0 to 0.8) | 0 (0 to 0) | 0.1 (0 to 0.4) | -1.7 (-1.93 to -1.48) |
| Morocco | 0.5 (0.2 to 1.1) | 3 (1 to 6.2) | 0.3 (0.1 to 0.6) | 0.9 (0.3 to 1.9) | -4.08 (-4.25 to -3.91) |
| Mozambique | 0.2 (0.1 to 0.4) | 3 (1.3 to 6) | 0.2 (0.1 to 0.5) | 1.8 (0.7 to 3.5) | -1.81 (-1.9 to -1.72) |
| Myanmar | 1.1 (0.3 to 2.5) | 3.6 (1.1 to 7.9) | 0.5 (0.1 to 1.2) | 1 (0.2 to 2.2) | -4.51 (-4.68 to -4.34) |
| Namibia | 0 (0 to 0.1) | 2.8 (0.7 to 6) | 0 (0 to 0) | 0.9 (0.2 to 2.2) | -3.75 (-3.91 to -3.59) |
| Nauru | 0 (0 to 0) | 0.6 (0 to 3.2) | 0 (0 to 0) | 0.4 (0 to 2.2) | -1.59 (-1.77 to -1.41) |
| Nepal | 2.6 (1.1 to 5) | 21.4 (9.4 to 41.7) | 2.2 (0.9 to 4) | 9.2 (4.2 to 16.9) | -2.89 (-2.96 to -2.82) |
| Netherlands | 0 (0 to 0.1) | 0.2 (0 to 0.4) | 0 (0 to 0.1) | 0.1 (0 to 0.2) | -2.72 (-3.38 to -2.06) |
| New Zealand | 0.1 (0 to 0.1) | 1.9 (0.8 to 3.6) | 0 (0 to 0.1) | 0.5 (0.2 to 1) | -4.71 (-5.03 to -4.38) |
| Nicaragua | 0 (0 to 0.1) | 1.7 (0.8 to 3.4) | 0 (0 to 0.1) | 0.5 (0.2 to 1.1) | -4.43 (-4.72 to -4.14) |
| Niger | 0.2 (0.1 to 0.5) | 5.7 (2.4 to 12.1) | 0.3 (0.1 to 0.7) | 3.3 (1.4 to 6.6) | -1.89 (-2.1 to -1.69) |
| Nigeria | 1.5 (0.4 to 3.3) | 2.8 (0.9 to 6.2) | 1 (0.3 to 2.5) | 0.9 (0.3 to 2.1) | -3.8 (-3.93 to -3.67) |
| Niue | 0 (0 to 0) | 0.2 (0 to 1.3) | 0 (0 to 0) | 0.1 (0 to 0.5) | -3.6 (-3.7 to -3.49) |
| North Macedonia | 0 (0 to 0) | 0.9 (0.1 to 2.2) | 0 (0 to 0) | 0.2 (0 to 0.6) | -4.34 (-4.59 to -4.08) |
| Northern Mariana Islands | 0 (0 to 0) | 0.1 (0 to 0.6) | 0 (0 to 0) | 0 (0 to 0.3) | -3.18 (-3.3 to -3.06) |
| Norway | 0 (0 to 0) | 0.2 (0 to 0.5) | 0 (0 to 0) | 0.1 (0 to 0.2) | -4.05 (-4.47 to -3.64) |
| Oman | 0 (0 to 0) | 0.8 (0.3 to 1.9) | 0 (0 to 0) | 0.2 (0.1 to 0.3) | -5.48 (-5.79 to -5.17) |
| Pakistan | 11.7 (4.8 to 22.1) | 17.3 (7.5 to 31.7) | 13.4 (5.2 to 26.2) | 9.9 (4.3 to 18.3) | -1.84 (-2 to -1.68) |
| Palau | 0 (0 to 0) | 0.2 (0 to 1) | 0 (0 to 0) | 0.1 (0 to 0.4) | -3.36 (-3.43 to -3.3) |
| Palestine | 0 (0 to 0) | 1.2 (0.5 to 2.5) | 0 (0 to 0) | 0.3 (0.1 to 0.7) | -4.41 (-4.53 to -4.29) |
| Panama | 0 (0 to 0.1) | 1.9 (0.6 to 3.8) | 0 (0 to 0) | 0.4 (0.1 to 1.1) | -4.89 (-5.08 to -4.7) |
| Papua New Guinea | 0.1 (0 to 0.3) | 2.4 (0 to 9.8) | 0.1 (0 to 0.4) | 1.5 (0 to 6.3) | -1.56 (-1.65 to -1.47) |
| Paraguay | 0 (0 to 0.1) | 1.3 (0.4 to 2.8) | 0 (0 to 0.1) | 0.6 (0.2 to 1.4) | -2.8 (-3.08 to -2.53) |
| Peru | 0.2 (0 to 0.5) | 1.3 (0.4 to 2.8) | 0.2 (0 to 0.4) | 0.5 (0.1 to 1.2) | -3.41 (-3.8 to -3.01) |
| Philippines | 0.3 (0.1 to 0.8) | 0.6 (0.1 to 1.5) | 0.5 (0.1 to 1.6) | 0.5 (0.1 to 1.5) | -0.35 (-0.63 to -0.07) |
| Poland | 1 (0.2 to 2.2) | 2.3 (0.4 to 5.2) | 0.2 (0 to 0.4) | 0.3 (0 to 0.6) | -7.33 (-7.63 to -7.04) |
| Portugal | 0.2 (0.1 to 0.4) | 1.6 (0.7 to 3.1) | 0.1 (0 to 0.2) | 0.3 (0.1 to 0.6) | -5.71 (-6.13 to -5.28) |
| Puerto Rico | 0 (0 to 0) | 0.2 (0 to 0.5) | 0 (0 to 0) | 0 (0 to 0.2) | -4.86 (-5.16 to -4.57) |
| Qatar | 0 (0 to 0) | 0.5 (0 to 1.4) | 0 (0 to 0) | 0.1 (0 to 0.3) | -5.02 (-5.27 to -4.76) |
| Republic of Korea | 0.1 (0 to 0.2) | 0.3 (0.1 to 0.6) | 0 (0 to 0.1) | 0.1 (0 to 0.1) | -5.43 (-5.57 to -5.3) |
| Republic of Moldova | 0.1 (0 to 0.2) | 1.5 (0 to 4.6) | 0 (0 to 0) | 0.3 (0 to 0.9) | -5.39 (-6.22 to -4.55) |
| Romania | 0.5 (0.1 to 1.3) | 2 (0.3 to 4.7) | 0.1 (0 to 0.2) | 0.3 (0 to 0.6) | -6.63 (-6.93 to -6.32) |
| Russian Federation | 1.1 (0 to 4.1) | 0.6 (0 to 2.3) | 0.3 (0 to 0.9) | 0.1 (0 to 0.4) | -5.53 (-6.46 to -4.6) |
| Rwanda | 0.1 (0 to 0.2) | 3 (1 to 6) | 0.1 (0 to 0.2) | 0.8 (0.2 to 2) | -4.32 (-4.44 to -4.2) |
| Saint Kitts and Nevis | 0 (0 to 0) | 1.3 (0.2 to 3.3) | 0 (0 to 0) | 0.2 (0 to 0.5) | -6.74 (-7.16 to -6.32) |
| Saint Lucia | 0 (0 to 0) | 3.1 (0.9 to 6.4) | 0 (0 to 0) | 0.6 (0.1 to 1.5) | -5.56 (-5.9 to -5.21) |
| Saint Vincent and the Grenadines | 0 (0 to 0) | 3.9 (1.7 to 7.3) | 0 (0 to 0) | 1 (0.3 to 2.3) | -4.68 (-4.91 to -4.45) |
| Samoa | 0 (0 to 0) | 0.8 (0 to 3.6) | 0 (0 to 0) | 0.4 (0 to 2) | -2.3 (-2.47 to -2.12) |
| San Marino | 0 (0 to 0) | 0.4 (0 to 1) | 0 (0 to 0) | 0.2 (0 to 0.6) | -2.13 (-2.25 to -2) |
| Sao Tome and Principe | 0 (0 to 0) | 2.6 (0.7 to 5.6) | 0 (0 to 0) | 1.3 (0.3 to 3.1) | -2.29 (-2.51 to -2.07) |
| Saudi Arabia | 0.2 (0.1 to 0.5) | 2 (0.7 to 5) | 0.1 (0 to 0.2) | 0.3 (0.1 to 0.7) | -6.11 (-6.34 to -5.88) |
| Senegal | 0.1 (0 to 0.2) | 2 (0.6 to 4.1) | 0.1 (0 to 0.2) | 0.9 (0.2 to 2) | -2.9 (-3.42 to -2.37) |
| Serbia | 0.1 (0 to 0.1) | 0.5 (0.1 to 1.2) | 0 (0 to 0.1) | 0.2 (0 to 0.4) | -3.21 (-3.5 to -2.92) |
| Seychelles | 0 (0 to 0) | 1.1 (0 to 3) | 0 (0 to 0) | 0.1 (0 to 0.5) | -7.18 (-7.37 to -6.98) |
| Sierra Leone | 0.1 (0 to 0.1) | 2.6 (0.8 to 5.2) | 0.1 (0 to 0.1) | 1.4 (0.5 to 2.9) | -1.99 (-2.07 to -1.9) |
| Singapore | 0 (0 to 0) | 0.7 (0.1 to 1.5) | 0 (0 to 0) | 0.1 (0 to 0.1) | -8.11 (-8.39 to -7.82) |
| Slovakia | 0 (0 to 0.1) | 0.4 (0 to 1.1) | 0 (0 to 0) | 0.1 (0 to 0.4) | -3.54 (-4.15 to -2.92) |
| Slovenia | 0 (0 to 0) | 0.5 (0 to 1.5) | 0 (0 to 0) | 0.2 (0 to 0.5) | -3.79 (-3.99 to -3.6) |
| Solomon Islands | 0 (0 to 0) | 7 (0.8 to 21.3) | 0 (0 to 0.1) | 4.1 (0.5 to 13.5) | -1.84 (-1.97 to -1.7) |
| Somalia | 0.2 (0.1 to 0.4) | 5.3 (2.1 to 10.8) | 0.3 (0.1 to 0.7) | 3.3 (1.1 to 7.6) | -1.62 (-1.67 to -1.57) |
| South Africa | 0.4 (0 to 1.1) | 1.3 (0.2 to 3.3) | 0.3 (0 to 0.9) | 0.5 (0.1 to 1.5) | -3.08 (-3.76 to -2.4) |
| South Sudan | 0.1 (0 to 0.1) | 2.1 (0.6 to 4.6) | 0.1 (0 to 0.1) | 1.2 (0.3 to 2.7) | -2.05 (-2.12 to -1.98) |
| Spain | 0.7 (0.2 to 1.4) | 1.4 (0.4 to 2.8) | 0.4 (0.1 to 0.9) | 0.4 (0.1 to 0.9) | -4.39 (-4.53 to -4.25) |
| Sri Lanka | 0.1 (0 to 0.2) | 0.4 (0 to 1.3) | 0 (0 to 0.1) | 0.1 (0 to 0.3) | -4.94 (-5.34 to -4.54) |
| Sudan | 1.1 (0.5 to 2.4) | 9.3 (4.1 to 21.2) | 0.7 (0.3 to 1.5) | 3 (1.4 to 6.5) | -3.87 (-3.95 to -3.79) |
| Suriname | 0 (0 to 0) | 1.5 (0.4 to 3.4) | 0 (0 to 0) | 0.5 (0.1 to 1.3) | -4.12 (-4.95 to -3.29) |
| Sweden | 0 (0 to 0) | 0.1 (0 to 0.3) | 0 (0 to 0) | 0 (0 to 0.1) | -3.81 (-4.15 to -3.47) |
| Switzerland | 0 (0 to 0.1) | 0.3 (0 to 0.8) | 0 (0 to 0) | 0.1 (0 to 0.2) | -5.45 (-5.69 to -5.22) |
| Syrian Arab Republic | 0.6 (0.3 to 1.2) | 8.6 (3.8 to 16.4) | 0.1 (0 to 0.3) | 1 (0.4 to 2) | -7.2 (-7.61 to -6.79) |
| Taiwan (Province of China) | 0.2 (0 to 0.4) | 1.2 (0.3 to 2.4) | 0.1 (0 to 0.1) | 0.2 (0 to 0.4) | -6.65 (-6.9 to -6.39) |
| Tajikistan | 0.2 (0.1 to 0.5) | 6.3 (2.1 to 12.4) | 0.1 (0 to 0.3) | 2.1 (0.7 to 4.3) | -3.65 (-3.97 to -3.32) |
| Thailand | 0.4 (0 to 1.4) | 0.9 (0 to 2.8) | 0.1 (0 to 0.3) | 0.1 (0 to 0.3) | -8.04 (-8.39 to -7.7) |
| Timor-Leste | 0 (0 to 0) | 2.8 (0.6 to 6.4) | 0 (0 to 0) | 1.2 (0.3 to 3) | -2.87 (-3.15 to -2.58) |
| Togo | 0 (0 to 0.1) | 2.1 (0.6 to 4.5) | 0 (0 to 0.1) | 1 (0.2 to 2.3) | -2.59 (-2.75 to -2.43) |
| Tokelau | 0 (0 to 0) | 1.3 (0.2 to 4.7) | 0 (0 to 0) | 0.4 (0 to 1.7) | -4.02 (-4.07 to -3.97) |
| Tonga | 0 (0 to 0) | 0.4 (0 to 1.6) | 0 (0 to 0) | 0.2 (0 to 0.8) | -3.01 (-3.17 to -2.85) |
| Trinidad and Tobago | 0 (0 to 0) | 0.4 (0 to 1.6) | 0 (0 to 0) | 0.1 (0 to 0.5) | -5.01 (-5.4 to -4.62) |
| Tunisia | 0.1 (0 to 0.2) | 1.5 (0.6 to 3.1) | 0 (0 to 0.1) | 0.4 (0.1 to 0.8) | -4.63 (-4.7 to -4.56) |
| Turkey | NA | NA | NA | NA | NA |
| Turkmenistan | 0 (0 to 0.1) | 1.4 (0.1 to 4.1) | 0 (0 to 0.1) | 0.4 (0 to 1.5) | -3.75 (-4.94 to -2.54) |
| Tuvalu | 0 (0 to 0) | 1.8 (0 to 7.5) | 0 (0 to 0) | 0.6 (0 to 3.1) | -3.54 (-3.61 to -3.46) |
| Uganda | 0.2 (0.1 to 0.4) | 2.1 (0.8 to 4.4) | 0.2 (0 to 0.5) | 1 (0.3 to 2.3) | -2.64 (-2.78 to -2.5) |
| Ukraine | 0.1 (0 to 0.6) | 0.2 (0 to 0.8) | 0.1 (0 to 0.3) | 0.1 (0 to 0.4) | -2.4 (-3.3 to -1.5) |
| United Arab Emirates | 0 (0 to 0.1) | 3 (0.7 to 7.8) | 0 (0 to 0.1) | 0.6 (0.1 to 1.8) | -5.45 (-5.77 to -5.12) |
| United Kingdom | 0.3 (0 to 0.8) | 0.4 (0 to 0.9) | 0.1 (0 to 0.2) | 0.1 (0 to 0.2) | -5.7 (-5.86 to -5.53) |
| United Republic of Tanzania | 0.2 (0.1 to 0.5) | 1.8 (0.7 to 3.6) | 0.2 (0 to 0.6) | 0.7 (0.2 to 1.7) | -3.17 (-3.27 to -3.06) |
| United States of America | 1.9 (0.5 to 4) | 0.6 (0.2 to 1.3) | 0.9 (0.1 to 2.1) | 0.1 (0 to 0.4) | -4.92 (-5.04 to -4.79) |
| United States Virgin Islands | 0 (0 to 0) | 0.3 (0 to 0.8) | 0 (0 to 0) | 0.1 (0 to 0.3) | -4.45 (-4.73 to -4.16) |
| Uruguay | 0 (0 to 0) | 0.7 (0.1 to 1.4) | 0 (0 to 0) | 0.3 (0.1 to 0.7) | -2.57 (-2.93 to -2.22) |
| Uzbekistan | 0.5 (0 to 1.3) | 3.2 (0.3 to 8.4) | 0.4 (0 to 1.3) | 1.5 (0.1 to 4) | -2.49 (-3.01 to -1.98) |
| Vanuatu | 0 (0 to 0) | 2.7 (0.2 to 9.3) | 0 (0 to 0) | 1.8 (0.1 to 6.9) | -1.42 (-1.74 to -1.11) |
| Venezuela (Bolivarian Republic of) | 0.1 (0 to 0.3) | 1 (0.3 to 2.2) | 0.1 (0 to 0.2) | 0.2 (0.1 to 0.6) | -4.87 (-5.12 to -4.63) |
| Viet Nam | 0.6 (0.1 to 1.5) | 1.3 (0.2 to 3.1) | 0.4 (0.1 to 0.9) | 0.4 (0.1 to 0.9) | -4.27 (-4.35 to -4.19) |
| Yemen | 0.8 (0.4 to 1.6) | 12 (5.9 to 25.7) | 1 (0.4 to 2) | 5.5 (2.6 to 11) | -2.67 (-2.83 to -2.51) |
| Zambia | 0.1 (0 to 0.1) | 1.6 (0.5 to 3.6) | 0.1 (0 to 0.2) | 0.8 (0.2 to 1.9) | -2.29 (-2.55 to -2.04) |
| Zimbabwe | 0.2 (0.1 to 0.5) | 4.1 (1.7 to 7.4) | 0.3 (0.1 to 0.7) | 3 (0.9 to 6.6) | -0.99 (-1.12 to -0.86) |
